# Improve the model of disease subtype heterogeneity by leveraging external summary data

**DOI:** 10.1371/journal.pcbi.1011236

**Published:** 2023-07-12

**Authors:** Sheng Fu, Mark P. Purdue, Han Zhang, Jing Qin, Lei Song, Sonja I. Berndt, Kai Yu

**Affiliations:** 1 Division of Cancer Epidemiology and Genetics, National Cancer Institute, Bethesda, Maryland, United States of America; 2 National Institute of Allergy and Infectious Diseases, National Institutes of Health, Bethesda, Maryland, United States of America; Yale, UNITED STATES

## Abstract

Researchers are often interested in understanding the disease subtype heterogeneity by testing whether a risk exposure has the same level of effect on different disease subtypes. The polytomous logistic regression (PLR) model provides a flexible tool for such an evaluation. Disease subtype heterogeneity can also be investigated with a case-only study that uses a case-case comparison procedure to directly assess the difference between risk effects on two disease subtypes. Motivated by a large consortium project on the genetic basis of non-Hodgkin lymphoma (NHL) subtypes, we develop PolyGIM, a procedure to fit the PLR model by integrating individual-level data with summary data extracted from multiple studies under different designs. The summary data consist of coefficient estimates from working logistic regression models established by external studies. Examples of the working model include the case-case comparison model and the case-control comparison model, which compares the control group with a subtype group or a broad disease group formed by merging several subtypes. PolyGIM efficiently evaluates risk effects and provides a powerful test for disease subtype heterogeneity in situations when only summary data, instead of individual-level data, is available from external studies due to various informatics and privacy constraints. We investigate the theoretic properties of PolyGIM and use simulation studies to demonstrate its advantages. Using data from eight genome-wide association studies within the NHL consortium, we apply it to study the effect of the polygenic risk score defined by a lymphoid malignancy on the risks of four NHL subtypes. These results show that PolyGIM can be a valuable tool for pooling data from multiple sources for a more coherent evaluation of disease subtype heterogeneity.

## Author summary

Researchers usually classify a disease condition into subtypes with different progression patterns and treatment responses. Multiple studies often investigate a complex disease, but not all of them consider the same set of subtypes. In addition, due to various informatics and privacy constraints, it can be challenging to pool individual data across all studies for more efficient analyses. On the other hand, summarized data, such as those generated from genetic association studies, can be easily accessed. We develop PolyGIM, a flexible statistical framework to integrate detailed individual-level data with summary data from multiple sources to comprehensively assess the risk effect on different disease subtypes. We use PolyGIM to understand the genetic basis underlying four major non-Hodgkin lymphoma subtypes.

## 1 Introduction

The polytomous logistic regression (PLR) model is a standard approach to modeling the effects of risk factors on a multicategory outcome [1]. It can be applied to a retrospectively sampled case-control study with one control group and several case groups, such as those defined by different disease subtypes [2]. The PLR model provides a consistent estimate of subtype-specific odds ratio associated with risk exposure [3]. Besides subtype-specific odds ratio, researchers are often interested in understanding the disease subtype heterogeneity by testing whether a risk exposure has the same effect on different disease subtypes [4–6]. Demonstrated evidence of the non-uniform exposure effect would suggest that different etiologic mechanisms cause some disease subtypes.

Leveraging robust findings on genetic associations from large-scale genome-wide association studies (GWAS), recent studies have been using the polygenic risk score (PRS) [7–9] to dissect the complex genetic architecture underlying different disease subtypes [10–14]. We calculate PRS as a weighted average of genotypes on a set of trait-associated genetic markers, i.e., single nucleotide polymorphisms (SNPs), with weights being their effect sizes estimated from existing large-scale GWAS [9, 15, 16]. The PRS provides an estimate of the overall genetic influence on specific traits and can be used as an effective instrument to dissect the genetic architecture underlying different disease subtypes.

Multiple studies are often conducted to investigate a complex disease but do not consider the same subtypes. In addition, due to various informatics and privacy constraints, it can be challenging to share individual data among studies for a more powerful pooled analysis. Our method is motivated by an InterLymph Consortium project [17]. We intend to study the non-Hodgkin lymphoma (NHL) subtype heterogeneity using data generated from multiple GWAS on four major NHL subtypes, known as hronic lymphocytic leukemia (CLL), diffuse large B-cell lymphoma (DLBCL), follicular lymphoma (FL), and marginal zone lymphoma (MZL) [18–23]. NHL is the most common hematological malignancy and has multiple subtypes with distinct morphologic, genetic, and clinical features [24, 25]. In particular, we are interested in evaluating whether the PRS of a lymphoid malignancy, such as Hodgkin lymphoma, exhibits different effects on the four considered NHL subtypes. In this project, we have individual-level genotype data from one study (the internal study) that consists of subjects from each of the four NHL subtype groups and controls. Instead of having individual-level data, we obtain the typical SNP-level summary data from the other seven GWAS (external studies). The SNP-level summary data consist of estimated regression coefficients, each representing an SNP’s marginal effect on a specific NHL subtype. This type of GWAS summary statistics is usually accessible from public databases and has become a valuable resource for future genetic studies [26–29]. Among those seven external case-control studies, one study has cases from three NHL subtype groups, and each of the remaining studies consists of cases of a unique subtype. We aim to integrate individual-level and summary data from all eight GWAS for a more efficient evaluation of the PRS effect and a more powerful heterogeneity test on whether the PRS has a common effect on different NHL subtypes.

Like SNP-level summary data, we consider model-based summary data, which consists of estimated coefficients from working models established by external studies. Working models can be quite different from the target model (i.e., the underlying risk model assumed by the internal study) and thus misspecified. In the NHL study mentioned above, SNP-level summary data are calculated from marginal models, each of which assesses the effect of a single SNP on an outcome. In contrast, the target model is the PRS effect model, which models the effect of a composite genotype score defined by a set of SNPs. In some applications, summary data consist of the estimated coefficient of a risk exposure measured in a different scale (e.g., in log scale) from the one used in the target model. Therefore, in general, we cannot use a standard meta-analysis procedure to pool summary data with estimates from the internal study to improve the inference on the target model.

A few procedures have been developed to integrate model-based summary data with individual-level data, but they mainly focused on studies with prospectively collected samples [30–37]. [38] recently developed an empirical likelihood approach to account for the sampling bias in the case-control study design to synthesize data from retrospective case-control studies. They focused on a binary outcome with a logistic regression model as the underlying risk model.

Here we expand the approach of [38] to multicategory outcomes and develop an efficient procedure to fit the PLR model using individual-level and summary data. Unlike the procedure of [38], the new approach allows different types of outcomes (i.e., one is multicategory, and the other is binary) to be studied by internal and external studies. The binary outcome considered by an external study can be any dichotomized version of the original multicategory outcome. For example, an external study can be a case-control study that adopts a logistic regression model to evaluate a risk exposure’s effect (odds ratio) on a specific disease subtype or a group of several disease subtypes. In another scenario, an external study might collect only cases (i.e., a case-only study) and uses a logistic regression model to directly assess the difference between the effects of a risk factor on two disease subtypes. We also implement the new method into a user-friendly R package, PolyGIM, which can incorporate summary data from multiple external studies with possibly overlapping subjects. We conduct theoretical and simulation studies to demonstrate the advantage of the proposed procedure. We apply the new procedure to the NHL study mentioned above study.

## 2 Method

### 2.1 Setup

Let’s assume that we have a case-control study of a multicategory outcome *Y* and a set of covariates ***X***. The outcome *Y* takes integer value from 0 to *K*, with *Y* = 0 for controls, and *Y* > 0 for other *K* different outcomes. For example, in the motivation example we consider a case-control study of NHL with four subtypes. We can denote individual-level data from this study (called internal study) as {***X***_*i*_, *Y*_*i*_, *i* = 1, …, *n*}, with *n*_*k*_ subjects having outcome *Y* = *k*, and $n=\sum_{k=0}^{K}n_{k}$. In the following discussion, we refer *Y* = 1, …, *K* as different disease subtypes. We assume the following prospective PLR model as the underlying model for the effect of ***X*** on *Y*,

$$\begin{array}{c} \text{log}\{\frac{P(Y=k|X)}{P(Y=0|X)}\}=\omega_{k}+M_{k}\left(X;\theta_{k}\right),\;k=1,\ldots ,K, \end{array}$$ (1)

where *ω*_*k*_ is the intercept and *M*_*k*_(***X***; ***θ***_*k*_) is a given function, such as *M*_*k*_(***X***; ***θ***_*k*_) = ***X***^⊤^***θ***_*k*_, with ***θ***_*k*_ being the vector of parameters of interest. In the NHL example, we have *M*_*k*_(***X***; *θ*_*k*_) = *θ*_*k*_ ⋅ *S*(***X***), with *S*(***X***) being the PRS. *S*(***X***) is defined as ∑_*i*_*w*_*i*_*X*_*i*_, with *X*_*i*_ being the genotype at the *k*-th SNP, and *w*_*i*_ being the weight defined by previous GWAS. Let $\theta ={\left(\theta_{1}^{⊤},\ldots ,\theta_{K}^{⊤}\right)}^{⊤}$ be the collection of all coefficients. We are interested in estimating ***θ*** or comparing ***θ***_*k*_ among different disease subtypes.

**Fig 1:**
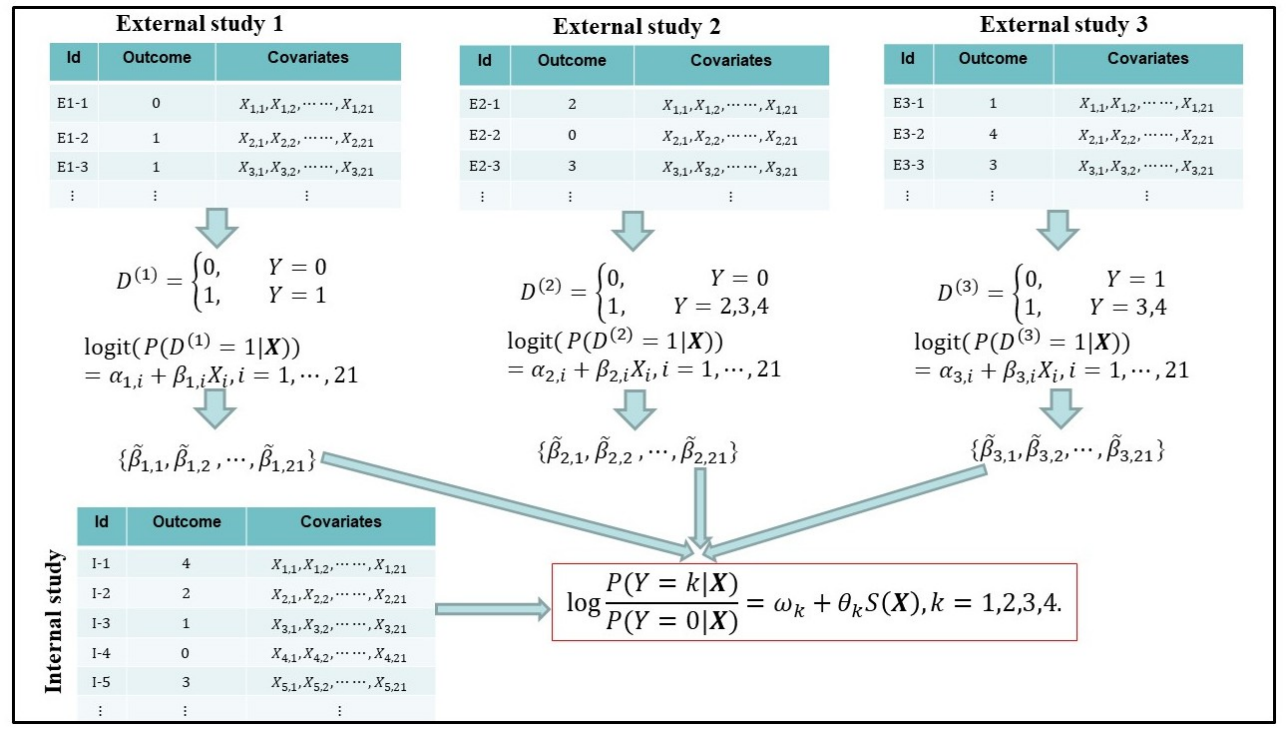
Illustration of the PolyGIM framework. We consider a setting similar to the NHL study. The outcome *Y* takes values from {0, 1, 2, 3, 4}. The set of covariates ***X*** comprises measures on 21 genetic markers (SNPs). We have individual-level data (*Y*, ***X***) from the internal study and summary data from three external studies. Summary data $\left\{{\tilde{\beta }}_{k,i},\;i=1,\ldots ,21;\;k=1,2,3\right\}$ consists of coefficient estimates from marginal logistic regression models established by the *k*-th external study (*k* = 1, 2, 3), with the binary outcome (*D*^(*k*)^) of each external model defined by *Y*. The goal is to fit the polytomous regression model for the PRS effect given in the red box using individual-level and summary data.

Beside data from the internal study, we have summary data from multiple external studies. In the NHL example, we consider the PRS defined by a set of 21 SNPs [39]. For each of those SNPs, we have SNP-level summary statistics from seven external studies. The SNP-level summary data is the estimated SNP’s marginal association (the regression coefficient) with one NHL subtype based on a standard logistic regression model (called the working model). Therefore, for an external study consisting of three different NHL subtype cases and controls, its summary data consists of 21 × 3 estimated regression coefficients, each of which is derived from a separate working model. The goal is to fit the PLR model (1) using individual-level and summary data and to utilize the fitted model for more efficient inference of ***θ***_*k*_ and disease subtype heterogeneity. An illustration of the proposed integration framework is given in Fig 1. Before considering this complicated scenario, we first present the methodology assuming that there is one external study, and that the summary data is derived from a single working model. We will describe the method under the more general setting in Section 2.7.

More specifically, we consider summary data consisting of regression coefficient estimates derived from a working model of a binary outcome *D*. The binary outcome *D* is derived from of the multicategory outcome *Y*. The working model is assumed to be a standard logistic regression model. Several versions of *D* can occur in practice. For example, a working model can treat several disease subtypes as one broad disease group, that is, *D* = 0 if *Y* = 0, and *D* = 1 if *Y* ∈ {*c*_1_, …, *c*_*L*_}, with 1 ≤ *c*_*l*_ ≤ *K*, 1 ≤ *l* ≤ *L*, where *L* is the total number of disease subtypes under study. We call this type of working model the grouped case-control (GC) model. We further assume the number of cases within each subtype group is known in the GC model. In some cases, such as in the NHL study, the GC model can consider just one subtype (i.e., *L* = 1). Another version of *D* arises from a case-only study, in which a logistic regression model is used to evaluate whether a risk factor has the same effect on two disease subtypes *c*_1_ and *c*_2_. We call this the case-case (CC) comparison model, with *D* = 0 for *Y* = *c*_1_, and *D* = 1 for *Y* = *c*_2_.

### 2.2 Likelihood for the internal study

Observations from the internal case-control study can be retrospectively sampled conditioning on the outcome *Y* from a source population. For some other case-control studies, cases are sampled from *P*(***X***|*Y* > 0), with their disease subtypes classified after the enrollment. As long as the sampling criterion is independent of ***X***, we can consider both types of case-control studies as stratified samples from *P*(***X***|*Y* = 0) and *P*(***X***|*Y* = *k*), *k* = 1, …, *K*.

We consider the retrospective likelihood for the case-control data as this framework is convenient for incorporating external summary data. Let *π*_*k*_ = *P*(*Y* = *k*) and $\tau_{k}=\omega_{k}+\text{log}\left\{\left(1-\sum_{i=1}^{K}\pi_{i}\right)/\pi_{k}\right\}$, *k* = 1, …, *K*. We define the vector ***τ*** = (*τ*_1_, …, *τ*_*K*_)^⊤^. By Bayes’ rule, model (1) has the following equivalent retrospective representation,

$$\begin{array}{c} P\left(X|Y=k\right)=P\left(X|Y=0\right)\Delta_{k}\left(X;\xi \right),\;k=1,\ldots ,K, \end{array}$$ (2)

where Δ_*k*_(***X***; ***ξ***) = exp{*τ*_*k*_ + *M*_*k*_(***X***; ***θ***_*k*_)}, *k* = 1, …, *K*, with ***ξ*** = (***τ***^⊤^, ***θ***^⊤^)^⊤^. We denote ξ* = (τ*^⊤^, θ*^⊤^)^⊤^ as the true population value of ***ξ***.

We use (2) to form the likelihood of observing ***X*** given *Y*. Denote $P=\{p_{i}\;≜\;P\left(X_{i}|Y=0\right),\;i=1,\ldots ,n\}$ as the empirical distribution of *P*(***X***|*Y* = 0) supported on samples from the internal study, and 1(·) as the indicator function. We can estimate ***ξ*** by maximizing the following empirical log-likelihood,

$$\begin{array}{c} \sum_{i=1}^{n}\;\text{log}\;p_{i}+\sum_{i=1}^{n}\sum_{k=1}^{K}1\left(Y_{i}=k\right)\cdot \text{log}\left\{\Delta_{k}\left(X_{i};\xi \right)\right\}, \end{array}$$ (3)

subject to constraints $\sum_{i=1}^{n}p_{i}=1,\;p_{i}\geq 0$ and $\sum_{i=1}^{n}p_{i}\Delta_{k}\left(X_{i};\xi \right)=1$ for *k* = 1, …, *K*. Using the method of Lagrange multipliers to profile out P, we can infer ***ξ*** by finding the stationary point of the following profile log-likelihood,

$$\begin{array}{c} \ell_{1}\left(\xi \right)=\sum_{i=1}^{n}\sum_{k=1}^{K}1\left(Y_{i}=k\right)\cdot \text{log}\left\{\Delta_{k}\left(X_{i};\xi \right)\right\}-\sum_{i=1}^{n}\text{log}\{1+\sum_{k=1}^{K}\rho_{k}\Delta_{k}\left(X_{i};\xi \right)\}, \end{array}$$ (4)

where *ρ*_*k*_ = *n*_*k*_/*n*_0_ for *k* = 1, …, *K*. Let ${\hat{\xi }}_{mle}={\text{arg}\;\text{max}}_{\xi }\;\ell_{1}\left(\xi \right)$ be the maximum likelihood estimate (MLE) based on the internal data. When *M*_*k*_(***X***; ***θ***_*k*_) = ***X***^⊤^***θ***_*k*_(*k* = 1, …, *K*), we can follow [40] to show that the empirical likelihood estimate of ***θ*** derived from (4) is exactly the same as the maximum likelihood estimate based on the standard prospective likelihood function specified by (1).

### 2.3 Properties of summary data

Here we will show the property of the summary data and its relationship with ***θ***, the parameter of interest. We assume that the external study consists of observations {***X***_*i*_, *D*_*i*_, *i* = *n* + 1, …, *n* + *N*}, representing stratified samples from *P*(***X***|*D* = 0) and *P*(***X***|*D* = 1). Suppose a standard logistic regression model is used as the working model to study the effect of ***X*** on *D*. We can represent this model by its equivalent retrospective formation as,

$$\begin{array}{c} P\left(X|D=1\right)=P\left(X|D=0\right)\text{exp}\{\alpha_{0}+m\left(X;\alpha_{1},\beta \right)\}, \end{array}$$ (5)

with *P*(***X***|*D* = 1) and *P*(***X***|*D* = 0) being distributions of ***X*** in the two groups. This model can be misspecified and thus inconsistent with (2). In model (5), all unknown parameters are divided into two parts, $\alpha ={\left(\alpha_{0},\alpha_{1}^{⊤}\right)}^{⊤}$ and ***β***. We use ***α*** to represent the set of nuisance parameters whose estimates are not given as part of the summary data. Note that the intercept term *α*_0_ is always assumed to be part of nuisance parameters. The summary data only consists of the estimate of ***β***.

Let *N*_0_ and *N*_1_ be sample sizes in groups *D* = 0 and *D* = 1, respectively. Based on the working model (5), the log-likelihood function of the external study is

$$\ell_{2}\left(\alpha ,\beta \right)=\sum_{i=n+1}^{n+N}D_{i}\;\text{log}\;\left\{\delta \left(X_{i};\alpha ,\beta \right)\right\}-\sum_{i=n+1}^{n+N}\text{log}\left\{1+\rho \delta \left(X_{i};\alpha ,\beta \right)\right\},$$

where *ρ* = *N*_1_/*N*_0_ and $\delta \left(X;\alpha ,\beta \right)\;≜\;\text{exp}\{\alpha_{0}+m\left(X;\alpha_{1},\beta \right)\}$. $\left(\tilde{\alpha },\tilde{\beta }\right)$ is the solution of the following estimating equation,

$$\begin{array}{c} \frac{\partial \ell_{2}\left(\alpha ,\beta \right)}{\partial (\alpha ,\beta )}=\sum_{i=n+1}^{n+N}\{D_{i}-\frac{\rho \delta (X_{i};\alpha ,\beta )}{1+\rho \delta (X_{i};\alpha ,\beta )}\}\frac{\partial \;\text{log}\;\delta (X_{i};\alpha ,\beta )}{\partial (\alpha ,\beta )}=0. \end{array}$$ (6)

Note that $\tilde{\beta }$ is the same as the estimate from the standard package for the logistic regression model. For simplicity, let $\phi_{0}\left(X;\alpha ,\beta \right)=-\frac{\rho \delta (X;\alpha ,\beta )}{1+\rho \delta (X;\alpha ,\beta )}\frac{\partial \;\text{log}\;\delta (X;\alpha ,\beta )}{\partial (\alpha ,\beta )}$ and $\phi_{1}\left(X;\alpha ,\beta \right)=\frac{1}{1+\rho \delta (X;\alpha ,\beta )}\frac{\partial \;\text{log}\;\delta (X;\alpha ,\beta )}{\partial (\alpha ,\beta )}$. According to [41], $\left(\tilde{\alpha },\tilde{\beta }\right)$ is a consistent estimate of (α*, β*), which satisfies the following stochastic constraint equation

$$\begin{array}{c} E_{D=0}\left[\varphi_{0}\left(X;\alpha ,\beta \right)\right]+\rho E_{D=1}\left[\varphi_{1}\left(X;\alpha ,\beta \right)\right]=0, \end{array}$$ (7)

where $E_{D=0}$ and $E_{D=1}$ are expectations over *P*(***X***|*D* = 0) and *P*(***X***|*D* = 1), respectively.

The estimate $\tilde{\beta }$ depends on how *D* is derived from *Y*. As mentioned in Section 2.1, two types of binary outcomes are typically encountered. One is used in the CC model, and the other is used in the GC model.

The CC model compares the distribution of ***X*** between disease subtype groups *c*_0_ and *c*_1_, *c*_0_ ≠ *c*_1_ ∈ {1, …, *K*}. Its binary outcome is defined as *D* = *D*_*CC*_, with *D*_*CC*_ = 0 if *Y* = *c*_0_, and *D*_*CC*_ = 1 if *Y* = *c*_1_. The CC model is commonly used for the study of disease subtype heterogeneity. Let ***μ*** = (***τ***^⊤^, ***θ***^⊤^, ***α***^⊤^, ***β***^⊤^)^⊤^ be the vector of all unknown parameters, and μ* = (τ*^⊤^, θ*^⊤^, α*^⊤^, β*^⊤^)^⊤^ be their true population values. Notice that $P\left(X|D_{CC}=0\right)=P\left(X|Y=0\right)\Delta_{c_{0}}\left(X;\xi \right)$ and $P\left(X|D_{CC}=1\right)=P\left(X|Y=0\right)\Delta_{c_{1}}\left(X;\xi \right)$ due to (2), we can express (7) as

$$\begin{array}{c} E_{Y=0}\left[g\left(X;\mu^{*}\right)\right]=0, \end{array}$$ (8)

with

$$g\left(X;\mu \right)=\varphi_{0}\left(X;\alpha ,\beta \right)\Delta_{c_{0}}\left(X;\xi \right)+\rho \varphi_{1}\left(X;\alpha ,\beta \right)\Delta_{c_{1}}\left(X;\xi \right),$$

where $E_{Y=0}$ is the expectation of over *P*(***X***|*Y* = 0). The asymptotic distribution of $\left(\tilde{\alpha },\tilde{\beta }\right)$ is given by

$$\begin{array}{c} \sqrt{N}[\begin{array}{c} \tilde{\alpha }-\alpha^{*}\\ \tilde{\beta }-\beta^{*} \end{array}]\overset{\;d\;}{\to }N\left(0,A^{-1}BA^{-1}\right), \end{array}$$ (9)

with **A** and **B** shown in S1 Appendix. Based on (9), we know $\text{Cov}\left(\tilde{\beta }\right)=\frac{1}{N}\Sigma_{0}$, where **Σ**_0_ = (**A**^−1^**B** **A**^−1^)_***β*** ***β***_ is the submatrix of **A**^−1^**B** **A**^−1^ corresponding to ***β***. Notice that **A** and **B** are determined by the expectation defined by *P*(***X***|*Y* = 0). We will provide an empirical likelihood based estimate of *P*(***X***|*Y* = 0) later. According to (9), $\tilde{\beta }$ is a consistent estimate of β*, whose relationship with θ*, the parameter of interest, is governed by (8). Therefore, summary data $\tilde{\beta }$ contains information on θ*.

In the GC model, one or several disease subtypes are considered as one broad case group. The binary outcome is defined as *D* = *D*_*GC*_, with *D*_*GC*_ = 0 if *Y* = 0, and *D*_*GC*_ = 1 if *Y* belongs to {*c*_1_, *c*_2_, …, *c*_*L*_}. We further assume that the proportion of cases belonging to each disease subtype *k* is known, and denote it as *q*_*k*_(*k* = 1, …, *K*). Note that some *q*_*k*_s can be zero for studies that do not collect cases with certain disease subtypes. Based on (2), we have

$$P\left(X|D_{GC}=1\right)=\sum_{k=1}^{K}q_{k}P\left(X|Y=k\right)=P\left(X|Y=0\right)\sum_{k=1}^{K}q_{k}\Delta_{k}\left(X;\xi \right).$$

Then (8) still applies with ***g***(***X***; ***μ***) defined as

$$g\left(X;\mu \right)=\varphi_{0}\left(X;\alpha ,\beta \right)+\rho \varphi_{1}\left(X;\alpha ,\beta \right)\sum_{k=1}^{K}q_{k}\Delta_{k}\left(X,\xi \right).$$

Furthermore, the asymptotic distribution of $\left(\tilde{\alpha },\tilde{\beta }\right)$ takes the same form as (9), where **A** and **B** are defined in S1 Appendix. We can obtain $\text{Cov}(\tilde{\beta })$ similarly as for the CC model.

In the following discussion, we will classify summary data into two distinct types, the regular and irregular summary data. Regular summary data has its corresponding ***g***(***X***; μ*) to be not constantly **0** over ***X***. Irregular summary data is the one with ***g***(***X***; μ*) ≡ **0** for all ***X***. The validity of the proposed procedure for integrating regular summary data requires some standard regularity conditions, which would not hold if ***g***(***X***; μ*) ≡ **0**. Therefore, a different integration procedure is needed for irregular summary data.

### 2.4 Procedure for integrating regular summary data

Here we extend the generalized integration model (GIM) of [38] to fit a PLR model by integrating individual-level and summary data. We call the new procedure PolyGIM.

The log-likelihood for the internal study is given by (3). The log-likelihood function of the summary data can be represented as $-\frac{N}{2}{\left(\beta -\tilde{\beta }\right)}^{⊤}\Sigma_{0}^{-1}\left(\beta -\tilde{\beta }\right)$. Since we do not know **Σ**_0_, we can replace it with a known matrix **V** (e.g., the identity matrix **I**) as the starting point. By combining the two into a joint (pseudo) log-likelihood function, we can estimate ***μ*** via solving the following optimization problem over (P,μ),

$$\begin{array}{c} \begin{array}{cccc} & \underset{P,\mu }{\text{max}} & & \sum_{i=1}^{n}\;\text{log}\;p_{i}+\sum_{i=1}^{n}\sum_{k=1}^{K}1\left(Y_{i}=k\right)\cdot \text{log}\left\{\Delta_{k}\left(X_{i};\xi \right)\right\}-\frac{N}{2}{\left(\beta -\tilde{\beta }\right)}^{⊤}V^{-1}\left(\beta -\tilde{\beta }\right),\\ & \text{subject}\;\text{to} & & \sum_{i=1}^{n}\;p_{i}=1,\;p_{i}\geq 0,\;i=1,\ldots ,n,\\ & & & \sum_{i=1}^{n}\;p_{i}\left\{\Delta_{k}\left(X_{i};\xi \right)-1\right\}=0,\;k=1,\ldots ,K,\\ & & & \sum_{i=1}^{n}\;p_{i}g\left(X_{i};\mu \right)=0. \end{array} \end{array}$$ (10)

The last constraint equation in (10) is from (8) with a specific ***g***.

We employ the Lagrange multiplier approach to solve (10). The Lagrange function can be written as,

$$\begin{array}{cc} L(P,\mu ,\kappa ,\lambda ,\nu )= & \sum_{i=1}^{n}\;\text{log}\;p_{i}+\sum_{i=1}^{n}\sum_{k=1}^{K}1\left(Y_{i}=k\right)\cdot \text{log}\left\{\Delta_{k}\left(X_{i};\xi \right)\right\}-\frac{N}{2}{\left(\beta -\tilde{\beta }\right)}^{⊤}V^{-1}\left(\beta -\tilde{\beta }\right)\\ & -n\kappa (\sum_{i=1}^{n}\;p_{i}-1)-n\sum_{k=1}^{K}\;\lambda_{k}(\sum_{i=1}^{n}p_{i}\left\{\Delta_{k}\left(X_{i};\xi \right)-1\right\})-n\sum_{i=1}^{n}p_{i}\nu^{⊤}g\left(X_{i};\mu \right), \end{array}$$

where *κ*, **λ** and ***ν*** are the Lagrange multipliers. It can be seen that *κ* = 1 and

$$p_{i}=\frac{1}{n}\frac{1}{1+\sum_{k=1}^{K}\lambda_{k}\left\{\Delta_{k}\left(X_{i};\xi \right)-1\right\}+\nu^{⊤}g\left(X_{i};\mu \right)},\;i=1,\ldots ,n.$$

Let ***η*** = (**λ**^⊤^, ***ν***^⊤^, ***μ***^⊤^)^⊤^ be the vector of all variables. Therefore, the profiled log-likelihood function can be written as

$$\begin{array}{c} \begin{array}{cc} \ell_{V}\left(\eta \right)= & -\sum_{i=1}^{n}\text{log}\{1+\sum_{k=1}^{K}\lambda_{k}\left(\Delta_{k}\left(X_{i};\xi \right)-1\right)+\nu^{⊤}g\left(X_{i};\mu \right)\}\\ & +\sum_{i=1}^{n}\sum_{k=1}^{K}1\left(Y_{i}=k\right)\cdot \text{log}\left\{\Delta_{k}\left(X_{i};\xi \right)\right\}-\frac{N}{2}{\left(\beta -\tilde{\beta }\right)}^{⊤}V^{-1}\left(\beta -\tilde{\beta }\right). \end{array} \end{array}$$ (11)

Hence, solving the original problem (10) can be translated into finding the solution of a set of score equations (shown in S1 Appendix). Then we can apply the Newton-Raphson algorithm to to find the solution ${\hat{\eta }}_{V}={\left({\hat{\lambda }}_{V}^{⊤},{\hat{\nu }}_{V}^{⊤},{\hat{\mu }}_{V}^{⊤}\right)}^{⊤}$. In S1 Appendix, we show in Lemma 1 that under some regularity conditions ${\hat{\eta }}_{V}$ is a consistent estimate of η* = (λ*^⊤^, ν*^⊤^, μ*^⊤^)^⊤^, with $\lambda_{k}^{*}=\frac{\rho_{k}}{1+\sum_{i=1}^{K}\rho_{i}}\;\left(k=1,\ldots ,K\right)$ and $\nu^{*}=0$. In particular, regularity condition C4 requires ***g***(***X***; μ*) to be not constantly **0**. More intuitively, if ***g***(***X***; μ*) ≡ **0**, the Lagrange multiplier ***ν*** in (11) is not identifiable. Consequently, the procedure based on (11) is only applicable to summary data that satisfy those regular conditions. We refer to such summary data as regular summary data, while summary data not meeting those conditions are called irregular summary data. Summary data derived from a GC model in general is regular. It is also regular if it is derived from a CC model that does not consider the same set of covariates as the one by the underlying PLR model, as in this setting ***g***(***X***; ***μ***) can not be constantly **0** for any ***μ***.

Based on the estimate ${\hat{\eta }}_{V}$, we can obtain the estimated empirical distribution of *P*(***X***|*Y* = 0) as

$$\begin{array}{c} {\hat{p}}_{i}=\frac{1}{n}\frac{1}{1+\sum_{k=1}^{K}{\hat{\lambda }}_{V,k}\left\{\Delta_{k}\left(X_{i};{\hat{\xi }}_{V}\right)-1\right\}+{\hat{\nu }}_{V}^{⊤}g\left(X_{i};{\hat{\mu }}_{V}\right)},\;i=1,\ldots ,n. \end{array}$$ (12)

Furthermore, we can estimate **A** and **B** in (9) by calculating the expectation over *P*(***X***|*Y* = 0) with ${\hat{p}}_{i}$. An example is shown in S1 Appendix. Therefore, $\text{Cov}(\tilde{\beta })$ can be estimated as $\frac{1}{N}{\hat{\Sigma }}_{0}$, with ${\hat{\Sigma }}_{0}$ given by

$$\begin{array}{c} {\hat{\Sigma }}_{0}={\left({\hat{A}}^{-1}\hat{B}{\hat{A}}^{-1}\right)}_{\beta \beta } \end{array}$$ (13)

which is the submatrix of ${\hat{A}}^{-1}\hat{B}{\hat{A}}^{-1}$ corresponding to ***β***.

Here is the summary of theoretic properties of the estimate.

**Proposition 1** *Under the regularity conditions given in* S1 Appendix, *assuming that* *N*/*n* → *γ*, *N*_1_/*N*_0_ → *ρ* *and* *n*_*k*_/*n* → *ρ*_*k*_ *for* *k* = 1, …, *K* *as* *n* → ∞. *We have* $\sqrt{n}\left({\hat{\mu }}_{V}-\mu^{*}\right)$ *is asymptotically normal, and its asymptotic variance-covariance matrix attains its minimum (in term of positive semidefinite) at* **V** = **Σ**_0_. *Furthermore*, ${\hat{\xi }}_{\Sigma_{0}}$ *is asymptotically more efficient than the internal data based MLE* ${\hat{\xi }}_{mle}$.

Proofs are given in S1 Appendix. The optimality of estimate ${\hat{\mu }}_{\Sigma_{0}}$ still holds when we replace **Σ**_0_ with its consistent estimate ${\hat{\Sigma }}_{0}$. We propose the following iterative Algorithm 1 to obtain this optimal estimate.

**Algorithm 1** Algorithm for PolyGIM

1: Based on the internal study, we fit the PLR model to obtain a consistent estimate of ***ξ***, and fit the working model to obtain a consistent estimate of (***α***, ***β***). We denote these estimates as ${\hat{\eta }}_{0}=\left({\hat{\lambda }}_{0},{\hat{\nu }}_{0},{\hat{\xi }}_{0},{\hat{\alpha }}_{0},{\hat{\beta }}_{0}\right)$, where ${\hat{\lambda }}_{k,0}=\frac{\rho_{k}}{1+\sum_{i=1}^{K}\rho_{i}}\;\left(k=1,\ldots ,K\right)$ and ${\hat{\nu }}_{0}=0$. Then an initial estimate of ${\hat{\Sigma }}_{0}$ is computed according to (13) at ${\hat{\eta }}_{0}$.

2: Resolve the score equation with $V={\hat{\Sigma }}_{0}$. Let the estimates be ${\hat{\eta }}_{{\hat{\Sigma }}_{0}}$.

3: Estimate the empirical probability via (12).

4: Update ${\hat{\Sigma }}_{0}$ via (13).

5: Repeat Steps 2 to 4 until ${\hat{\eta }}_{{\hat{\Sigma }}_{0}}$ is converged.

We use the following strategy to choose the initial point in Step 1. We obtain ${\hat{\xi }}_{0}$ by fitting the PLR model with the internal data. Since we formulate the PLR model with the empirical likelihood representation (4), we adjust estimates of intercept terms from the standard R package for the PLR model by subtracting log(*ρ*_*i*_), *i* = 1, …, *K*. To obtain the initial estimate of (***α***, ***β***), we first adjust for the sample size difference between the internal and external studies by assigning each subject from the internal study an appropriate weight. Then we fit a weighted logistic regression model to obtain the initial estimate of (***α***, ***β***). More specifically, suppose that the external study fits a GC model based on *N*_0_ controls and *N*_1_ grouped cases, with *r*_*i*_ proportion of them having disease subtype *i*(*i* = 1, …, *K*). We assign *N*_0_/*n*_0_ as the weight for each control and (*N*_1_*r*_*i*_)/*n*_*i*_ as the weight for each subtype *i* case in the internal study. We can obtain $\left({\hat{\alpha }}_{0},{\hat{\beta }}_{0}\right)$ by fitting a weighted logistic regression model with those weights. Again, we need to adjust the estimate of the intercept term due to the use of the empirical likelihood representation.

### 2.5 Integrating irregular summary data

When summary data is derived from a working CC model that is consistent with the underlying risk model, it may become irregular with ***g***(***X***; ***μ***) ≡ **0**. More specifically, if *M*_*k*_(⋅) = ***X***^⊤^***θ***_*k*_ in the underlying risk model (2) and *m*(⋅) = ***X***^⊤^***β*** in the working CC model (5) with two subtypes *c*_0_ and *c*_1_, based on the definitions of {*φ*_0_, *φ*_1_, Δ_*k*_, *δ*} in Section 2.2 and 2.3, it can be shown that

$$\begin{array}{cc} g(X;\mu ) & =\varphi_{0}\left(X;\alpha_{0},\beta \right)\Delta_{c_{0}}\left(X;\xi \right)+\rho \varphi_{1}\left(X;\alpha_{0},\beta \right)\Delta_{c_{1}}\left(X;\xi \right)\\ & =\rho \frac{\text{exp}\left\{\tau_{c_{1}}+X^{⊤}\theta_{c_{1}}\right\}-\text{exp}\left\{\tau_{c_{0}}+\alpha_{0}+X^{⊤}\left(\theta_{c_{0}}+\beta \right)\right\}}{1+\rho \delta (X;\alpha_{0},\beta )}\frac{\partial \;\text{log}\;\delta (X;\alpha_{0},\beta )}{\partial (\alpha_{0},\beta )}. \end{array}$$

So we have ***g***(***X***; ***μ*** ≡ **0** if we let

$$\begin{array}{c} \alpha_{0}=\tau_{c_{1}}-\tau_{c_{0}}\;\text{and}\;\beta =\theta_{c_{1}}-\theta_{c_{0}}. \end{array}$$ (14)

Notice that the true value of ***μ*** satisfies (14) due to the consistency between the working model and the underlying risk model. Under these constraints (14), we can eliminate $\sum_{i=1}^{n}p_{i}g\left(X_{i};\mu \right)=0$ from (10) and use the Lagrange multiplier approach to solve a modified version of (10). The resultant profile log-likelihood function can be written as

$$\begin{array}{c} \begin{array}{cc} \ell_{V}^{\prime }\left(\xi \right)= & -\sum_{i=1}^{n}\text{log}\{1+\sum_{k=1}^{K}\rho_{k}\Delta_{k}\left(X_{i};\xi \right)\}+\sum_{i=1}^{n}\sum_{k=1}^{K}1\left(Y_{i}=k\right)\cdot \text{log}\left\{\Delta_{k}\left(X_{i};\xi \right)\right\}\\ & -\frac{N}{2}{\left(\theta_{c_{1}}-\theta_{c_{0}}-\tilde{\beta }\right)}^{⊤}V^{-1}\left(\theta_{c_{1}}-\theta_{c_{0}}-\tilde{\beta }\right). \end{array} \end{array}$$ (15)

We can obtain the estimate ${\hat{\xi }}_{V}={\left({\hat{\tau }}_{V}^{⊤},{\hat{\theta }}_{V}^{⊤}\right)}^{⊤}$ based on (15). Under regularity conditions given in S1 Appendix, we can show that ${\hat{\xi }}_{V}$ is consistent for any given positive definite **V**.

The asymptotic distribution of ${\hat{\xi }}_{V}$ and the optimal choice of **V** are summarized by the following result, with proofs given in S1 Appendix.

**Proposition 2** *Under model* (15) *and regularity conditions given in* S1 Appendix, *we have* $\sqrt{n}\left({\hat{\xi }}_{V}-\xi^{*}\right)$ *is asymptotically normal, and its asymptotic variance-covariance matrix attains its minimum at* **V** = **Σ**_0_. *In particular, the asymptotic variance-covariance matrix of* $\sqrt{n}{\hat{\xi }}_{\Sigma_{0}}$ *has the following form*,

$$J_{\Sigma_{0}}^{{}^{\prime }-1}-[\begin{array}{cc} \Gamma^{\prime } & 0\\ 0 & 0 \end{array}],$$

*where* $J_{\Sigma_{0}}^{\prime }$ *is defined in* S1 Appendix, *and* $\Gamma^{\prime }=\text{diag}\left(\frac{1}{\lambda_{1}^{*}},\ldots ,\frac{1}{\lambda_{K}^{*}}\right)+\frac{1}{1-\sum_{k}\lambda_{k}^{*}}11^{⊤}$ *with* $\lambda_{k}^{*}=\frac{\rho_{k}}{1+\sum_{i=1}^{K}\rho_{i}}\;\left(k=1,\ldots ,K\right)$ *and* **1** *being a vector of 1’s*.

Similar to the regular PolyGIM procedure, we can obtain ${\hat{\xi }}_{{\hat{\Sigma }}_{0}}$ by an iterative algorithm. Even though we use different procedures for integrating regular and irregular summary data, in the following discussion we still call them the PolyGIM procedure when there is no confusion.

We can also use a restricted MLE (RMLE) approach to incorporate this irregular summary data. Let ${\hat{\xi }}_{rmle}$ be the solution of the following constraint optimization problem,

$$\begin{array}{c} \begin{array}{cccc} & \underset{\xi }{\text{max}} & & -\sum_{i=1}^{n}\;\text{log}\;\{1+\sum_{k=1}^{K}\rho_{k}\Delta_{k}\left(X_{i};\xi \right)\}+\sum_{i=1}^{n}\sum_{k=1}^{K}1\left(Y_{i}=k\right)\cdot \text{log}\left\{\Delta_{k}\left(X_{i};\xi \right)\right\},\\ & \text{subject}\;\text{to} & & \theta_{c_{1}}-\theta_{c_{0}}-\tilde{\beta }=0. \end{array} \end{array}$$ (16)

This setup is different from the standard RMLE, as $\tilde{\beta }$ in the constraint equation has variability. We need to account for this uncertainty when estimating the variance-covariance matrix of ${\hat{\xi }}_{rmle}$. In S1 Appendix, we prove the following result that shows the PolyGIM estimate is more efficient than other considered estimates.

**Proposition 3** *The estimate* ${\hat{\xi }}_{\Sigma_{0}}$ *based on* (15) *is asymptotically more efficient than both the internal data based MLE* ${\hat{\xi }}_{mle}$ *and the RMLE* ${\hat{\xi }}_{rmle}$.

### 2.6 Test for disease subtype heterogeneity

As mentioned in the Introduction, researchers are often interested in testing whether a risk factor *X*_*j*_ has the same effect on different disease subtypes, with the null hypothesis being *H*_0_ : *θ*_*j*1_ = *θ*_*j*2_ = … = *θ*_*jK*_. We can use PolyGIM to combine data from multiple sources for a more efficient test.

By Proposition 1, ${\hat{\theta }}_{j\cdot }={\left({\hat{\theta }}_{j1},{\hat{\theta }}_{j2},\ldots ,{\hat{\theta }}_{jK}\right)}^{⊤}$, the estimated coefficients corresponding to *X*_*j*_, has a multivariate normal distribution $N(\theta_{j},\hat{W})$, where $\hat{W}$ is extracted from the estimated variance-covariance matrix of ${\hat{\eta }}_{\hat{\Sigma }}$. So its log-likelihood function can be written as

$$Q\left(\theta_{j\cdot }\right)=-\frac{1}{2}{\left({\hat{\theta }}_{j\cdot }-\theta_{j\cdot }\right)}^{⊤}{\hat{W}}^{-1}\left({\hat{\theta }}_{j\cdot }-\theta_{j\cdot }\right)-\frac{K}{2}\text{ln}\left(2\pi \right)-\frac{1}{2}\text{ln}\;\text{det}\left(\hat{W}\right).$$

Under the null, ${\hat{\theta }}_{j\cdot }$ follows $N(a1,\hat{W})$. The MLE of *a* under the null can be derived as $\hat{a}=\left(1^{⊤}{\hat{W}}^{-1}{\hat{\theta }}_{j\cdot }\right)/\left(1^{⊤}{\hat{W}}^{-1}1\right)$. Thus, we can construct the following likelihood ratio test,

$$\Lambda \;≜\;-2\left[Q\left(\hat{a}1\right)-Q\left({\hat{\theta }}_{j\cdot }\right)\right]={\hat{\theta }}_{j\cdot }^{⊤}{\hat{W}}^{-1}{\hat{\theta }}_{j\cdot }-\frac{{\left(1^{⊤}{\hat{W}}^{-1}{\hat{\theta }}_{j\cdot }\right)}^{2}}{1^{⊤}{\hat{W}}^{-1}1}.$$

Under the null, this test follows a chi-square distribution with *K* − 1 degrees of freedom.

### 2.7 Summary data from multiple models

So far, we have considered summary data from a single working model based on one external study. Here we show how to incorporate summary data from multiple working models, some of which can be fitted with overlapping samples. For example, in the NHL example, we have summary data from seven independent external studies. From one study consisting of cases of three NHL subtypes, we have three summary statistics on each SNP, which are estimated from three GC models sharing a common set of controls. For notation simplicity, we focus on regular summary data in the following discussion.

First, we provide some theoretical insights on the PolyGIM procedure with multiple summary data. Suppose that we are given the summary data ${\tilde{\beta }}_{i}\;\left(i=1,2\right)$, from two external models. Let ***α***_*i*_ (*i* = 1, 2) be the corresponding nuisance parameters. Similar to Section 2.3, we can establish the following asymptotics

$$\sqrt{N}[\begin{array}{c} {\tilde{\beta }}_{1}-\beta_{1}^{*}\\ {\tilde{\beta }}_{2}-\beta_{2}^{*} \end{array}]\overset{\;d\;}{\to }N(0,\Sigma =[\begin{array}{cc} \Sigma_{11} & \Sigma_{12}\\ \Sigma_{21} & \Sigma_{22} \end{array}]),$$

where $\beta_{i}^{*}\;\left(i=1,2\right)$ are the true values. The specific form of **Σ** depends on the two external models and whether there are overlapped samples used for fitting them. For example, if the two external models are fitted with data from two different studies, we have **Σ**_12_ = **0**. If overlapped samples are used for fitting the two models, **Σ**_12_ ≠ **0**. Later we will provide more details on how to estimate **Σ**.

Let ${\hat{\xi }}_{\Sigma_{11}}$ be the optimal PolyGIM estimate of ***ξ*** using summary data ${\tilde{\beta }}_{1}$, ${\hat{\xi }}_{\Sigma }$ be the optimal estimate using summary data $\left\{{\tilde{\beta }}_{1},{\tilde{\beta }}_{2}\right\}$. We can show theoretically that it is always beneficial by using more summary data (Proposition 4), see S1 Appendix.

More technical details on integrating summary data from multiple studies are presented in S1 Appendix.

## 3 Results

### 3.1 Simulation studies with summary data from one external study

To verify the theoretic properties of the proposed PolyGIM method, we first considered a simple scenario when summary data comes from one external study. Assume that ***X*** = (*X*_1_, *X*_2_) was a vector of two binary biomarkers and that the outcome *Y* had three classes, with 0 for the control group and 1/2 for the two disease subtypes. In the study population, the two biomarkers were correlated, with the joint probability of (*X*_1_, *X*_2_) = (0, 0), (0, 1), (1, 0), and (1, 1) specified as 0.28, 0.12, 0.18 and 0.42, respectively. The true disease risk model was chosen as

$$\text{log}\{\frac{P(Y=k|X)}{P(Y=0|X)}\}=\omega_{k}+\theta_{k1}X_{1}+\theta_{k2}X_{2},\;k=1,2.$$

We let (*ω*_1_, *ω*_2_) = (−0.2, 0.1) and (*θ*_11_, *θ*_12_, *θ*_21_, *θ*_22_) = (1.5, −1.0, −0.5, 1.2). From this population, case-control studies were retrospectively generated conditioning on the outcome *Y*, with the internal study consisting of 2000 controls, 500 subtype 1 cases and 500 subtype 2 cases, and the external study consisting of 1000 controls, 600 subtype 1 cases and 400 subtype 2 cases.

We considered two classes of summary data, one from the CC model and the other from the GC model. The binary outcome *D* used in a CC model was defined as *D* = 0 for subtype 1 and *D* = 1 for subtype 2. Summary data from two types of CC working models (CC1 and CC2) were studied. The CC1 working model only included covariate *X*_1_ and thus was inconsistent with the underlying risk model. It generated regular summary data ${\tilde{\beta }}_{1}$, which was the estimated coefficient of *X*_1_. The CC2 working model was consistent with the underlying risk model and included covariates (*X*_1_, *X*_2_). It generated irregular summary data $\left({\tilde{\beta }}_{1},{\tilde{\beta }}_{2}\right)$, which were estimated coefficients of *X*_1_ and *X*_2_.

The GC model merged the two disease subtypes into one group and compared it with the control group. Two GC working models (GC1 and GC2) were used to generate regular summary data. GC1 model included only *X*_1_ as a covariate and generated summary data ${\tilde{\beta }}_{1}$. GC2 model had *X*_1_ and *X*_2_ as covariates and generated summary data $\left({\tilde{\beta }}_{1},{\tilde{\beta }}_{2}\right)$.

For each simulated dataset (including an internal study and summary data), we applied three versions of PolyGIM, one based on the most optimal estimate ${\hat{\theta }}_{{\hat{\Sigma }}_{0}}$ (called GIM_*opt*_), the others based on the estimate with two options of **V**, one called GIM_**I**_ with **V** = **I**, and the other call ${\text{GIM}}_{{}_{V_{\sigma }}}$ with **V** = **V**_*σ*_, a diagonal matrix that has each diagonal element being $\sigma_{i}^{2}/N$, with $\sigma_{i}^{2}$ being the variance of the *i*-th summary statistic from the summary data $\tilde{\beta }$. We applied different types of PolyGIM procedures depending on whether the summary data was regular or irregular. As a comparison, we also analyzed the internal study using the standard PLR model (called MLE_*int*_). We simulated 2000 datasets under each scenario to evaluate the performance of all considered methods. Tables 1 and 2 summarized simulation results in situations when summary data is generated from CC and GC models, respectively.

**Table 1:** Simulation results in situations when summary data is derived from one external study based on case-case (CC) comparison models.

		MLE_*int*_	CC1: Given ${\tilde{\beta }}_{1}$			CC2: Given $\left({\tilde{\beta }}_{1},{\tilde{\beta }}_{2}\right)$		
			GIM_**I**_	${\text{GIM}}_{{}_{V_{\sigma }}}$	GIM_*opt*_	GIM_**I**_	${\text{GIM}}_{{}_{V_{\sigma }}}$	GIM_*opt*_
*θ*_11_	Bias	0.27	0.40	0.27	0.29	0.87	0.24	0.45
	SE-Emp	12.53	12.45	11.33	11.34	13.85	11.54	11.30
	SE-Est	12.58	12.38	11.34	11.34	13.68	11.53	11.30
	CP	95.20	94.85	95.65	95.75	94.55	95.05	95.15
*θ*_21_	Bias	−0.24	−0.36	−0.30	−0.32	−0.83	−0.24	−0.46
	SE-Emp	12.13	11.77	11.25	11.25	13.10	11.10	10.98
	SE-Est	11.95	11.83	11.20	11.20	13.17	11.10	10.95
	CP	94.90	94.95	94.70	94.70	94.60	94.25	94.50
*θ*_12_	Bias	−0.22	−0.22	−0.22	−0.22	−0.62	−0.17	−0.35
	SE-Emp	11.53	11.53	11.53	11.53	12.87	10.86	10.66
	SE-Est	11.65	11.65	11.65	11.65	12.62	10.86	10.70
	CP	95.65	95.65	95.65	95.65	94.65	95.25	95.55
*θ*_22_	Bias	0.28	0.28	0.28	0.28	0.77	0.19	0.39
	SE-Emp	12.80	12.80	12.80	12.80	14.02	11.62	11.50
	SE-Est	12.55	12.55	12.55	12.55	13.81	11.50	11.29
	CP	94.25	94.25	94.25	94.25	94.80	95.00	94.35

All numbers are multiplied by 100. SE-Emp: empirical standard error; SE-Est: mean of estimated standard error; CP: coverage probability of a 95% confidence interval; CC1/CC2: case-case comparison model with covariates *X*_1_/{*X*_1_, *X*_2_}; MLE_*int*_: MLE based on the internal study; GIM_**I**_: PolyGIM with **V** = **I**; ${\text{GIM}}_{{}_{V_{\sigma }}}$: PolyGIM with **V** = **V**_*σ*_; GIM_*opt*_: the optimal PolyGIM.

**Table 2:** Simulation results in situations when summary data is derived from one external study based on grouped case-control (GC) models.

		MLE_*int*_	GC1: Given ${\tilde{\beta }}_{1}$			GC2: Given $\left({\tilde{\beta }}_{1},{\tilde{\beta }}_{2}\right)$		
			GIM_**I**_	${\text{GIM}}_{{}_{V_{\sigma }}}$	GIM_*opt*_	GIM_**I**_	${\text{GIM}}_{{}_{V_{\sigma }}}$	GIM_*opt*_
*θ*_11_	Bias	0.27	0.47	0.34	0.36	0.56	0.38	0.41
	SE-Emp	12.53	13.14	11.03	11.03	13.37	10.72	10.65
	SE-Est	12.58	13.10	11.03	11.03	13.57	10.85	10.71
	CP	95.20	95.65	95.30	95.30	95.85	95.25	95.25
*θ*_21_	Bias	−0.24	0.04	−0.16	−0.15	0.18	−0.12	−0.11
	SE-Emp	12.13	12.67	11.35	11.35	12.84	11.08	11.05
	SE-Est	11.95	12.27	11.04	11.04	12.64	10.86	10.78
	CP	94.90	93.35	93.90	93.90	93.95	94.35	94.35
*θ*_12_	Bias	−0.22	−0.22	−0.22	−0.22	−0.32	−0.29	−0.28
	SE-Emp	11.53	11.53	11.53	11.53	12.53	9.77	9.66
	SE-Est	11.65	11.65	11.65	11.65	12.78	9.94	9.80
	CP	95.65	95.65	95.65	95.65	95.35	95.60	95.65
*θ*_22_	Bias	0.28	0.28	0.28	0.28	0.01	0.19	0.21
	SE-Emp	12.80	12.80	12.80	12.80	13.37	11.79	11.73
	SE-Est	12.55	12.55	12.55	12.55	13.26	11.54	11.47
	CP	94.25	94.25	94.25	94.25	94.95	94.90	94.85

All numbers are multiplied by 100. GC1/GC2: grouped case-control model with covariates *X*_1_/{*X*_1_, *X*_2_}.

Table 1 for the CC models shows that all considered methods have expected performances, with unbiased estimates and their estimated standard errors matching well with corresponding empirical standard errors. When using irregular summary data $\left({\tilde{\beta }}_{1},{\tilde{\beta }}_{2}\right)$, we can see that GIM_*opt*_ provides more efficient estimates of {*θ*_*k*1_, *θ*_*k*2_, *k* = 1, 2} compared to GIM_**I**_, ${\text{GIM}}_{{}_{V_{\sigma }}}$ and MLE_*int*_. These findings align with the conclusions of Propositions 2 and 3. If only ${\tilde{\beta }}_{1}$ is used as summary data, GIM_*opt*_ and ${\text{GIM}}_{{}_{V_{\sigma }}}$ are more effective for estimating coefficients of *X*_1_, whereas all methods have similar efficiency levels for estimating coefficients of *X*_2_. Notably, results from Table 1 reveal that ${\text{GIM}}_{{}_{V_{\sigma }}}$ has a similar level of efficiency as GIM_*opt*_ when integrating only one summary statistic, but it becomes less efficient when integrating two correlated summary statistics. This efficiency loss arises because ${\text{GIM}}_{{}_{V_{\sigma }}}$ selects the matrix **V** = **V**_*σ*_ in (10), assuming all summary statistics to be independent. This chosen **V**_*σ*_ is suboptimal when summary statistics are correlated, since the optimal matrix should be a consistent estimate of the variance-covariance matrix for these statistics. However, when the summary statistics are indeed independent, **V**_*σ*_ can serve as a satisfactory practical approximation for the optimal matrix. Although they differ theoretically in their diagonal terms when external models are mis-specified (e.g., the CC1 model), this discrepancy is more theoretical than practical in our context, as evident from S1 Table. From Table 2 and S2 Table. for the GC models, we can reach similar conclusions as those from Table 1 and S1 Table.

### 3.2 Simulation studies with summary data from multiple external studies

We conducted additional simulation studies under a more complex setting to mimic the NHL study. We considered 21 SNPs *X* = (*X*_1_, …, *X*_21_), and the outcome *Y* had five classes, with *Y* = 0 representing controls and *Y* = 1 to 4 representing four different disease subtypes. We selected the 21 SNPs identical to those used in the NHL example, with the exception that we assumed they were independent to simplify the simulation procedure. The characteristics of these SNPs are summarized in S3 Table. The true underlying risk model was defined as

$$\text{log}\{\frac{P(Y=k|X)}{P(Y=0|X)}\}=\omega_{k}+\theta_{k}S\left(X\right),\;k=1,\ldots ,4,$$

with the PRS $S\left(X\right)=\sum_{i=1}^{21}w_{i}X_{i}$. We considered the following two set of parameters for the true model:

**Null PRS Model:** (*ω*_1_, *ω*_2_, *ω*_3_, *ω*_4_) = (−3.9, −4.1, −3.6, −3.8) and (*θ*_1_, *θ*_2_, *θ*_3_, *θ*_4_) = (0, 0, 0, 0).
**Alternative PRS Model:** *ω*_*i*_ = −3.8, *i* = 1, …, 4, and (*θ*_1_, *θ*_2_, *θ*_3_, *θ*_4_) = (0.019, 0.092, −0.12, 0.047).

Both models were designed with intercept terms to ensure the rarity of each disease subtype in the study population (each with a prevalence of less than 2%). For Alternative PRS Model, the effects of the PRS were chosen to match those observed in the NCI study of the NHL example.

In practice the weights (*w*_*i*_) used in PRS are estimated with uncertainty from other studies, leading to a PRS with measurement error. We aim to evaluate the performance of procedures under consideration while accounting for the measurement error. To accomplish this, we generated estimates (${\tilde{w}}_{i}$) of the true weights (*w*_*i*_) for each simulated dataset, assuming that the true weights *w*_*i*_ were identical to those used in the real NHL example (S3 Table). We randomly generated ${\tilde{w}}_{i}$ from a normal distribution $N(w_{i},c\cdot {\text{se}}_{i}^{2})$, where se_*i*_ was the standard error reported in the published GWAS from which the SNP was genome-wide significantly detected (see S3 Table). We used ${\tilde{w}}_{i}$ to calculate the PRS with measurement error, denoted as $\tilde{S}\left(X\right)=\sum_{i=1}^{21}{\tilde{w}}_{i}X_{i}$, and varied the level of measurement error by choosing the scaling factor *c* from {0, 1, 16}. In the analysis of each simulated dataset, we used $\tilde{S}\left(X\right)$ instead of *S*(*X*) to reflect the measurement error.

First, we considered performance of MLE_*int*_ and GIM_*opt*_ under the null model. We used summary data from External Studies 1–5 listed in Table 3. All these five external studies, as well as the internal study, were generated from the same source population (the internal study population). S3 Table provides the allele frequency of the effect allele “1” for each of the 21 SNPs in the study population. For each external study, we used a logistic regression model to estimate the marginal effect of each SNP (i.e., the regression coefficient) on the risk of a specific disease subtype. The summary data consisted of these coefficient estimates for all considered 21 SNPs. We simulated 2000 datasets under Null PRS model and analyzed each dataset with MLE_*int*_ and GIM_*opt*_. To assess the impact of measurement error, we employed three sets of PRS in the analysis of each simulated dataset, including the PRS without measurement error (i.e., ${\tilde{w}}_{i}=w_{i}$), the PRS with measurement error at the same level as the real NHL study (i.e., ${\tilde{w}}_{i}∼N\left(w_{i},{\text{se}}_{i}^{2}\right)$), and the PRS with elevated measurement error (i.e., ${\tilde{w}}_{i}∼N\left(w_{i},16{\text{se}}_{i}^{2}\right)$). The simulation results, presented in Table 4, indicate that the level of uncertainty in PRS does not have an impact on the statistical properties of MLE_*int*_ and GIM_*opt*_. This is expected since likelihood models for both procedures remain valid under the null model (i.e., ***θ*** = **0**), even when using PRS with high levels of measurement error. Consequently, the estimate of the effect of the PRS remains consistent. Additionally, we evaluated the type I errors of GIM_*opt*_ at various significance levels, as shown in S4 Table, and generated the Q-Q plots of estimated *Z*-scores for the PRS effect on different subtypes in S1 Fig. These results demonstrate that GIM_*opt*_ has well calibrated type I errors and *P*-values.

**Table 3:** Simulation results in situations when summary data is derived from one external study based on grouped case-control (GC) models.

GWAS		Subtype 1 (CLL)	Subtype 2 (DLBCL)	Subtype 3 (FL)	Subtype 4 (MZL)	Controls
Internal study	NCI(US)	2179	2661	2142	825	6221
External study 1	USCF2(US)	213	254	210	0	748
External study 2	GEC(US)	387	0	0	0	294
External study 3	UTAH(US)	321	0	0	0	405
External study 4	MAYO(US)	0	393	0	0	172
External study 5	UCSF1(US)	0	0	119	0	349
External study 6	GELA(EU)	0	549	0	0	525
External study 7	SCALE(EU)	0	0	376	0	791

**Table 4:** Simulation results on the impact of measurement error under the null PRS model.

		None		Low		High	
		MLE	GIM_*opt*_	MLE	GIM_*opt*_	MLE	GIM_*opt*_
*θ*_1_	Bias	0.05	0.02	−0.12	−0.16	−0.10	−0.14
	SE-Emp	3.23	2.81	3.16	2.77	2.81	2.51
	SE-Est	3.20	2.78	3.17	2.76	2.85	2.47
	CP	95.00	94.60	95.25	95.40	95.05	95.40
*θ*_2_	Bias	0.05	0.02	−0.04	−0.06	−0.04	−0.06
	SE-Emp	3.00	2.78	2.95	2.76	2.72	2.55
	SE-Est	2.98	2.74	2.95	2.71	2.65	2.44
	CP	95.05	95.00	95.15	94.40	94.75	94.50
*θ*_3_	Bias	0.04	−0.01	−0.16	−0.22	−0.13	−0.18
	SE-Emp	3.27	3.01	3.17	2.94	2.85	2.65
	SE-Est	3.22	2.97	3.19	2.95	2.87	2.65
	CP	94.55	94.50	94.85	94.95	95.05	95.40
*θ*_4_	Bias	0.04	0.02	−0.11	−0.13	−0.14	−0.16
	SE-Emp	4.68	4.65	4.64	4.62	4.19	4.17
	SE-Est	4.76	4.73	4.72	4.69	4.24	4.21
	CP	95.95	95.90	95.25	95.30	95.00	95.05

Measurement errors are considered at three different levels: none (i.e., ${\tilde{w}}_{i}=w_{i}$), low (i.e., ${\tilde{w}}_{i}∼N\left(w_{i},{\text{se}}_{i}^{2}\right)$), and high (i.e., ${\tilde{w}}_{i}∼N\left(w_{i},16{\text{se}}_{i}^{2}\right)$). Summary data are derived from five external studies with their sample sizes giving in Table 3. All numbers are multiplied by 100.

Next, we assessed the performance of the considered procedures under Alternative PRS Model with non-zero PRS effects, and we summarized the simulation results in Table 5. Here are some notable observations. Firstly, when the PRS has either no or relatively low measurement error, both MLE_*int*_ and GIM_*opt*_ exhibit desirable statistical properties in terms of consistency, the accuracy of standard error estimation, and 95% confidence interval coverage probability. In these cases, GIM_*opt*_ is more efficient than MLE_*int*_. Secondly, when the PRS has increased measurement error with ${\tilde{w}}_{i}∼N\left(w_{i},16{\text{se}}_{i}^{2}\right)$, estimates obtained using MLE_*int*_ and GIM_*opt*_ become inconsistent, with a noticeable increase in bias.

**Table 5:** Simulation results on the impact of measurement error under the alternative PRS model.

		None		Low		High	
		MLE	GIM_*opt*_	MLE	GIM_*opt*_	MLE	GIM_*opt*_
*θ*_1_	Bias	0.03	−0.04	0.10	2.73E-03	−0.31	−0.39
	SE-Emp	3.21	2.79	3.11	2.75	2.85	2.52
	SE-Est	3.20	2.78	3.17	2.76	2.85	2.48
	CP	95.30	95.00	96.00	95.25	95.35	94.65
*θ*_2_	Bias	0.10	0.09	−0.15	−0.18	-2.06	-2.09
	SE-Emp	2.99	2.73	2.97	2.76	2.86	2.66
	SE-Est	2.97	2.73	2.95	2.71	2.65	2.43
	CP	94.65	94.75	94.90	94.70	84.50	82.40
*θ*_3_	Bias	0.06	−0.05	0.19	0.11	2.72	2.63
	SE-Emp	3.31	3.04	3.29	3.02	3.13	2.90
	SE-Est	3.24	2.99	3.21	2.96	2.89	2.66
	CP	94.25	94.45	95.15	94.60	80.60	78.55
*θ*_4_	Bias	2.89E-03	−0.03	0.07	0.03	−0.96	−0.99
	SE-Emp	4.70	4.65	4.75	4.72	4.33	4.29
	SE-Est	4.75	4.72	4.72	4.68	4.24	4.21
	CP	95.10	95.15	94.75	94.70	93.35	93.70

Measurement errors are considered at three different levels: none (i.e., ${\tilde{w}}_{i}=w_{i}$), low (i.e., ${\tilde{w}}_{i}∼N\left(w_{i},{\text{se}}_{i}^{2}\right)$), and high (i.e., ${\tilde{w}}_{i}∼N\left(w_{i},16{\text{se}}_{i}^{2}\right)$). Summary data are derived from five external studies with their sample sizes given in Table 3. All numbers are multiplied by 100.

We further evaluated how the sample sizes of external studies affect the efficiency of GIM_*opt*_ by increasing the sample size of the five external studies by 5 and 10 times, with a focus on PRS that have no or relatively low measurement error (i.e., ${\tilde{w}}_{i}∼N\left(w_{i},{\text{se}}_{i}^{2}\right)$). The results, which are summarized in Table 6, demonstrate that the efficiency of GIM_*opt*_ improves as the sample sizes of external studies increase. This same pattern is observed when using an internal study with only 10% of the original sample size (see S5 Table).

**Table 6:** Simulation results on the impact of sample sizes of external studies under the alternative PRS model.

		1 × sample size		5 × sample size		10 × sample size	
		None	Low	None	Low	None	Low
*θ*_1_	Bias	−0.04	2.73E-03	−1.87E-03	0.03	−0.04	−0.07
	SE-Emp	2.79	2.75	1.98	1.92	1.58	1.57
	SE-Est	2.78	2.76	1.98	1.96	1.56	1.54
	CP	95.00	95.25	95.60	95.80	94.90	94.60
*θ*_2_	Bias	0.09	−0.18	0.07	−0.13	0.09	−0.20
	SE-Emp	2.73	2.76	2.22	2.19	1.82	1.79
	SE-Est	2.73	2.71	2.16	2.15	1.79	1.78
	CP	94.75	94.70	94.60	94.65	94.65	95.25
*θ*_3_	Bias	−0.05	0.11	0.02	0.20	0.05	0.10
	SE-Emp	3.04	3.02	2.41	2.44	2.05	2.02
	SE-Est	2.99	2.96	2.38	2.36	1.98	1.97
	CP	94.45	94.60	94.60	94.25	94.00	94.00
*θ*_4_	Bias	−0.03	0.03	−0.03	0.02	−0.03	−0.03
	SE-Emp	4.65	4.72	4.60	4.65	4.57	4.64
	SE-Est	4.72	4.68	4.67	4.63	4.65	4.61
	CP	95.15	94.70	95.55	94.75	95.40	95.00

Summary data are derived from five external studies, with their sample sizes varying from 1, 5 to 10 times the original sizes reported in Table 3. PRS with no or low measurement error is considered. All numbers are multiplied by 100.

Next, we conducted simulations to compare strategies for integrating summary data when there were differences in the joint distribution of ***X*** between the internal and external study populations. Seven external studies were considered, as listed in Table 3. The first five were generated from the same source population as the internal study, while External Studies 6 and 7 were generated from two different external study populations. The allele frequency of allele “1” in External Study 6 was 0.2 lower than that in the internal study population for each of the first 10 SNPs listed in S3 Table, with a lower bound of 0.05. Similarly, in External Study 7, the allele frequency of allele “1” was 0.2 lower than that in the internal study population for each of the last 11 SNPs listed in S3 Table, with a lower bound of 0.05. All other SNPs in these two external studies had the same distribution as in the internal study population. It is worth noting that the discrepancy in the SNP distribution between the two studies is substantially higher than the discrepancy observed in the NHL study. We examined three strategies for integrating summary data from the seven external studies. The first strategy utilized summary data from only the first five external studies. The second strategy employed complete summary data, including summary statistics on the 21 SNPs across all seven studies. The third strategy used summary data from the first five external studies and partial summary data from the remaining two external studies, which included summary data only on SNPs having the same allele frequency as in the internal study population. This excluded summary data on the first 10 SNPs from External Study 6 and the last 11 SNPs from External Study 7. To investigate the impact of varying allele frequencies between the internal and external study populations, we conducted additional simulations. Specifically, we increased the sample sizes of External Studies 6 and 7 by a factor of five to further explore this effect.

Table 7 summarizes the results of our simulation study, from which several observations can be made. The GIM_*opt*_ method, when using additional partial summary data from External Studies 6 and 7, shows desirable statistical properties and is more efficient than using only summary data from the first five external studies. This advantage becomes more pronounced as we increase the sample size of the two additional external studies. In contrast, GIM_*opt*_ using complete summary data could lead to erroneous standard error estimates, especially when the sample size is increased in External Studies 6 and 7. This is an expected outcome, as the PolyGIM procedure is tailored to the scenario where the internal and external studies are conducted in the same source population. Regarding the use of partial summary data, we can show, using arguments similar to those presented in [42], that it is valid to use partial summary data on a subset of SNPs if the following conditions are met. First, the joint distribution of this subset of SNPs in the external study population must be the same as in the internal study population. Second, these SNPs must be independent of the remaining SNPs in both study populations. Third, the disease prevalence must be relatively rare in both populations so that the SNP distribution is similar between the control group and the general population. Our simulation study demonstrated that the use of partial summary data is valid and beneficial when these underlying assumptions are satisfied.

**Table 7:** Simulation results comparing different summary data integration strategies under the alternative PRS model.

		Five external studies	Seven external studies		Seven extended external studies	
			Complete	Partial	Complete	Partial
*θ*_1_	Bias	2.73E-03	−4.08E-03	−3.33E-03	−0.01	−1.34E-03
	SE-Emp	2.75	2.74	2.74	2.74	2.73
	SE-Est	2.76	2.74	2.75	2.70	2.72
	CP	95.25	95.35	95.35	94.70	95.25
*θ*_2_	Bias	−0.18	−0.15	−0.18	−0.22	−0.19
	SE-Emp	2.76	2.68	2.69	2.46	2.54
	SE-Est	2.71	2.55	2.65	2.12	2.47
	CP	94.70	94.05	95.20	90.40	94.70
*θ*_3_	Bias	0.11	0.03	0.07	0.06	0.09
	SE-Emp	3.02	2.88	2.89	2.54	2.49
	SE-Est	2.96	2.77	2.83	2.27	2.43
	CP	94.60	94.15	94.70	92.35	94.45
*θ*_4_	Bias	0.03	0.02	0.02	0.01	0.03
	SE-Emp	4.72	4.71	4.70	4.67	4.68
	SE-Est	4.68	4.67	4.68	4.64	4.66
	CP	94.70	95.10	94.90	94.65	94.95

Three sets of summary data are considered, summary data first five external studies presented in Table 3; summary data from all seven external studies presented in Table 3; and summary data derived from extended seven external studies, with sample sizes for the last two studies increased by five-fold. The sixth and seventh external studies have different allele frequencies compared to the first five. Two strategies for using summary data are assessed: using all summary data from the external studies (complete), and using only summary data from SNPs with the same distribution as the ones in the internal study population (partial). PRS with a low level of measurement error is employed. All numbers are multiplied by 100.

Finally, we carried out supplementary experiments to assess the computational efficiency and memory requirements of the PolyGIM package. In these experiments, we focused on the internal study and the external studies listed in Table 3, and analyzed a PRS with an increasing number of SNPs, ranging from 21 to 105. We considered summary data derived from the first 1, 3, or 5 external studies provided in Table 3. The computational time and memory requirements were recorded over 100 replications and are presented in S6 and S7 Tables. These tables offer a glimpse into the performance of the PolyGIM package under various conditions.

### 3.3 Real data application: The NHL study

NHL is the most common hematological malignancy with many subtypes with distinct molecular and clinical features. It has been hypothesized that various NHL subtypes and other lymphoid malignancies might share some degree of genetic susceptibility. Recently, [39] used eight GWAS within the InterLymph Consortium to study the genetic heritability in four major NHL subtypes, including CLL, DLBCL, FL, and MZL. To explore pleiotropy between NHL subtypes and other lymphoid malignancies (e.g., acute lymphoblastic leukemia and Hodgkin lymphoma), they generated PRS using SNPs that had been established as being associated with each lymphoid malignancy and tested their associations with risk for the four NHL subtypes.

We utilized PolyGIM to analyze the project described by [39], using the PRS for Hodgkin lymphoma (HL) as our primary example. This PRS was derived from 21 HL-associated SNPs that were selected by [39] due to their identification as genome-wide significant SNPs, each with a *P*-value of less than 5 × 10^−8^ from seven previously published GWAS [43–49]. Each SNP was selected as an index SNP to represent a nearby gene, and they were mostly independent of each other, with only two pairs having *R*^2^ > 0.01 (one pair with *R*^2^ = 0.024 and the other with *R*^2^ = 0.038. The PRS was calculated as $S\left(X\right)=\sum_{j=1}^{21}w_{j}X_{j}$, where *X*_*j*_ represents the genotype, and *w*_*j*_ (weight) is the estimated effect by the *j*-th SNP on HL. Further information on the 21 SNPs and their estimated weights used in the PRS calculation can be found in S3 Table.

[39] collected data from eight GWAS with European ancestry, including six US-based studies and two European studies. Table 3 shows the sample sizes of cases with specific NHL subtypes and controls from each study. S2 Fig displays the minor allele frequency (MAF) of each of the 21 SNPs in the control groups of the eight studies. The plot exhibits a consistent distribution of MAF across the studies, with a maximum range of approximately 0.1. This suggests that it is reasonable to assume that all eight studies were conducted on the same population.

For our analysis, we obtained individual-level data from the US-based NCI study, which collected cases from all four NHL subtypes and controls, and treated it as the internal study. We obtained SNP-level summary statistics from the other seven studies (external studies), consisting of each SNP’s marginal effect (i.e., the estimated regression coefficient) on one NHL subtype. We assumed the following PLR model to assess the effect of PRS on the four NHL subtype,

$$\text{log}\{\frac{P(Y=k|X)}{P(Y=0|X)}\}=\omega_{k}+\theta_{k}S\left(X\right),\;k=1,\ldots ,4.$$

We utilized PolyGIM to fit the model in two different ways. The first approach involved integrating summary data solely from the five US-based studies, while the second approach integrated summary data from all seven studies, which included the five US-based studies and two European studies.

Table 8 summarizes the estimates of the effects of PRS on the four subtypes of NHL. The two PolyGIM approaches provide consistent results, indicating that the PRS has a significant effect on DLBCL and FL subtypes. Using summary data from all seven studies yields slightly more significant results compared to using only summary data from the five US-based studies, as the two European studies contribute additional cases on DLBCL and FL subtypes. It is interesting to notice that the PRS is positively associated with the risk of DLBCL but inversely associated with the risk of FL. This observation indicates that SNPs that increase the risk of HL tend to be associated with an increased risk of DLBCL but a reduced risk of FL. On the other hand, the PRS has no significant effect on the risk of CLL or MZL, suggesting that it is less likely to have SNPs with pleiotropic effects on both HL and CLL/MZL. In Table 9, we compared PRS effects on each pair of NHL subtypes. A global test for disease subtype heterogeneity based on the one given in Section 2.6 is significant, with *P*-value = 6.97 × 10^−11^ based on PolyGIM using summary data from all seven external studies. We also present results based on the PLR model fitted with the internal study (the NCI study). As expected, PolyGIM estimates are more efficient than internal study-based MLE since summary data from external studies provide additional helpful information. However, the overall improvement is somewhat limited as the internal study has a much larger sample size in controls and each NHL subtype group.

**Table 8:** Estimated PRS effects on four NHL subtypes based on the NHL study.

		CLL(*θ*_1_)	DLBCL(*θ*_2_)	FL(*θ*_3_)	MZL(*θ*_4_)
MLE_*int*_	Estimate	0.0187	0.0923	−0.1240	0.0474
	SE	0.0312	0.0290	0.0316	0.0463
	*P*-value	0.5495	0.0014	8.63E-05	0.3059
GIM_*opt*_ using 5 external studies	Estimate	0.0175	0.0980	−0.1236	0.0485
	SE	0.0271	0.0266	0.0292	0.0460
	*P*-value	0.5172	2.30E-04	2.28E-05	0.2923
GIM_*opt*_ using 7 external studies	Estimate	0.0155	0.0955	−0.1330	0.0462
	SE	0.0269	0.0250	0.0274	0.0459
	*P*-value	0.5641	1.37E-04	1.15E-06	0.3147

Est (SE) refers to the estimated coefficient and its standard error given by the MLE_*int*_ and GIM_*opt*_.

**Table 9:** Disease subtype heterogeneity testing *P*-values based on the NHL study.

Comparison	MLE_*int*_	GIM_*opt*_ (5 studies)	GIM_*opt*_ (7 studies)
CLL vs. DLBCL	0.0415	0.0147	0.0134
CLL vs. FL	1.94E-04	5.74E-05	1.40E-05
CLL vs. MZL	0.5735	0.5347	0.5387
DLBCL vs. FL	3.18E-09	9.55E-11	2.85E-12
DLBCL vs. MZL	0.3676	0.3136	0.3115
FL vs. MZL	0.0009	0.0007	0.0003
CLL vs. DLBCL vs. FL vs. MZL	6.14E-08	2.36E-09	6.97E-11

Each *P*-value is for testing the null hypothesis that the PRS has the same effect on different NHL subtypes.

As an experiment, we reduced the internal study sample size by randomly removing 2/3 of the control and each subtype group. Results based on this downsized internal study and the original summary data are given in S8 and S9 Tables. Compared with the results in Tables 8 and 9, the advantage of PolyGIM over MLE_*int*_ becomes more evident. Finally, we considered the following model by allowing the PRS had a nonlinear effect on each subtype,

$$\text{log}\{\frac{P(Y=k|X)}{P(Y=0|X)}\}=\omega_{k}+\theta_{k1}S\left(X\right)+\theta_{k2}S^{2}\left(X\right),\;k=1,\ldots ,4.$$

By applying PolyGIM, we found that the PRS had no significant nonlinear effect on any subtype, with *P*-values associated *θ*_*k*2_ all being larger than 0.05.

## 4 Discussion

We developed PolyGIM, an integrative procedure based on the PLR model to study a disease outcome with multiple subtypes. PolyGIM fits the PLR model using individual-level data from the internal study while incorporating constraints on the parameter space imposed by the summary data derived from external studies. The summary data consist of coefficient estimates from working logistic regression models, which can be quite general as long as their targeted binary outcomes are functions of the original multicategory outcome. Examples of the working model include the case-case comparison model, which focuses on comparing two disease subtypes, and the grouped case-control comparison model, which compares the control group with a broad disease group formed by merging several subtypes. We established the theoretic properties of the procedure and demonstrated the advantage of PolyGIM using simulation studies. We applied PolyGIM to evaluate the effect of the HL-associated PRS on the risks of four major NHL subtypes. We found that the PRS has an uneven effect on different subtypes. As shown in the NHL study, the PolyGIM procedure provides a versatile tool for exploring genetic heterogeneity by leveraging SNP-level summary statistics generated by large-scale GWAS.

We can use PolyGIM to integrate summary data from the grouped case-control comparison model, assuming we know the mixture proportion of subtypes within the broad disease group. In some practices, the mixture proportion is unknown. It is possible to expand PolyGIM by treating the mixture proportions as unknown parameters in the likelihood formation and adjusting the constraint equations accordingly. But the mixture proportions could be unidentifiable unless the risk factor has very distinct effects on different subtypes. We are still investigating strategies to incorporate this unknown mixture proportion into the PolyGIM procedure, possibly with the help of certain additional information.

We utilized PolyGIM to evaluate the impact of PRS on different NHL subtypes. Since the weights used in PRS calculation are subject to uncertainty, the PRS used in the model is essentially a variable with measurement error. However, we demonstrate that it is acceptable to ignore the measurement error by treating the observed weights as their true values for the purpose of the association testing. PolyGIM can still maintain the proper type I error using PRS with any level of measurement error. However, we should exercise caution when attempting to estimate the true effect of PRS using this approach, particularly when the variability of each weight is relatively large. Addressing measurement error in nonlinear regression models is a significant challenge, and there is no standard approach to deal with it. As a result, future research efforts could focus on developing a measurement error model for PRS and integrating it into the PolyGIM framework.

In PolyGIM, the number of unknown parameters increases linearly with the dimension of the summary data. The current version of the PolyGIM package is capable of handling several hundred SNPs simultaneously. To study a PRS with a much larger number of SNPs, one solution is to adopt the strategy proposed by [42], provided that all considered SNPs can be divided into smaller, independent batches. For instance, it is reasonable to assume that SNPs on different chromosomes are independent. This approach entails partitioning the SNPs into separate groups while ensuring the independence of SNPs within each group from those in other groups. Subsequently, PolyGIM is applied to each batch to obtain estimates of the regression coefficients. These individual estimates are then combined using a meta-analysis procedure to derive the final estimate.

PolyGIM was proposed to incorporate summary data from external studies in the situation when their individual-level data are not readily available. In fact, it is also helpful even when researchers can collect individual-level data from all studies. Suppose we want to evaluate a PRS effect on a disease outcome, as in the setting of NHL study, certain participating studies might have missing (imputed) genotypes on some SNPs required in the PRS calculation. Thus, we can not directly fit the PRS model using data from those studies since the PRS on subjects in them are undefined. On the other hand, PolyGIM can still incorporate information from those studies by using their summary statistics on measured SNPs. Therefore, PolyGIM provides a solution to the missing data problem when gathering data from multiple studies.

PolyGIM was developed to analyze individual-level and summary data from studies conducted within the same population. However, our simulation results demonstrate that it is possible to utilize partial summary data from external studies conducted in different populations under certain conditions. Moreover, we can extend PolyGIM to integrate complete summary data from different external study populations, as we did for binary outcomes in our previous work [38]. To accomplish this, we require a reference set of ***X*** randomly chosen from controls in the external study population to estimate the empirical distribution of ***X*** in that population.

Large-scale GWAS often generate SNP-level summary data using logistic or linear mixed models to account for related subjects. However, PolyGIM is formulated within an empirical likelihood framework that assumes unrelated subjects within each study, making it unable to handle summary data derived from these mixed models. To address this, a heuristic solution would be to treat the summary data from logistic mixed models as if they were generated from a standard logistic regression model, fitted on a study with an “effective sample size” of independent subjects [50]. Alternatively, for summary data generated from linear mixed models, existing methods (e.g., [51]) can be used to convert them to the corresponding logistic regression model and then proceed with PolyGIM. Nonetheless, it is important to note that both approaches are heuristic and require further evaluation.

We have developed the PolyGIM R package and made it available on GitHub for public use (https://github.com/fushengstat/PolyGIM). It can incorporate summary data sets from one external study or multiple studies with overlapping subjects. This package allows users to specify their target and working models using the R model formulae. Aided by this package, we expect PolyGIM to be a valuable tool for pooling data from multiple sources for a more coherent evaluation of disease subtype heterogeneity.

## Supporting information

S1 Appendix Supplementary material for mathematical details and technical proofs for Section 2. (PDF)

S1 Table Simulation results in situations when summary data is derived from one external study based on case-case (CC) comparison models considering the rare disease and independent markers. All numbers are multiplied by 100.
(PDF)

S2 Table Simulation results in situations when summary data is derived from one external study based on grouped case-control (GC) comparison models considering the rare disease and independent markers. All numbers are multiplied by 100.
(PDF)

S3 Table Information on the 21 SNPs used in the definition of Hodgkin lymphoma associated polygenic risk score. (PDF)

S4 TableSimulation results of type I errors under the null PRS model for the impact of measurement error.Measurement errors are considered at three different levels: none (i.e., ${\tilde{w}}_{i}=w_{i}$), low (i.e., ${\tilde{w}}_{i}∼N\left(w_{i},{\text{se}}_{i}^{2}\right)$), and high (i.e., ${\tilde{w}}_{i}∼N\left(w_{i},16{\text{se}}_{i}^{2}\right)$). Summary data are derived from five external studies with their sample sizes giving in Table 3. All numbers are multiplied by 100.(PDF)

S5 Table Simulation results on the impact of sample sizes of external studies under the alternative PRS model. We consider an internal study with a sample size reduced to 10% of its original size. Summary data are derived from five external studies, with their sample sizes varying from 1, 5 to 10 times the original sizes reported in Table 3. PRS with no or low measurement error is considered. All numbers are multiplied by 100.
(PDF)

S6 Table Summary of the average computational time (in minutes) over 100 replications under the Null PRS model. Summary data are derived from the first 1, 3, or 5 external studies shown in Table 3, with the number of SNPs varying from 21 to 105 by simply stacking the original 21 SNPs.
(PDF)

S7 Table Summary of the average memory usage (in Gigabyte) over 100 replications under the Null PRS model. Summary data are derived from the first 1, 3, or 5 external studies shown in Table 3, with the number of SNPs varying from 21 to 105 by simply stacking the original 21 SNPs.
(PDF)

S8 Table Estimated PRS effects on four NHL subtypes using 1/3 of the internal data from the NHL study. (PDF)

S9 Table Disease subtype heterogeneity testing *P*-values based on the NHL study by randomly using 1/3 of the internal data from the NHL study. Each *P*-value is for testing the null hypothesis that the PRS has the same effect on different NHL subtypes.
(PDF)

S1 FigQ-Q plots of *Z*-scores generated by GIM_*opt*_.Measurement errors for the PRS are considered at three different levels: none (i.e., ${\tilde{w}}_{i}=w_{i}$), low (i.e., ${\tilde{w}}_{i}∼N\left(w_{i},{\text{se}}_{i}^{2}\right)$), and high (i.e., ${\tilde{w}}_{i}∼N\left(w_{i},16{\text{se}}_{i}^{2}\right)$).(PDF)

S2 Fig Minor allele frequencies of the 21 SNPs in the eight NHL studies. The minor allele frequency is estimated from the control group within each study.
(PDF)
