# Supplementary material for: Improve the model of disease subtype heterogeneity by leveraging external summary data
Source: PLoS Comput Biol. 2023 Jul 12;19(7):e1011236. doi: 10.1371/journal.pcbi.1011236 (PMC10337985; doi:10.1371/journal.pcbi.1011236)
Supplement: S1 Appendix — (PDF) [file pcbi.1011236.s001.pdf]

Supplementary Material
Mathematical Formulations and Proofs for the Main Results

A Mathematical Formulations Used in the Main Text

A1 Details on Forms of A and B in Section 2.3

For CC model, we have $\mathbf{A} = \frac{1}{\rho+1}\mathbb{E}_{Y=0}\left\{\frac{\partial \boldsymbol{g}(\boldsymbol{X};\boldsymbol{\mu}^*)}{\partial(\boldsymbol{\alpha},\boldsymbol{\beta})}\right\}$ and

$$\mathbf{B} = \mathbb{E}_{Y=0}\left\{\frac{\Delta_{c_0}(\boldsymbol{X};\boldsymbol{\xi}^*)}{\rho+1}\phi_0(\boldsymbol{X};\boldsymbol{\alpha}^*,\boldsymbol{\beta}^*)\phi_0^\top(\boldsymbol{X};\boldsymbol{\alpha}^*,\boldsymbol{\beta}^*) + \frac{\rho\Delta_{c_1}(\boldsymbol{X};\boldsymbol{\xi}^*)}{\rho+1}\phi_1(\boldsymbol{X};\boldsymbol{\alpha}^*,\boldsymbol{\beta}^*)\phi_1^\top(\boldsymbol{X};\boldsymbol{\alpha}^*,\boldsymbol{\beta}^*)\right\}$$
$$-\frac{1}{\rho}\mathbb{E}_{Y=0}\{\Delta_{c_0}(\boldsymbol{X};\boldsymbol{\xi}^*)\phi_0(\boldsymbol{X};\boldsymbol{\alpha}^*,\boldsymbol{\beta}^*)\}\mathbb{E}_{Y=0}\{\Delta_{c_0}(\boldsymbol{X};\boldsymbol{\xi}^*)\phi_0(\boldsymbol{X};\boldsymbol{\alpha}^*,\boldsymbol{\beta}^*)\}^\top.$$

For GC model, we know $\mathbf{A} = \frac{1}{\rho+1}\mathbb{E}_{Y=0}\left\{\frac{\partial \boldsymbol{g}(\boldsymbol{X};\boldsymbol{\mu}^*)}{\partial(\boldsymbol{\alpha},\boldsymbol{\beta})}\right\}$ and

$$\mathbf{B} = \mathbb{E}_{Y=0}\left\{\frac{1}{\rho+1}\phi_0(\boldsymbol{X};\boldsymbol{\alpha}^*,\boldsymbol{\beta}^*)\phi_0^\top(\boldsymbol{X};\boldsymbol{\alpha}^*,\boldsymbol{\beta}^*) + \frac{\rho\sum_{k=1}^K q_k\Delta_k(\boldsymbol{X},\boldsymbol{\xi}^*)}{\rho+1}\phi_1(\boldsymbol{X};\boldsymbol{\alpha}^*,\boldsymbol{\beta}^*)\phi_1^\top(\boldsymbol{X};\boldsymbol{\alpha}^*,\boldsymbol{\beta}^*)\right\}$$
$$-\frac{1}{\rho}\mathbb{E}_{Y=0}\{\phi_0(\boldsymbol{X};\boldsymbol{\alpha}^*,\boldsymbol{\beta}^*)\}\mathbb{E}_{Y=0}\{\phi_0(\boldsymbol{X};\boldsymbol{\alpha}^*,\boldsymbol{\beta}^*)\}^\top.$$

Note that the CC and GC models have different definitions of $\boldsymbol{g}$.

A2 Details of Score Equations in Section 2.4

In particular, we can derive the following score equations to maximize the problem (11),

$$\begin{cases}
\frac{\partial \ell_{\mathbf{V}}}{\partial \lambda_k} &= -\sum_{i=1}^n \frac{\Delta_k(\boldsymbol{X}_i;\boldsymbol{\xi})-1}{1+\sum_{k=1}^K\lambda_k(\Delta_k(\boldsymbol{X}_i;\boldsymbol{\xi})-1)+\boldsymbol{\nu}^\top\boldsymbol{g}(\boldsymbol{X}_i;\boldsymbol{\mu})} = 0,\ k=1,\dots,K;\\
\frac{\partial \ell_{\mathbf{V}}}{\partial \boldsymbol{\nu}} &= -\sum_{i=1}^n \frac{\boldsymbol{g}(\boldsymbol{X}_i;\boldsymbol{\mu})}{1+\sum_{k=1}^K\lambda_k(\Delta_k(\boldsymbol{X}_i;\boldsymbol{\xi})-1)+\boldsymbol{\nu}^\top\boldsymbol{g}(\boldsymbol{X}_i;\boldsymbol{\mu})} = 0,\\
\frac{\partial \ell_{\mathbf{V}}}{\partial \tau_k} &= -\sum_{i=1}^n \frac{\lambda_k\Delta_k(\boldsymbol{X}_i;\boldsymbol{\xi})+\frac{\partial \boldsymbol{g}^\top(\boldsymbol{X}_i;\boldsymbol{\mu})}{\partial\tau_k}\boldsymbol{\nu}}{1+\sum_{k=1}^K\lambda_k(\Delta_k(\boldsymbol{X}_i;\boldsymbol{\xi})-1)+\boldsymbol{\nu}^\top\boldsymbol{g}(\boldsymbol{X}_i;\boldsymbol{\mu})} + n_k = 0,\ k=1,\dots,K;\\
\frac{\partial \ell_{\mathbf{V}}}{\partial \boldsymbol{\theta}_k} &= -\sum_{i=1}^n \frac{\lambda_k\frac{\partial\Delta_k(\boldsymbol{X}_i;\boldsymbol{\xi})}{\partial\boldsymbol{\theta}_k}+\frac{\partial \boldsymbol{g}^\top(\boldsymbol{X}_i;\boldsymbol{\mu})}{\partial\boldsymbol{\theta}_k}\boldsymbol{\nu}}{1+\sum_{k=1}^K\lambda_k(\Delta_k(\boldsymbol{X}_i;\boldsymbol{\xi})-1)+\boldsymbol{\nu}^\top\boldsymbol{g}(\boldsymbol{X}_i;\boldsymbol{\mu})} + \sum_{i=1}^n\frac{\mathbb{1}(Y_i=k)}{\Delta_k(\boldsymbol{X}_i;\boldsymbol{\xi})}\frac{\partial\Delta_k(\boldsymbol{X}_i;\boldsymbol{\xi})}{\partial\boldsymbol{\theta}_k} = 0,\ k=1,\dots,K;\\
\frac{\partial \ell_{\mathbf{V}}}{\partial \boldsymbol{\alpha}} &= -\sum_{i=1}^n \frac{\frac{\partial \boldsymbol{g}^\top(\boldsymbol{X}_i;\boldsymbol{\mu})}{\partial\boldsymbol{\alpha}}\boldsymbol{\nu}}{1+\sum_{k=1}^K\lambda_k(\Delta_k(\boldsymbol{X}_i;\boldsymbol{\xi})-1)+\boldsymbol{\nu}^\top\boldsymbol{g}(\boldsymbol{X}_i;\boldsymbol{\mu})} = 0,\\
\frac{\partial \ell_{\mathbf{V}}}{\partial \boldsymbol{\beta}} &= -\sum_{i=1}^n \frac{\frac{\partial \boldsymbol{g}^\top(\boldsymbol{X}_i;\boldsymbol{\mu})}{\partial\boldsymbol{\beta}}\boldsymbol{\nu}}{1+\sum_{k=1}^K\lambda_k(\Delta_k(\boldsymbol{X}_i;\boldsymbol{\xi})-1)+\boldsymbol{\nu}^\top\boldsymbol{g}(\boldsymbol{X}_i;\boldsymbol{\mu})} - N\mathbf{V}^{-1}(\boldsymbol{\beta}-\tilde{\boldsymbol{\beta}}) = 0.
\end{cases}$$

A3 Estimates of $\hat{\mathbf{A}}$ and $\hat{\mathbf{B}}$ in Section 2.4

For summary data from the CC model, we can estimate $\mathbf{A}$ and $\mathbf{B}$ as $\hat{\mathbf{A}} = \frac{1}{\rho+1}\sum_{i=1}^{n}\hat{p}_i\frac{\partial \boldsymbol{g}(\boldsymbol{X}_i;\hat{\boldsymbol{\mu}}_{\mathbf{V}})}{\partial(\boldsymbol{\alpha},\boldsymbol{\beta})}$ and

$$\hat{\mathbf{B}} = \sum_{i=1}^{n}\hat{p}_i\Big\{\frac{\Delta_{c_0}(\boldsymbol{X}_i;\hat{\boldsymbol{\xi}}_{\mathbf{V}})}{\rho+1}\phi_0(\boldsymbol{X}_i;\hat{\boldsymbol{\alpha}}_{\mathbf{V}},\hat{\boldsymbol{\beta}}_{\mathbf{V}})\phi_0^\top(\boldsymbol{X}_i;\hat{\boldsymbol{\alpha}}_{\mathbf{V}},\hat{\boldsymbol{\beta}}_{\mathbf{V}}) + \frac{\rho\Delta_{c_1}(\boldsymbol{X}_i;\hat{\boldsymbol{\xi}}_{\mathbf{V}})}{\rho+1}\phi_1(\boldsymbol{X}_i;\hat{\boldsymbol{\alpha}}_{\mathbf{V}},\hat{\boldsymbol{\beta}}_{\mathbf{V}})\phi_1^\top(\boldsymbol{X}_i;\hat{\boldsymbol{\alpha}}_{\mathbf{V}},\hat{\boldsymbol{\beta}}_{\mathbf{V}})\Big\}$$
$$-\frac{1}{\rho}\sum_{i=1}^{n}\hat{p}_i\{\Delta_{c_0}(\boldsymbol{X}_i;\hat{\boldsymbol{\xi}}_{\mathbf{V}})\phi_0(\boldsymbol{X}_i;\hat{\boldsymbol{\alpha}}_{\mathbf{V}},\hat{\boldsymbol{\beta}}_{\mathbf{V}})\}\sum_{i=1}^{n}\hat{p}_i\{\Delta_{c_0}(\boldsymbol{X}_i;\hat{\boldsymbol{\xi}}_{\mathbf{V}})\phi_0(\boldsymbol{X}_i;\hat{\boldsymbol{\alpha}}_{\mathbf{V}},\hat{\boldsymbol{\beta}}_{\mathbf{V}})\}^\top.$$

A4 Technical Details on Section 2.7

In this section, we provide more details on how to integrating multiple summary data. Let's assume we have summary data extracted from T external models. Suppose that the t-th external model is used to study the relationship between the outcome $D^{(t)}$ and covariates $\boldsymbol{X}^{(t)}$ based on N_{t1} cases (i.e., $D^{(t)} = 1$) and N_{t0} controls ($D^{(t)} = 0$). Here the case-control status is decided by the definition of $D^{(t)}$. One disease subtype can be treated as case in one model, but as control in a different model. Let the t-th model be

$$P(\boldsymbol{X}^{(t)}|D^{(t)} = 1) = P(\boldsymbol{X}^{(t)}|D^{(t)} = 0)\delta_t(\boldsymbol{X}^{(t)};\boldsymbol{\gamma}_t),$$

with $\delta_t(\boldsymbol{X}^{(t)};\boldsymbol{\gamma}_t) = \exp\{\alpha_{t0} + m_t(\boldsymbol{X}^{(t)};\boldsymbol{\alpha}_{t1},\boldsymbol{\beta}_t)\}$ and $\boldsymbol{\gamma}_t = (\alpha_{t0},\boldsymbol{\alpha}_{t1}^\top,\boldsymbol{\beta}_t^\top)^\top$. As we did in Section 2.3, we assume that only estimate of $\boldsymbol{\beta}_t$ is available as a part of the summary data. Let $\{\boldsymbol{X}_i^{(t)}, D_i^{(t)}, i = 1,\ldots,N_t\}$ with $N_t = N_{t0} + N_{t1}$, be the individual level data used for fitting the t-th model, and $\tilde{\boldsymbol{\gamma}}_t$ be the estimate of $\boldsymbol{\gamma}_t$. For r-th and s-th external models, we use n_{00} to denote the number of samples used as controls in both models, n_{11} as the number of samples used as cases in both models, n_{10} as the number of samples used as cases in the r-th model and as controls in the s-th model, and n_{01} as the number of samples used as controls in the r-th model and as cases in the s-th model. Note that if $n_{00} = n_{11} = n_{10} = n_{01} = 0$, there is no overlapping sample used in the two models. If $n_{00} = N_{r0} = N_{s0}$ and $n_{11} = N_{r1} = N_{s1}$, the two models are fitted with the same set of subjects. If $n_{01} = N_{r0} = N_{s1}$, all controls used in the r-th model are cases in the s-th model.

Similar to (6), estimate $\tilde{\boldsymbol{\gamma}}_t$ is obtained from the following estimating equation,

$$\boldsymbol{\Psi}_t(\boldsymbol{\gamma}_t) = \sum_{i=1}^{N_t}\left\{D_i^{(t)} - \frac{\bar{\rho}_t\delta_t(\boldsymbol{X}_i^{(t)};\boldsymbol{\gamma}_t)}{1+\bar{\rho}_t\delta_t(\boldsymbol{X}_i^{(t)};\boldsymbol{\gamma}_t)}\right\}\frac{\partial\log\delta_t(\boldsymbol{X}_i^{(t)};\boldsymbol{\gamma}_t)}{\partial(\boldsymbol{\gamma}_t)} = \mathbf{0},$$

with $\bar{\rho}_t = N_{t1}/N_{t0}$ for $t = 1,\ldots,T$. Let $\boldsymbol{\Psi}(\boldsymbol{\gamma}) = (\boldsymbol{\Psi}_1^\top(\boldsymbol{\gamma}_1),\ldots,\boldsymbol{\Psi}_T^\top(\boldsymbol{\gamma}_T))^\top$ with $\boldsymbol{\gamma} = (\boldsymbol{\gamma}_1^\top,\ldots,\boldsymbol{\gamma}_T^\top)^\top$. To allow the procedure described in Section 2.4 to incorporate regular summary data from multiple external models, we just need to find the appropriate formula for the variance-covariance matrix of

$\tilde{\boldsymbol{\gamma}} = (\tilde{\boldsymbol{\gamma}}_1^\top, \ldots, \tilde{\boldsymbol{\gamma}}_T^\top)^\top$. By the estimating equation theory of [1], we know

$$\mathrm{Cov}(\tilde{\boldsymbol{\gamma}}) = \mathbf{S}^{-1}\mathbf{T}(\mathbf{S}^{-1})^\top,$$

where $\mathbf{S} = \mathbb{E}\{\frac{\partial \boldsymbol{\Psi}(\boldsymbol{\gamma})}{\partial \boldsymbol{\gamma}^\top}\}$ and $\mathbf{T} = \mathrm{Cov}(\boldsymbol{\Psi}(\boldsymbol{\gamma}))$, with all expectations calculated under the true model. The calculation of $\mathbf{S}$ is straightforward. To evaluate $\mathbf{T}$, we need to evaluate $\mathrm{Cov}(\boldsymbol{\Psi}_r(\boldsymbol{\gamma}_r), \boldsymbol{\Psi}_s(\boldsymbol{\gamma}_s))$ for $1 \le r \le s \le T$. We have

$$\begin{aligned}
&\mathrm{Cov}(\boldsymbol{\Psi}_r(\boldsymbol{\gamma}_r), \boldsymbol{\Psi}_s(\boldsymbol{\gamma}_s)) \\
=& n_{00}\,\mathrm{Cov}_0^r\left\{\frac{\bar{\rho}_r\delta_r(\boldsymbol{X}^{(r)};\boldsymbol{\gamma}_r)}{1+\bar{\rho}_r\delta_r(\boldsymbol{X}^{(r)};\boldsymbol{\gamma}_r)}\frac{\partial \log\delta_r(\boldsymbol{X}^{(r)};\boldsymbol{\gamma}_r)}{\partial \boldsymbol{\gamma}_r}, \frac{\bar{\rho}_s\delta_s(\boldsymbol{X}^{(s)};\boldsymbol{\gamma}_s)}{1+\bar{\rho}_s\delta_s(\boldsymbol{X}^{(s)};\boldsymbol{\gamma}_s)}\frac{\partial \log\delta_s(\boldsymbol{X}^{(s)};\boldsymbol{\gamma}_s)}{\partial \boldsymbol{\gamma}_s}\right\} \\
&+ n_{01}\,\mathrm{Cov}_0^r\left\{\frac{\bar{\rho}_r\delta_r(\boldsymbol{X}^{(r)};\boldsymbol{\gamma}_r)}{1+\bar{\rho}_r\delta_r(\boldsymbol{X}^{(r)};\boldsymbol{\gamma}_r)}\frac{\partial \log\delta_r(\boldsymbol{X}^{(r)};\boldsymbol{\gamma}_r)}{\partial \boldsymbol{\gamma}_r}, \frac{1}{1+\bar{\rho}_s\delta_s(\boldsymbol{X}^{(s)};\boldsymbol{\gamma}_s)}\frac{\partial \log\delta_s(\boldsymbol{X}^{(s)};\boldsymbol{\gamma}_s)}{\partial \boldsymbol{\gamma}_s}\right\} \\
&+ n_{10}\,\mathrm{Cov}_1^r\left\{\frac{1}{1+\bar{\rho}_r\delta_r(\boldsymbol{X}^{(r)};\boldsymbol{\gamma}_r)}\frac{\partial \log\delta_r(\boldsymbol{X}^{(r)};\boldsymbol{\gamma}_r)}{\partial \boldsymbol{\gamma}_r}, \frac{\bar{\rho}_s\delta_s(\boldsymbol{X}^{(s)};\boldsymbol{\gamma}_s)}{1+\bar{\rho}_s\delta_s(\boldsymbol{X}^{(s)};\boldsymbol{\gamma}_s)}\frac{\partial \log\delta_s(\boldsymbol{X}^{(s)};\boldsymbol{\gamma}_s)}{\partial \boldsymbol{\gamma}_s}\right\} \\
&+ n_{11}\,\mathrm{Cov}_1^r\left\{\frac{1}{1+\bar{\rho}_r\delta_r(\boldsymbol{X}^{(r)};\boldsymbol{\gamma}_r)}\frac{\partial \log\delta_r(\boldsymbol{X}^{(r)};\boldsymbol{\gamma}_r)}{\partial \boldsymbol{\gamma}_r}, \frac{1}{1+\bar{\rho}_s\delta_s(\boldsymbol{X}^{(s)};\boldsymbol{\gamma}_s)}\frac{\partial \log\delta_s(\boldsymbol{X}^{(s)};\boldsymbol{\gamma}_s)}{\partial \boldsymbol{\gamma}_s}\right\}.
\end{aligned}$$

Note that in the above calculation, $\mathrm{Cov}_1^r(\cdot)$ and $\mathrm{Cov}_0^r(\cdot)$ are defined under $P(\boldsymbol{X}^{(r)}|D^{(r)} = 1)$ and $P(\boldsymbol{X}^{(r)}|D^{(r)} = 0)$, respectively. Those covariances can be calculated by converting them into expectations under $P(\boldsymbol{X}|Y = 0)$ using (2). Once the theoretic formula for $\mathrm{Cov}(\tilde{\boldsymbol{\gamma}})$ is given, the procedure described in Section 2.4 can be carried out for summary data from multiple external models.

B Proofs of the Main Theoretical Results

B1 Regularity Conditions for the Theoretical Results

We consider $\boldsymbol{\mu} = (\boldsymbol{\tau}^\top, \boldsymbol{\theta}^\top, \boldsymbol{\alpha}, \boldsymbol{\beta}^\top)^\top$ in an open set of Θ in $\mathbb{R}^m$, with $m = \dim(\boldsymbol{\tau}) + \dim(\boldsymbol{\theta}) + \dim(\boldsymbol{\alpha}) + \dim(\boldsymbol{\beta})$. We define $\boldsymbol{h}(\boldsymbol{X};\boldsymbol{\mu}) = (\Delta_1(\boldsymbol{X};\boldsymbol{\xi}) - 1, \ldots, \Delta_K(\boldsymbol{X};\boldsymbol{\xi}) - 1, \boldsymbol{g}^\top(\boldsymbol{X};\boldsymbol{\mu}))^\top$ and use $\mathbb{E}_0[\cdot] = \mathbb{E}_{Y=0}[\cdot]$ to represent the expectation in controls. The following conditions are assumed for our theoretical results. Let $\lambda_k^* = \frac{\rho_k}{1+\sum_{i=1}^K \rho_i}$ for $k = 1, \ldots, K$.

- C1. The true parameter $\boldsymbol{\mu}^*$ is in an interior point of Θ.
- C2. $\mathbb{E}_0[\boldsymbol{g}(\boldsymbol{X};\boldsymbol{\tau}^*, \boldsymbol{\theta}^*, \boldsymbol{\alpha}, \boldsymbol{\beta})] = \boldsymbol{0}$ has a unique solution at $(\boldsymbol{\alpha}^*, \boldsymbol{\beta}^*)$.
- C3. The function $\boldsymbol{h}(\boldsymbol{X};\boldsymbol{\mu})$ is twice continuously differentiable in $\boldsymbol{\mu} \in \Omega$, where $\Omega \subset \Theta$ is a neighbourhood of $\boldsymbol{\mu}^*$, and $\mathbb{E}_0\left[\left\|\frac{\boldsymbol{h}(\boldsymbol{X};\boldsymbol{\mu})}{1+\sum_{k=1}^K \lambda_k^*(\Delta_k(\boldsymbol{X};\boldsymbol{\xi})-1)}\right\|^3\right] < \infty$ and $\mathbb{E}_0\left[\left\|\frac{\boldsymbol{h}(\boldsymbol{X};\boldsymbol{\mu})\Delta_j(\boldsymbol{X};\boldsymbol{\xi})}{1+\sum_{k=1}^K \lambda_k^*(\Delta_k(\boldsymbol{X};\boldsymbol{\xi})-1)}\right\|^3\right] < \infty$ for $j = 1, \ldots, K$.

C4. $\frac{1}{n}\sum_{i=1}^{n}\frac{\boldsymbol{h}(\boldsymbol{X}_i;\boldsymbol{\mu}^*)\boldsymbol{h}^\top(\boldsymbol{X}_i;\boldsymbol{\mu}^*)}{[1+\sum_{k=1}^{K}\lambda_k^*(\Delta_k(\boldsymbol{X}_i;\boldsymbol{\xi}^*-1)]^2} \to \mathbb{E}_0\left[\frac{\boldsymbol{h}(\boldsymbol{X};\boldsymbol{\mu}^*)\boldsymbol{h}^\top(\boldsymbol{X};\boldsymbol{\mu}^*)}{1+\sum_{k=1}^{K}\lambda_k^*(\Delta_k(\boldsymbol{X};\boldsymbol{\xi}^*)-1)}\right]$ in probability, uniformly in Ω as $n \to \infty$. The matrix $\mathbb{E}\left[\frac{\boldsymbol{h}(\boldsymbol{X};\boldsymbol{\mu}^*)\boldsymbol{h}^\top(\boldsymbol{X};\boldsymbol{\mu}^*)}{1+\sum_{k=1}^{K}\lambda_k^*(\Delta_k(\boldsymbol{X};\boldsymbol{\xi}^*)-1)}\right]$ is positive.

C5. $\mathbb{E}_0\left[\left\|\partial\left\{\frac{\boldsymbol{h}(\boldsymbol{X};\boldsymbol{\mu})}{1+\sum_{k=1}^{K}\lambda_k^*(\Delta_k(\boldsymbol{X};\boldsymbol{\xi})-1)}\right\}/\partial\boldsymbol{\mu}\right\|\right] < \infty$ and $\mathbb{E}_0\left[\left\|\partial\left\{\frac{\boldsymbol{h}(\boldsymbol{X};\boldsymbol{\mu})\Delta_j(\boldsymbol{X};\boldsymbol{\xi})}{1+\sum_{k=1}^{K}\lambda_k^*(\Delta_k(\boldsymbol{X};\boldsymbol{\xi})-1)}\right\}/\partial\boldsymbol{\mu}\right\|\right] < \infty$ for $j = 1,\ldots,K$.

C6. The external estimate $\tilde{\boldsymbol{\beta}}$ satisfies $N^{1/2}(\tilde{\boldsymbol{\beta}} - \boldsymbol{\beta}^*) \xrightarrow{d} \mathcal{N}(\mathbf{0}, \boldsymbol{\Sigma}_0)$ in distribution.

All those regularity conditions can be met when standard PLR models (i.e., $M_k(\boldsymbol{X};\boldsymbol{\xi})$s and $m(\boldsymbol{X};\boldsymbol{\alpha}_1,\boldsymbol{\beta})$ are a linear function of unknown parameters) are used in internal and external studies, with both have relatively large numbers of cases and controls.

More specifically, when both internal and external studies have large enough sample sizes, we can start the iterative estimation algorithm with an initial point (i.e., the one estimated from the internal study) that is close to the true value $\boldsymbol{\mu}^*$. Therefore, it is reasonable to claim that the algorithm tries to search the solution in Θ, a neighborhood of $\boldsymbol{\mu}^*$ that is tight enough to satisfy the requirement of C2.

Condition C3 requires the smoothness of $h(\boldsymbol{X};\boldsymbol{\mu})$ and $\Delta_k(\boldsymbol{X};\boldsymbol{\xi})$s, which is clearly met when standard PLR models are used. Under Condition C3, note that $\boldsymbol{X}_i$ are i.i.d., the convergence in probability stated in C4 holds because of the law of large numbers on a study with large numbers of cases and controls. The uniformity of convergence is guaranteed by assuming Ω is a bounded closed set.

By the smoothness of $\boldsymbol{h}(\boldsymbol{X};\boldsymbol{\mu})$ and $\Delta_k(\boldsymbol{X};\boldsymbol{\xi})$s and the fact that Ω is a bounded closed set, we know that the expectation and the partial derivative are exchangeable on $\frac{\boldsymbol{h}(\boldsymbol{X};\boldsymbol{\mu})}{1+\sum_{k=1}^{K}\lambda_k^*(\Delta_k(\boldsymbol{X};\boldsymbol{\xi})-1)}$. Therefore, Condition C5 is met as $\mathbb{E}\left[\partial\left\{\frac{\boldsymbol{h}(\boldsymbol{X};\boldsymbol{\mu})}{1+\sum_{k=1}^{K}\lambda_k^*(\Delta_k(\boldsymbol{X};\boldsymbol{\xi})-1)}\right\}/\partial\boldsymbol{\mu}\right] = \partial\left\{\mathbb{E}\left[\frac{\boldsymbol{h}(\boldsymbol{X};\boldsymbol{\mu})}{1+\sum_{k=1}^{K}\lambda_k^*(\Delta_k(\boldsymbol{X};\boldsymbol{\xi})-1)}\right]\right\}/\partial\boldsymbol{\mu}$, whose elements are finite over Ω if standard logistic regression models are used.

Condition C6 is usually met if $\tilde{\boldsymbol{\beta}}$ is the ML estimate based on a standard regression model with relatively large numbers of cases and controls.

B2 Estimate Consistency

We establish the consistency of the proposed estimate as follows.

Lemma 1. *Under the same assumptions in Proposition 1 and above conditions, with probability one, $\ell_{\mathbf{V}}(\boldsymbol{\eta})$ attains its maximum value at $\hat{\boldsymbol{\eta}}_{\mathbf{V}}$, such that $\hat{\boldsymbol{\eta}}_{\mathbf{V}}$ satisfies $\partial\ell_{\mathbf{V}}(\hat{\boldsymbol{\eta}}_{\mathbf{V}})/\partial\boldsymbol{\eta} = 0$, and $\hat{\boldsymbol{\mu}}_{\mathbf{V}}$ is in the interior of the closed ball $\{\boldsymbol{\mu} : \|\boldsymbol{\mu} - \boldsymbol{\mu}^*\| \leq n^{-1/3}\}$.*

This lemma can be proved following the steps given by [2]. Details are omitted.

B3 Proof of Proposition 1

Note that the first-order derivatives of $\ell_{\mathbf{V}}(\boldsymbol{\eta})$ are given in Section A2. By expanding the score $\partial \ell_{\mathbf{V}}(\hat{\boldsymbol{\eta}}_{\mathbf{V}})/\partial \boldsymbol{\eta}$ at the true value $\boldsymbol{\eta}^* = (\boldsymbol{\lambda}^{*\top}, \mathbf{0}^\top, \boldsymbol{\tau}^{*\top}, \boldsymbol{\theta}^{*\top}, \boldsymbol{\alpha}^{*\top}, \boldsymbol{\beta}^{*\top})^\top$,

$$0 = \frac{1}{n}\frac{\partial \ell_{\mathbf{V}}(\hat{\boldsymbol{\eta}}_{\mathbf{V}})}{\partial \boldsymbol{\eta}} = \frac{1}{n}\frac{\partial \ell_{\mathbf{V}}(\boldsymbol{\eta}^*)}{\partial \boldsymbol{\eta}} + \frac{1}{n}\frac{\partial^2 \ell_{\mathbf{V}}(\boldsymbol{\eta}^*)}{\partial \boldsymbol{\eta}\partial \boldsymbol{\eta}^\top}(\hat{\boldsymbol{\eta}}_{\mathbf{V}} - \boldsymbol{\eta}^*) + o_p(\delta_n),$$

where $\delta_n = \|\hat{\boldsymbol{\eta}}_{\mathbf{V}} - \boldsymbol{\eta}^*\|$. We have

$$\hat{\boldsymbol{\eta}}_{\mathbf{V}} - \boldsymbol{\eta}^* = \mathbf{J}_{n,\mathbf{V}}^{-1}\left(\frac{1}{n}\frac{\partial \ell_{\mathbf{V}}(\boldsymbol{\eta}^*)}{\partial \boldsymbol{\eta}} + o_p(\delta_n)\right),$$

where the n^{-1} negative Hessian at the true parameter can converge to $\mathbf{J}_{\mathbf{V}}$, that is,

$$\mathbf{J}_{n,\mathbf{V}} = -\frac{1}{n}\frac{\partial^2 \ell_{\mathbf{V}}(\boldsymbol{\eta}^*)}{\partial \boldsymbol{\eta}\partial \boldsymbol{\eta}^\top} \to \mathbf{J}_{\mathbf{V}} = \begin{bmatrix} j_{\boldsymbol{\lambda}\boldsymbol{\lambda}} & j_{\boldsymbol{\lambda}\boldsymbol{v}} & j_{\boldsymbol{\lambda}\boldsymbol{\tau}} & j_{\boldsymbol{\lambda}\boldsymbol{\theta}} & 0 & 0 \\ j_{\boldsymbol{v}\boldsymbol{\lambda}} & j_{\boldsymbol{v}\boldsymbol{v}} & j_{\boldsymbol{v}\boldsymbol{\tau}} & j_{\boldsymbol{v}\boldsymbol{\theta}} & j_{\boldsymbol{v}\boldsymbol{\alpha}} & j_{\boldsymbol{v}\boldsymbol{\beta}} \\ j_{\boldsymbol{\tau}\boldsymbol{\lambda}} & j_{\boldsymbol{\tau}\boldsymbol{v}} & j_{\boldsymbol{\tau}\boldsymbol{\tau}} & j_{\boldsymbol{\tau}\boldsymbol{\theta}} & 0 & 0 \\ j_{\boldsymbol{\theta}\boldsymbol{\lambda}} & j_{\boldsymbol{\theta}\boldsymbol{v}} & j_{\boldsymbol{\theta}\boldsymbol{\tau}} & j_{\boldsymbol{\theta}\boldsymbol{\theta}} & 0 & 0 \\ 0 & j_{\boldsymbol{\alpha}\boldsymbol{v}} & 0 & 0 & 0 & 0 \\ 0 & j_{\boldsymbol{\beta}\boldsymbol{v}} & 0 & 0 & 0 & j_{\boldsymbol{\beta}\boldsymbol{\beta}} \end{bmatrix} = \begin{bmatrix} \mathbf{J}_{ss} & \mathbf{J}_{s\boldsymbol{\mu}} \\ \mathbf{J}_{\boldsymbol{\mu}s} & \mathbf{J}_{\mathbf{V},\boldsymbol{\mu}\boldsymbol{\mu}} \end{bmatrix}.$$

In specific, some straightforward computations show that

$$(j_{\boldsymbol{\lambda}\boldsymbol{\lambda}})_{st} = -\mathbb{E}\left[\frac{(\Delta_s - 1)(\Delta_t - 1)}{1 + \sum_{k=1}^K \lambda_k^*(\Delta_k - 1)}\right]$$

$$(j_{\boldsymbol{\lambda}\boldsymbol{v}})_{s\cdot} = (j_{\boldsymbol{v}\boldsymbol{\lambda}})_{\cdot s}^\top = -\mathbb{E}\left[\frac{(\Delta_s - 1)\boldsymbol{g}^\top}{1 + \sum_{k=1}^K \lambda_k^*(\Delta_k - 1)}\right]$$

$$(j_{\boldsymbol{\lambda}\boldsymbol{\tau}})_{st} = (j_{\boldsymbol{\tau}\boldsymbol{\lambda}})_{ts} = \mathbb{1}(s = t) - \mathbb{E}\left[\frac{(\Delta_s - 1)\lambda_t^*\Delta_t}{1 + \sum_{k=1}^K \lambda_k^*(\Delta_k - 1)}\right]$$

$$(j_{\boldsymbol{\lambda}\boldsymbol{\theta}})_{st} = (j_{\boldsymbol{\theta}\boldsymbol{\lambda}})_{ts} = \mathbb{1}(s = t)\cdot\mathbb{E}\left[\frac{\partial \Delta_t}{\partial \boldsymbol{\theta}_t}\right] - \mathbb{E}\left[\frac{(\Delta_s - 1)\lambda_t^*}{1 + \sum_{k=1}^K \lambda_k^*(\Delta_k - 1)}\frac{\partial \Delta_t}{\partial \boldsymbol{\theta}_t}\right]$$

$$j_{\boldsymbol{v}\boldsymbol{v}} = -\mathbb{E}\left[\frac{\boldsymbol{g}\boldsymbol{g}^\top}{1 + \sum_{k=1}^K \lambda_k^*(\Delta_k - 1)}\right]$$

$$(j_{\boldsymbol{v}\boldsymbol{\tau}})_{\cdot s} = (j_{\boldsymbol{\tau}\boldsymbol{v}})_{s\cdot} = \mathbb{E}\left[\frac{\partial \boldsymbol{g}}{\partial \tau_s}\right] - \mathbb{E}\left[\frac{\lambda_s^*\Delta_s\boldsymbol{g}}{1 + \sum_{k=1}^K \lambda_k^*(\Delta_k - 1)}\right]$$

$$(j_{\boldsymbol{v}\boldsymbol{\theta}})_{\cdot s} = (j_{\boldsymbol{\theta}\boldsymbol{v}})_{s\cdot} = \mathbb{E}\left[\frac{\partial \boldsymbol{g}}{\partial \boldsymbol{\theta}_s^\top}\right] - \mathbb{E}\left[\frac{\lambda_s^*\boldsymbol{g}}{1 + \sum_{k=1}^K \lambda_k^*(\Delta_k - 1)}\frac{\partial \Delta_s}{\partial \boldsymbol{\theta}_s^\top}\right]$$

$$j_{\boldsymbol{v}\boldsymbol{\alpha}} = j_{\boldsymbol{\alpha}\boldsymbol{v}}^\top = \mathbb{E}\left[\frac{\partial \boldsymbol{g}}{\partial \boldsymbol{\alpha}^\top}\right]$$

$$j_{\boldsymbol{v}\boldsymbol{\beta}} = j_{\boldsymbol{\beta}\boldsymbol{v}}^\top = \mathbb{E}\left[\frac{\partial \boldsymbol{g}}{\partial \boldsymbol{\beta}^\top}\right]$$

$$
\begin{aligned}
(j_{\boldsymbol{\tau}\boldsymbol{\tau}})_{st} &= \mathbb{1}(s=t)\lambda_t^* - \mathbb{E}\left[\frac{\lambda_s^*\lambda_t^*\Delta_s\Delta_t}{1+\sum_{k=1}^K \lambda_k^*(\Delta_k-1)}\right]\\
(j_{\boldsymbol{\tau}\boldsymbol{\theta}})_{st} &= (j_{\boldsymbol{\theta}\boldsymbol{\tau}})_{ts}^\top = \mathbb{1}(s=t)\cdot\mathbb{E}\left[\lambda_t^*\frac{\partial\Delta_t}{\partial\boldsymbol{\theta}_t}\right] - \mathbb{E}\left[\frac{\lambda_s^*\lambda_t^*\Delta_s\frac{\partial\Delta_t}{\partial\boldsymbol{\theta}_t}}{1+\sum_{k=1}^K \lambda_k^*(\Delta_k-1)}\right]\\
(j_{\boldsymbol{\theta}\boldsymbol{\theta}})_{st} &= \mathbb{1}(s=t)\cdot\mathbb{E}\left[\frac{\lambda_t^*\partial^2\Delta_t}{\partial\boldsymbol{\theta}_t\partial\boldsymbol{\theta}_t^\top}\right] - \mathbb{E}\left[\frac{\lambda_s^*\lambda_t^*\frac{\partial\Delta_s}{\partial\boldsymbol{\theta}_s}\frac{\partial\Delta_t}{\partial\boldsymbol{\theta}_t^\top}}{1+\sum_{k=1}^K \lambda_k^*(\Delta_k-1)}\right]\\
j_{\boldsymbol{\beta}\boldsymbol{\beta}} &= \gamma\mathbf{V}^{-1}.
\end{aligned}
$$

Similarly, the variance of $n^{-1/2}\partial\ell_{\mathbf{V}}(\boldsymbol{\eta}^*)/\partial\boldsymbol{\eta}$ can be shown to be

$$
\mathbf{I}_{\mathbf{V}} = \begin{bmatrix}
i_{\boldsymbol{\lambda}\boldsymbol{\lambda}} & i_{\boldsymbol{\lambda}\boldsymbol{\nu}} & i_{\boldsymbol{\lambda}\boldsymbol{\tau}} & i_{\boldsymbol{\lambda}\boldsymbol{\theta}} & 0 & 0\\
i_{\boldsymbol{\nu}\boldsymbol{\lambda}} & i_{\boldsymbol{\nu}\boldsymbol{\nu}} & i_{\boldsymbol{\nu}\boldsymbol{\tau}} & i_{\boldsymbol{\nu}\boldsymbol{\theta}} & 0 & 0\\
i_{\boldsymbol{\tau}\boldsymbol{\lambda}} & i_{\boldsymbol{\tau}\boldsymbol{\nu}} & i_{\boldsymbol{\tau}\boldsymbol{\tau}} & i_{\boldsymbol{\tau}\boldsymbol{\theta}} & 0 & 0\\
i_{\boldsymbol{\theta}\boldsymbol{\lambda}} & i_{\boldsymbol{\theta}\boldsymbol{\nu}} & i_{\boldsymbol{\theta}\boldsymbol{\tau}} & i_{\boldsymbol{\theta}\boldsymbol{\theta}} & 0 & 0\\
0 & 0 & 0 & 0 & 0 & 0\\
0 & 0 & 0 & 0 & 0 & i_{\boldsymbol{\beta}\boldsymbol{\beta}}
\end{bmatrix},
$$

with

$$
\begin{aligned}
(i_{\boldsymbol{\lambda}\boldsymbol{\lambda}})_{st} =& \mathbb{E}\left[\frac{(\Delta_s-1)(\Delta_t-1)}{1+\sum_{k=1}^K \lambda_k^*(\Delta_k-1)}\right] - \sum_{r=1}^K \lambda_r^*\mathbb{E}\left[\frac{(\Delta_s-1)(\Delta_r-1)}{1+\sum_{k=1}^K \lambda_k^*(\Delta_k-1)}\right]\mathbb{E}\left[\frac{(\Delta_t-1)(\Delta_r-1)}{1+\sum_{k=1}^K \lambda_k^*(\Delta_k-1)}\right]\\
&+ \sum_{q=1}^K\sum_{r=1}^K \lambda_q^*\lambda_r^*\mathbb{E}\left[\frac{(\Delta_s-1)(\Delta_q-1)}{1+\sum_{k=1}^K \lambda_k^*(\Delta_k-1)}\right]\mathbb{E}\left[\frac{(\Delta_t-1)(\Delta_r-1)}{1+\sum_{k=1}^K \lambda_k^*(\Delta_k-1)}\right],\\
(i_{\boldsymbol{\lambda}\boldsymbol{\nu}})_{s\cdot} =& (i_{\boldsymbol{\nu}\boldsymbol{\lambda}})_{\cdot s}^\top = \mathbb{E}\left[\frac{(\Delta_s-1)\boldsymbol{g}^\top}{1+\sum_{k=1}^K \lambda_k^*(\Delta_k-1)}\right] - \sum_{r=1}^K \lambda_r^*\mathbb{E}\left[\frac{(\Delta_s-1)(\Delta_r-1)}{1+\sum_{k=1}^K \lambda_k^*(\Delta_k-1)}\right]\mathbb{E}\left[\frac{(\Delta_r-1)\boldsymbol{g}^\top}{1+\sum_{k=1}^K \lambda_k^*(\Delta_k-1)}\right]\\
&+ \sum_{q=1}^K\sum_{r=1}^K \lambda_q^*\lambda_r^*\mathbb{E}\left[\frac{(\Delta_s-1)(\Delta_q-1)}{1+\sum_{k=1}^K \lambda_k^*(\Delta_k-1)}\right]\mathbb{E}\left[\frac{(\Delta_q-1)\boldsymbol{g}^\top}{1+\sum_{k=1}^K \lambda_k^*(\Delta_k-1)}\right],\\
(i_{\boldsymbol{\lambda}\boldsymbol{\tau}})_{st} =& (i_{\boldsymbol{\tau}\boldsymbol{\lambda}})_{ts} = \sum_{q=1}^K\sum_{r=1}^K \lambda_q^*\lambda_r^*\mathbb{E}\left[\frac{(\Delta_s-1)(\Delta_q-1)}{1+\sum_{k=1}^K \lambda_k^*(\Delta_k-1)}\right]\mathbb{E}\left[\frac{(\Delta_r-1)\lambda_t^*\Delta_t}{1+\sum_{k=1}^K \lambda_k^*(\Delta_k-1)} - \mathbb{1}(r=t)\right]\\
&- \sum_{r=1}^K \lambda_r^*\mathbb{E}\left[\frac{(\Delta_s-1)(\Delta_r-1)}{1+\sum_{k=1}^K \lambda_k^*(\Delta_k-1)}\right]\mathbb{E}\left[\frac{(\Delta_r-1)\lambda_t^*\Delta_t}{1+\sum_{k=1}^K \lambda_k^*(\Delta_k-1)} - \mathbb{1}(r=t)\right],\\
(i_{\boldsymbol{\lambda}\boldsymbol{\theta}})_{st} =& (i_{\boldsymbol{\theta}\boldsymbol{\lambda}})_{ts} = \sum_{q=1}^K\sum_{r=1}^K \lambda_q^*\lambda_r^*\mathbb{E}\left[\frac{(\Delta_q-1)(\Delta_s-1)}{1+\sum_{k=1}^K \lambda_k^*(\Delta_k-1)}\right]\mathbb{E}\left[\frac{(\Delta_r-1)\lambda_t^*\frac{\partial\Delta_t}{\partial\boldsymbol{\theta}_t}}{1+\sum_{k=1}^K \lambda_k^*(\Delta_k-1)} - \mathbb{1}(r=t)\frac{\partial\Delta_r}{\partial\boldsymbol{\theta}_r}\right]\\
&- \sum_{r=1}^K \lambda_r^*\mathbb{E}\left[\frac{(\Delta_s-1)(\Delta_r-1)}{1+\sum_{k=1}^K \lambda_k^*(\Delta_k-1)}\right]\mathbb{E}\left[\frac{(\Delta_r-1)\lambda_t^*\frac{\partial\Delta_t}{\partial\boldsymbol{\theta}_t}}{1+\sum_{k=1}^K \lambda_k^*(\Delta_k-1)} - \mathbb{1}(r=t)\frac{\partial\Delta_r}{\partial\boldsymbol{\theta}_r}\right],\\
i_{\boldsymbol{\nu}\boldsymbol{\nu}} =& \mathbb{E}\left[\frac{\boldsymbol{g}\boldsymbol{g}^\top}{1+\sum_{k=1}^K \lambda_k^*(\Delta_k-1)}\right] - \sum_{r=1}^K \lambda_r^*\mathbb{E}\left[\frac{(\Delta_r-1)\boldsymbol{g}}{1+\sum_{k=1}^K \lambda_k^*(\Delta_k-1)}\right]\mathbb{E}\left[\frac{(\Delta_r-1)\boldsymbol{g}^\top}{1+\sum_{k=1}^K \lambda_k^*(\Delta_k-1)}\right]
\end{aligned}
$$

$$
\begin{aligned}
&+\sum_{q=1}^{K}\sum_{r=1}^{K}\lambda_q^*\lambda_r^*\mathbb{E}\left[\frac{(\Delta_q-1)\boldsymbol{g}}{1+\sum_{k=1}^K\lambda_k^*(\Delta_k-1)}\right]\mathbb{E}\left[\frac{(\Delta_r-1)\boldsymbol{g}^\top}{1+\sum_{k=1}^K\lambda_k^*(\Delta_k-1)}\right],\\
(i_{\boldsymbol{\nu}\boldsymbol{\tau}})_{\cdot s}=&(i_{\boldsymbol{\tau}\boldsymbol{\nu}})_{s\cdot}=\sum_{q=1}^{K}\sum_{r=1}^{K}\lambda_q^*\lambda_r^*\mathbb{E}\left[\frac{(\Delta_q-1)\lambda_s^*\Delta_s}{1+\sum_{k=1}^K\lambda_k^*(\Delta_k-1)}-\mathbb{1}(q=s)\right]\mathbb{E}\left[\frac{(\Delta_r-1)\boldsymbol{g}^\top}{1+\sum_{k=1}^K\lambda_k^*(\Delta_k-1)}\right]\\
&-\sum_{r=1}^{K}\lambda_r^*\mathbb{E}\left[\frac{(\Delta_r-1)\lambda_s^*\Delta_s}{1+\sum_{k=1}^K\lambda_k^*(\Delta_k-1)}-\mathbb{1}(r=s)\right]\mathbb{E}\left[\frac{(\Delta_r-1)\boldsymbol{g}^\top}{1+\sum_{k=1}^K\lambda_k^*(\Delta_k-1)}\right],\\
(i_{\boldsymbol{\nu}\boldsymbol{\theta}})_{\cdot s}=&(i_{\boldsymbol{\theta}\boldsymbol{\nu}})_{s\cdot}=\sum_{q=1}^{K}\sum_{r=1}^{K}\lambda_q^*\lambda_r^*\mathbb{E}\left[\frac{(\Delta_q-1)\boldsymbol{g}}{1+\sum_{k=1}^K\lambda_k^*(\Delta_k-1)}\right]\mathbb{E}\left[\frac{(\Delta_r-1)\lambda_s^*\frac{\partial\Delta_s}{\partial\boldsymbol{\theta}_s^\top}}{1+\sum_{k=1}^K\lambda_k^*(\Delta_k-1)}-\mathbb{1}(r=s)\frac{\partial\Delta_r}{\partial\boldsymbol{\theta}_r^\top}\right]\\
&-\sum_{r=1}^{K}\lambda_r^*\mathbb{E}\left[\frac{(\Delta_r-1)\boldsymbol{g}}{1+\sum_{k=1}^K\lambda_k^*(\Delta_k-1)}\right]\mathbb{E}\left[\frac{(\Delta_r-1)\lambda_s^*\frac{\partial\Delta_s}{\partial\boldsymbol{\theta}_s^\top}}{1+\sum_{k=1}^K\lambda_k^*(\Delta_k-1)}-\mathbb{1}(r=s)\frac{\partial\Delta_r}{\partial\boldsymbol{\theta}_r^\top}\right],\\
(i_{\boldsymbol{\tau}\boldsymbol{\tau}})_{st}=&\mathbb{1}(s=t)\cdot\lambda_s^*-\mathbb{E}\left[\frac{\lambda_s^*\lambda_t^*\Delta_s\Delta_t}{1+\sum_{k=1}^K\lambda_k^*(\Delta_k-1)}\right]\\
&-\sum_{r=1}^{K}\lambda_r^*\mathbb{E}\left[\frac{(\Delta_r-1)\lambda_s^*\Delta_s}{1+\sum_{k=1}^K\lambda_k^*(\Delta_k-1)}-\mathbb{1}(r=s)\right]\mathbb{E}\left[\frac{(\Delta_r-1)\lambda_t^*\Delta_t}{1+\sum_{k=1}^K\lambda_k^*(\Delta_k-1)}-\mathbb{1}(r=t)\right]\\
&+\sum_{q=1}^{K}\sum_{r=1}^{K}\lambda_q^*\lambda_r^*\mathbb{E}\left[\frac{(\Delta_q-1)\lambda_s^*\Delta_s}{1+\sum_{k=1}^K\lambda_k^*(\Delta_k-1)}-\mathbb{1}(q=s)\right]\mathbb{E}\left[\frac{(\Delta_r-1)\lambda_t^*\Delta_t}{1+\sum_{k=1}^K\lambda_k^*(\Delta_k-1)}-\mathbb{1}(r=t)\right],\\
(i_{\boldsymbol{\tau}\boldsymbol{\theta}})_{st}=&(i_{\boldsymbol{\theta}\boldsymbol{\tau}})_{ts}^\top=\mathbb{1}(s=t)\cdot\mathbb{E}\left[\lambda_s^*\frac{\partial\Delta_s}{\partial\boldsymbol{\theta}_s}\right]-\mathbb{E}\left[\frac{\lambda_s^*\lambda_t^*\Delta_s\frac{\partial\Delta_t}{\partial\boldsymbol{\theta}_t}}{1+\sum_{k=1}^K\lambda_k^*(\Delta_k-1)}\right]\\
&-\sum_{r=1}^{K}\lambda_r^*\mathbb{E}\left[\frac{(\Delta_r-1)\lambda_s^*\Delta_s}{1+\sum_{k=1}^K\lambda_k^*(\Delta_k-1)}-\mathbb{1}(r=s)\right]\mathbb{E}\left[\frac{(\Delta_r-1)\lambda_t^*\frac{\partial\Delta_t}{\partial\boldsymbol{\theta}_t}}{1+\sum_{k=1}^K\lambda_k^*(\Delta_k-1)}-\mathbb{1}(r=t)\frac{\partial\Delta_r}{\partial\boldsymbol{\theta}_r}\right]\\
&+\sum_{q=1}^{K}\sum_{r=1}^{K}\lambda_q^*\lambda_r^*\mathbb{E}\left[\frac{(\Delta_q-1)\lambda_s^*\Delta_s}{1+\sum_{k=1}^K\lambda_k^*(\Delta_k-1)}-\mathbb{1}(q=s)\right]\mathbb{E}\left[\frac{(\Delta_r-1)\lambda_t^*\frac{\partial\Delta_t}{\partial\boldsymbol{\theta}_t}}{1+\sum_{k=1}^K\lambda_k^*(\Delta_k-1)}-\mathbb{1}(r=t)\frac{\partial\Delta_r}{\partial\boldsymbol{\theta}_r}\right],\\
(i_{\boldsymbol{\theta}\boldsymbol{\theta}})_{st}=&\mathbb{1}(s=t)\cdot\mathbb{E}\left[\frac{\lambda_s^*}{\Delta_s}\frac{\partial\Delta_s}{\partial\boldsymbol{\theta}_s}\frac{\partial\Delta_s}{\partial\boldsymbol{\theta}_s^\top}\right]-\mathbb{E}\left[\frac{\lambda_s^*\lambda_t^*\frac{\partial\Delta_s}{\partial\boldsymbol{\theta}_s}\frac{\partial\Delta_t}{\partial\boldsymbol{\theta}_t^\top}}{1+\sum_{k=1}^K\lambda_k^*(\Delta_k-1)}\right]\\
&-\sum_{r=1}^{K}\lambda_r^*\mathbb{E}\left[\frac{(\Delta_r-1)\lambda_s^*\frac{\partial\Delta_s}{\partial\boldsymbol{\theta}_s}}{1+\sum_{k=1}^K\lambda_k^*(\Delta_k-1)}-\mathbb{1}(r=s)\frac{\partial\Delta_r}{\partial\boldsymbol{\theta}_r}\right]\mathbb{E}\left[\frac{(\Delta_r-1)\lambda_t^*\frac{\partial\Delta_t}{\partial\boldsymbol{\theta}_t^\top}}{1+\sum_{k=1}^K\lambda_k^*(\Delta_k-1)}-\mathbb{1}(r=t)\frac{\partial\Delta_r}{\partial\boldsymbol{\theta}_r^\top}\right]\\
&+\sum_{q=1}^{K}\sum_{r=1}^{K}\lambda_q^*\lambda_r^*\mathbb{E}\left[\frac{(\Delta_q-1)\lambda_s^*\frac{\partial\Delta_s}{\partial\boldsymbol{\theta}_s}}{1+\sum_{k=1}^K\lambda_k^*(\Delta_k-1)}-\mathbb{1}(q=s)\frac{\partial\Delta_q}{\partial\boldsymbol{\theta}_q}\right]\mathbb{E}\left[\frac{(\Delta_r-1)\lambda_t^*\frac{\partial\Delta_t}{\partial\boldsymbol{\theta}_t^\top}}{1+\sum_{k=1}^K\lambda_k^*(\Delta_k-1)}-\mathbb{1}(r=t)\frac{\partial\Delta_r}{\partial\boldsymbol{\theta}_r^\top}\right],\\
i_{\boldsymbol{\beta}\boldsymbol{\beta}}=&\gamma\mathbf{V}^{-1}\boldsymbol{\Sigma}_0\mathbf{V}^{-1}.
\end{aligned}
$$

Let $\boldsymbol{\Gamma} = \mathbf{diag}(\boldsymbol{\lambda}^*) - \boldsymbol{\lambda}^*\boldsymbol{\lambda}^{*\top}$ be a matrix defined by $\boldsymbol{\lambda}^* = (\lambda_1^*, \ldots, \lambda_K^*)^\top$. One can verify that

$$\mathbf{I}_\mathbf{V} = \begin{bmatrix} -\mathbf{J}_{ss} & 0 \\ 0 & \mathbf{J}_{\mathbf{V},\boldsymbol{\mu}\boldsymbol{\mu}} \end{bmatrix} - \mathbf{J}_{\cdot\boldsymbol{\lambda}}\boldsymbol{\Gamma}\mathbf{J}_{\cdot\boldsymbol{\lambda}}^\top + \begin{bmatrix} 0 & 0 & 0 & 0 & 0 & 0 \\ 0 & 0 & 0 & 0 & 0 & 0 \\ 0 & 0 & 0 & 0 & 0 & 0 \\ 0 & 0 & 0 & 0 & 0 & 0 \\ 0 & 0 & 0 & 0 & 0 & 0 \\ 0 & 0 & 0 & 0 & 0 & i_{\boldsymbol{\beta}\boldsymbol{\beta}} - j_{\boldsymbol{\beta}\boldsymbol{\beta}} \end{bmatrix},$$

where $\mathbf{J}_{\cdot\boldsymbol{\lambda}}$ is the first column-wise block matrix of $\mathbf{J}_\mathbf{V}$.

Note that $\mathbb{E}\{\frac{\partial \ell_\mathbf{V}(\boldsymbol{\eta}^*)}{\partial \boldsymbol{\eta}}\} = 0$, from this and $\hat{\boldsymbol{\eta}}_\mathbf{V} - \boldsymbol{\eta}^* = \mathbf{J}_{n,\mathbf{V}}^{-1}\{n^{-1}\partial \ell_\mathbf{V}(\boldsymbol{\eta}^*)/\partial\boldsymbol{\eta} + o_p(\delta_n)\}$, we have $\delta_n = o_p(n^{-1/2})$. Then by central limiting theorem,

$$n^{1/2}(\hat{\boldsymbol{\eta}}_\mathbf{V} - \boldsymbol{\eta}^*) = \mathbf{J}_{n,\mathbf{V}}^{-1}\{n^{-1/2}\partial \ell_\mathbf{V}(\boldsymbol{\eta}^*)/\partial\boldsymbol{\eta}\} + o_p(1) \xrightarrow{d} \mathcal{N}(\mathbf{0}, \mathbf{J}_\mathbf{V}^{-1}\mathbf{I}_\mathbf{V}\mathbf{J}_\mathbf{V}^{-1}).$$

If $\mathbf{V} = \boldsymbol{\Sigma}_0$, then $i_{\boldsymbol{\beta}\boldsymbol{\beta}} = j_{\boldsymbol{\beta}\boldsymbol{\beta}}$, which implies that

$$\mathbf{I}_{\boldsymbol{\Sigma}_0} = \begin{bmatrix} -\mathbf{J}_{ss} & 0 \\ 0 & \mathbf{J}_{\boldsymbol{\Sigma}_0,\boldsymbol{\mu}\boldsymbol{\mu}} \end{bmatrix} - \mathbf{J}_{\cdot\boldsymbol{\lambda}}\boldsymbol{\Gamma}\mathbf{J}_{\cdot\boldsymbol{\lambda}}^\top.$$

Note the fact that $\mathbf{J}_{\boldsymbol{\Sigma}_0}^{-1}\mathbf{J}_{\cdot\boldsymbol{\lambda}} = \begin{bmatrix} \mathbf{I}_K \\ 0 \end{bmatrix}$, where $\mathbf{I}_K$ is the identity matrix of the order K. Some simple algebras show that

$$\mathbf{J}_{\boldsymbol{\Sigma}_0}^{-1}\mathbf{I}_{\boldsymbol{\Sigma}_0}\mathbf{J}_{\boldsymbol{\Sigma}_0}^{-1} = \begin{bmatrix} -\mathbf{J}_{ss}^{-1} - \mathbf{J}_{ss}^{-1}\mathbf{J}_{s\boldsymbol{\mu}}(\mathbf{J}_{\boldsymbol{\Sigma}_0,\boldsymbol{\mu}\boldsymbol{\mu}} - \mathbf{J}_{\boldsymbol{\mu}s}\mathbf{J}_{ss}^{-1}\mathbf{J}_{s\boldsymbol{\mu}})^{-1}\mathbf{J}_{\boldsymbol{\mu}s}\mathbf{J}_{ss}^{-1} & 0 \\ 0 & (\mathbf{J}_{\boldsymbol{\Sigma}_0,\boldsymbol{\mu}\boldsymbol{\mu}} - \mathbf{J}_{\boldsymbol{\mu}s}\mathbf{J}_{ss}^{-1}\mathbf{J}_{s\boldsymbol{\mu}})^{-1} \end{bmatrix} - \begin{bmatrix} \boldsymbol{\Gamma} & 0 \\ 0 & 0 \end{bmatrix}.$$

We next show the asymptotic variance of $\hat{\boldsymbol{\mu}}_{\boldsymbol{\Sigma}_0}$ can achieve the lower bound at $\mathbf{V} = \boldsymbol{\Sigma}_0$ for any positive-definite $\mathbf{V}$. Denote $\mathbf{M}_\mathbf{V} = (\mathbf{J}_{\mathbf{V},\boldsymbol{\mu}\boldsymbol{\mu}} - \mathbf{J}_{\boldsymbol{\mu}s}\mathbf{J}_{ss}^{-1}\mathbf{J}_{s\boldsymbol{\mu}})^{-1}$ and $\mathbf{M}_{\boldsymbol{\Sigma}_0} = (\mathbf{J}_{\boldsymbol{\Sigma}_0,\boldsymbol{\mu}\boldsymbol{\mu}} - \mathbf{J}_{\boldsymbol{\mu}s}\mathbf{J}_{ss}^{-1}\mathbf{J}_{s\boldsymbol{\mu}})^{-1}$. Since $n^{1/2}\hat{\boldsymbol{\eta}}_\mathbf{V} = n^{1/2}\boldsymbol{\eta}^* + \mathbf{J}_{n,\mathbf{V}}^{-1}n^{-1/2}\partial\ell_\mathbf{V}(\boldsymbol{\eta}^*)/\partial\boldsymbol{\eta} + o_p(1)$ and

$$\begin{aligned} n^{1/2}\hat{\boldsymbol{\eta}}_{\boldsymbol{\Sigma}_0} &= n^{1/2}\boldsymbol{\eta}^* + \mathbf{J}_{n,\boldsymbol{\Sigma}_0}^{-1}n^{-1/2}\partial\ell_{\boldsymbol{\Sigma}_0}(\boldsymbol{\eta}^*)/\partial\boldsymbol{\eta} + o_p(1) \\ &= n^{1/2}\boldsymbol{\eta}^* + \mathbf{J}_{n,\boldsymbol{\Sigma}_0}^{-1}\left(n^{-1/2}\partial\ell_\mathbf{V}(\boldsymbol{\eta}^*)/\partial\boldsymbol{\eta} + \begin{bmatrix} 0 \\ 0 \\ 0 \\ 0 \\ 0 \\ n^{-1/2}N(\mathbf{V}^{-1} - \boldsymbol{\Sigma}_0^{-1})(\boldsymbol{\beta} - \tilde{\boldsymbol{\beta}}) \end{bmatrix}\right) + o_p(1), \end{aligned}$$

then

$$\mathrm{Cov}(n^{1/2}\hat{\boldsymbol{\eta}}_{\mathbf{V}}, n^{1/2}\hat{\boldsymbol{\eta}}_{\boldsymbol{\Sigma}_0}) \longrightarrow \mathbf{J}_{\mathbf{V}}^{-1}\left(\mathbf{I}_{\mathbf{V}} + \begin{bmatrix} 0 & 0 & 0 & 0 & 0 & 0 \\ 0 & 0 & 0 & 0 & 0 & 0 \\ 0 & 0 & 0 & 0 & 0 & 0 \\ 0 & 0 & 0 & 0 & 0 & 0 \\ 0 & 0 & 0 & 0 & 0 & 0 \\ 0 & 0 & 0 & 0 & 0 & -(i_{\boldsymbol{\beta\beta}} - j_{\boldsymbol{\beta\beta}}) \end{bmatrix}\right)\mathbf{J}_{\boldsymbol{\Sigma}_0}^{-1}$$

$$= \begin{bmatrix} * & 0 \\ 0 & \mathbf{M}_{\mathbf{V}} \end{bmatrix} \mathbf{J}_{\mathbf{V}}\mathbf{J}_{\boldsymbol{\Sigma}_0}^{-1}$$

$$= \begin{bmatrix} * & 0 \\ 0 & \mathbf{M}_{\mathbf{V}} \end{bmatrix} \begin{bmatrix} \mathbf{J}_{ss} & \mathbf{J}_{s\boldsymbol{\mu}} \\ \mathbf{J}_{\boldsymbol{\mu}s} & \mathbf{J}_{\mathbf{V},\boldsymbol{\mu\mu}} \end{bmatrix} \begin{bmatrix} * & -\mathbf{J}_{ss}^{-1}\mathbf{J}_{s\boldsymbol{\mu}}\mathbf{M}_{\boldsymbol{\Sigma}_0} \\ * & \mathbf{M}_{\boldsymbol{\Sigma}_0} \end{bmatrix}$$

$$= \begin{bmatrix} * & * \\ * & \mathbf{M}_{\boldsymbol{\Sigma}_0} \end{bmatrix},$$

which implies $\mathrm{Cov}(n^{1/2}\hat{\boldsymbol{\mu}}_{\mathbf{V}}, n^{1/2}\hat{\boldsymbol{\mu}}_{\boldsymbol{\Sigma}_0}) \to \mathbf{M}_{\boldsymbol{\Sigma}_0}$. Since $\mathbf{M}_{\boldsymbol{\Sigma}_0}$ is the asymptotic variance of $n^{1/2}\hat{\boldsymbol{\mu}}_{\boldsymbol{\Sigma}_0}$, we have

$$\mathrm{Cov}(n^{1/2}\hat{\boldsymbol{\mu}}_{\mathbf{V}} - n^{1/2}\hat{\boldsymbol{\mu}}_{\boldsymbol{\Sigma}_0}) \to \mathrm{Cov}(n^{1/2}\hat{\boldsymbol{\mu}}_{\mathbf{V}}) - \mathbf{M}_{\boldsymbol{\Sigma}_0} \geq 0.$$

Therefore, the asymptotic variance of $n^{1/2}(\hat{\boldsymbol{\mu}}_{\mathbf{V}} - \hat{\boldsymbol{\mu}}_{\boldsymbol{\Sigma}_0})$ attains its minimum at $\mathbf{V} = \boldsymbol{\Sigma}_0$.

If $\mathbf{V}$ is a consistent estimate of $\boldsymbol{\Sigma}_0$, i.e. $\mathbf{V} - \boldsymbol{\Sigma}_0 \to 0$ with probability 1, then $\mathbf{V}^{-1} - \boldsymbol{\Sigma}_0^{-1} \to 0$ with probability 1. Using similar arguments, we have

$$n^{-1/2}\{\partial\ell_{\mathbf{V}}(\boldsymbol{\eta}^*)/\partial\boldsymbol{\beta}\} = -n^{-1/2}N(\mathbf{V}^{-1} - \boldsymbol{\Sigma}_0^{-1})(\boldsymbol{\beta} - \tilde{\boldsymbol{\beta}}) - n^{-1/2}N\boldsymbol{\Sigma}_0^{-1}(\boldsymbol{\beta} - \tilde{\boldsymbol{\beta}}).$$

Since the first term converges to 0 asymptotically, we have $\mathbf{I}_{\mathbf{V}} \to \mathbf{I}_{\boldsymbol{\Sigma}_0}$. Note that $\mathbf{J}_{n,\mathbf{V}} \to \mathbf{J}_{\boldsymbol{\Sigma}_0}$, combining these two results, we have

$$\mathrm{Cov}(n^{1/2}\hat{\boldsymbol{\eta}}_{\mathbf{V}}) \to \mathbf{J}_{\boldsymbol{\Sigma}_0}^{-1}\mathbf{I}_{\boldsymbol{\Sigma}_0}\mathbf{J}_{\boldsymbol{\Sigma}_0}^{-1},$$

the asymptotic covariance of $\mathrm{Cov}(n^{1/2}\hat{\boldsymbol{\eta}}_{\boldsymbol{\Sigma}_0})$.

Let $\boldsymbol{\xi} = (\boldsymbol{\tau}^\top, \boldsymbol{\theta}^\top)^\top$ be the vector of all parameters in the internal study. To simplify the notation, here we always consider $\mathbf{V} = \boldsymbol{\Sigma}_0$. Let $\mathbf{M}' = (j_{\boldsymbol{\nu\nu}} - j_{\boldsymbol{\nu\lambda}} j_{\boldsymbol{\lambda\lambda}}^{-1} j_{\boldsymbol{\lambda\nu}})^{-1}$ and $\boldsymbol{\gamma} = (\boldsymbol{\alpha}^\top, \boldsymbol{\beta}^\top)^\top$, we have

$$\mathrm{Cov}(\hat{\boldsymbol{\xi}}_{\boldsymbol{\Sigma}_0}) = [(\mathbf{J}_{\boldsymbol{\Sigma}_0,\boldsymbol{\mu\mu}} - \mathbf{J}_{\boldsymbol{\mu}s}\mathbf{J}_{ss}^{-1}\mathbf{J}_{s\boldsymbol{\mu}})^{-1}]_{\boldsymbol{\xi\xi}}$$

$$= \begin{bmatrix} \mathbf{J}_{\boldsymbol{\xi\xi}} - \mathbf{J}_{\boldsymbol{\xi}s}\mathbf{J}_{ss}^{-1}\mathbf{J}_{s\boldsymbol{\xi}} & -\mathbf{J}_{\boldsymbol{\xi}s}\mathbf{J}_{ss}^{-1}\mathbf{J}_{s\boldsymbol{\gamma}} \\ -\mathbf{J}_{\boldsymbol{\gamma}s}\mathbf{J}_{ss}^{-1}\mathbf{J}_{s\boldsymbol{\xi}} & \begin{bmatrix} 0 & 0 \\ 0 & \gamma\boldsymbol{\Sigma}_0^{-1} \end{bmatrix} - \mathbf{J}_{\boldsymbol{\gamma}s}\mathbf{J}_{ss}^{-1}\mathbf{J}_{s\boldsymbol{\gamma}} \end{bmatrix}_{\boldsymbol{\xi\xi}}^{-1}$$

$$= \begin{bmatrix} \mathbf{J}_{\xi\xi} - \mathbf{J}_{\xi s}\mathbf{J}_{ss}^{-1}\mathbf{J}_{s\xi} & -\mathbf{J}_{\xi s}\mathbf{J}_{ss}^{-1}\mathbf{J}_{s\gamma} \\ -\mathbf{J}_{\gamma s}\mathbf{J}_{ss}^{-1}\mathbf{J}_{s\xi} & \begin{bmatrix} 0 & 0 \\ 0 & \gamma\boldsymbol{\Sigma}_0^{-1} \end{bmatrix} - \mathbf{J}_{\gamma\nu}\mathbf{M}'\mathbf{J}_{\nu\gamma} \end{bmatrix}^{-1}_{\xi\xi}$$

$$= \left(\mathbf{J}_{\xi\xi} - \mathbf{J}_{\xi s}\mathbf{J}_{ss}^{-1}\mathbf{J}_{s\xi} - \mathbf{J}_{\xi s}\mathbf{J}_{ss}^{-1}\mathbf{J}_{s\gamma} \left(\begin{bmatrix} 0 & 0 \\ 0 & \gamma\boldsymbol{\Sigma}_0^{-1} \end{bmatrix} - \mathbf{J}_{\gamma\nu}\mathbf{M}'\mathbf{J}_{\nu\gamma} \right)^{-1} \mathbf{J}_{\gamma s}\mathbf{J}_{ss}^{-1}\mathbf{J}_{s\xi} \right)^{-1}$$

$$= \left(\mathbf{J}_{\xi\xi} + \mathbf{J}_{\xi s}\mathbf{J}_{ss}^{-1} \left(-\mathbf{J}_{ss} - \begin{bmatrix} 0 & 0 \\ 0 & \left(-\mathbf{M}' + \mathbf{J}_{\gamma\nu}^{-1} \begin{bmatrix} 0 & 0 \\ 0 & \gamma\boldsymbol{\Sigma}_0^{-1} \end{bmatrix} \mathbf{J}_{\nu\gamma}^{-1} \right)^{-1} \end{bmatrix} \right) \mathbf{J}_{ss}^{-1}\mathbf{J}_{s\xi} \right)^{-1}.$$

Let $\boldsymbol{\Gamma}' = \mathbf{diag}(\frac{1}{\lambda_1^*}, \ldots, \frac{1}{\lambda_K^*}) + \frac{1}{1-\sum_k \lambda_k^*}\mathbf{1}\mathbf{1}^\top$ be a $K \times K$ matrix. Then, we have $\mathbf{J}_{\boldsymbol{\lambda}\xi} = \boldsymbol{\Gamma}'\mathbf{J}_{\boldsymbol{\tau}\xi}$ and the variance of the internal based MLE $\hat{\boldsymbol{\xi}}_{mle}$ as defined by (4) is

$$\text{Cov}(\hat{\boldsymbol{\xi}}_{mle}) = \mathbf{J}_{\xi\xi}^{-1}\mathbf{I}_{\xi\xi}\mathbf{J}_{\xi\xi}^{-1} = \mathbf{J}_{\xi\xi}^{-1} - \begin{bmatrix} \boldsymbol{\Gamma}' & 0 \\ 0 & 0 \end{bmatrix}.$$

Since $-\mathbf{J}_{ss} > 0$ and $-\mathbf{M}' > 0$, we know $-j_{\boldsymbol{\lambda}\boldsymbol{\lambda}} > 0$ and

$$\mathbf{M}_1 \triangleq -\mathbf{J}_{ss} - \begin{bmatrix} 0 & 0 \\ 0 & \left(-\mathbf{M}' + \mathbf{J}_{\gamma\nu}^{-1} \begin{bmatrix} 0 & 0 \\ 0 & \gamma\boldsymbol{\Sigma}_0^{-1} \end{bmatrix} \mathbf{J}_{\nu\gamma}^{-1} \right)^{-1} \end{bmatrix} \geq \begin{bmatrix} -j_{\boldsymbol{\lambda}\boldsymbol{\lambda}} & -j_{\boldsymbol{\lambda}\nu} \\ -j_{\nu\boldsymbol{\lambda}} & -j_{\nu\boldsymbol{\lambda}} j_{\boldsymbol{\lambda}\boldsymbol{\lambda}}^{-1} j_{\boldsymbol{\lambda}\nu} \end{bmatrix} = \mathbf{J}_{s\boldsymbol{\lambda}}(-j_{\boldsymbol{\lambda}\boldsymbol{\lambda}})^{-1}\mathbf{J}_{\boldsymbol{\lambda}s} \geq 0.$$

Based on some tedious matrix algebras, we have

$$\begin{aligned} \text{Cov}(\hat{\boldsymbol{\xi}}_{\boldsymbol{\Sigma}_0}) = (\mathbf{J}_{\xi\xi} + \mathbf{J}_{\xi s}\mathbf{J}_{ss}^{-1}\mathbf{M}_1\mathbf{J}_{ss}^{-1}\mathbf{J}_{s\xi})^{-1} &\leq \left(\mathbf{J}_{\xi\xi} - \mathbf{J}_{\xi s} \begin{bmatrix} j_{\boldsymbol{\lambda}\boldsymbol{\lambda}}^{-1} & 0 \\ 0 & 0 \end{bmatrix} \mathbf{J}_{s\xi} \right)^{-1} \\ &= (\mathbf{J}_{\xi\xi} - \mathbf{J}_{\xi\boldsymbol{\lambda}} j_{\boldsymbol{\lambda}\boldsymbol{\lambda}}^{-1} \mathbf{J}_{\boldsymbol{\lambda}\xi})^{-1} \\ &= \mathbf{J}_{\xi\xi}^{-1} + \mathbf{J}_{\xi\xi}^{-1}\mathbf{J}_{\xi\boldsymbol{\lambda}}(j_{\boldsymbol{\lambda}\boldsymbol{\lambda}} - \mathbf{J}_{\boldsymbol{\lambda}\xi}\mathbf{J}_{\xi\xi}^{-1}\mathbf{J}_{\xi\boldsymbol{\lambda}})^{-1}\mathbf{J}_{\xi\boldsymbol{\lambda}}\mathbf{J}_{\xi\xi}^{-1} \\ &= \mathbf{J}_{\xi\xi}^{-1} + \begin{bmatrix} \boldsymbol{\Gamma}' \\ 0 \end{bmatrix} (j_{\boldsymbol{\lambda}\boldsymbol{\lambda}} - \boldsymbol{\Gamma}' j_{\boldsymbol{\tau}\boldsymbol{\lambda}})^{-1} \begin{bmatrix} \boldsymbol{\Gamma}' & 0 \end{bmatrix} \\ &= \mathbf{J}_{\xi\xi}^{-1} + \begin{bmatrix} \boldsymbol{\Gamma}' \\ 0 \end{bmatrix} (-\boldsymbol{\Gamma}')^{-1} \begin{bmatrix} \boldsymbol{\Gamma}' & 0 \end{bmatrix} \\ &= \mathbf{J}_{\xi\xi}^{-1} - \begin{bmatrix} \boldsymbol{\Gamma}' & 0 \\ 0 & 0 \end{bmatrix}, \end{aligned}$$

which implies $\text{Cov}(\hat{\boldsymbol{\xi}}_{\boldsymbol{\Sigma}_0}) \leq \text{Cov}(\hat{\boldsymbol{\xi}}_{mle})$. This completes the proof of Proposition 1. $\square$

B4 Proof of Proposition 2

First, we should modify the regularity conditions of Appendix B1 for irregular summary data. We consider $\boldsymbol{\xi} = (\boldsymbol{\tau}^\top, \boldsymbol{\theta}^\top)^\top$ in an open set of Θ in $\mathbb{R}^m$ with $m = \dim(\boldsymbol{\tau}) + \dim(\boldsymbol{\theta})$, and define $\bar{\boldsymbol{h}}(\boldsymbol{X};\boldsymbol{\xi}) = (\Delta_1(\boldsymbol{X};\boldsymbol{\xi}) - 1, \ldots, \Delta_K(\boldsymbol{X};\boldsymbol{\xi}) - 1)^\top$. The following conditions are assumed for the our proofs.

C1′. The true parameter $\boldsymbol{\xi}^*$ is in an interior point of Θ.

C2′. The function $\bar{\boldsymbol{h}}(\boldsymbol{X};\boldsymbol{\xi})$ is twice continuously differentiable in $\boldsymbol{\xi} \in \Omega$, where $\Omega \subset \Theta$ is a neighbourhood of $\boldsymbol{\xi}^*$, and $\mathbb{E}_0\left[\left\|\frac{\bar{\boldsymbol{h}}(\boldsymbol{X};\boldsymbol{\xi})}{1+\sum_{k=1}^K \lambda_k^*(\Delta_k(\boldsymbol{X};\boldsymbol{\xi})-1)}\right\|^3\right] < \infty$ and $\mathbb{E}_0\left[\left\|\frac{\bar{\boldsymbol{h}}(\boldsymbol{X};\boldsymbol{\xi})\Delta_j(\boldsymbol{X};\boldsymbol{\xi})}{1+\sum_{k=1}^K \lambda_k^*(\Delta_k(\boldsymbol{X};\boldsymbol{\xi})-1)}\right\|^3\right] < \infty$ for $j = 1, \ldots, K$.

C3′. $\frac{1}{n}\sum_{i=1}^n \frac{\bar{\boldsymbol{h}}(\boldsymbol{X}_i;\boldsymbol{\xi}^*)\bar{\boldsymbol{h}}^\top(\boldsymbol{X}_i;\boldsymbol{\xi}^*)}{[1+\sum_{k=1}^K \lambda_k^*(\Delta_k(\boldsymbol{X}_i;\boldsymbol{\xi}^*-1)]^2} \to \mathbb{E}_0\left[\frac{\bar{\boldsymbol{h}}(\boldsymbol{X};\boldsymbol{\xi}^*)\bar{\boldsymbol{h}}^\top(\boldsymbol{X};\boldsymbol{\xi}^*)}{1+\sum_{k=1}^K \lambda_k^*(\Delta_k(\boldsymbol{X};\boldsymbol{\xi}^*)-1)}\right]$ in probability, uniformly in Ω as $n \to \infty$. The matrix $\mathbb{E}\left[\frac{\bar{\boldsymbol{h}}(\boldsymbol{X};\boldsymbol{\xi}^*)\bar{\boldsymbol{h}}^\top(\boldsymbol{X};\boldsymbol{\xi}^*)}{1+\sum_{k=1}^K \lambda_k^*(\Delta_k(\boldsymbol{X};\boldsymbol{\xi}^*)-1)}\right]$ is positive.

C4′. $\mathbb{E}_0\left[\left\|\partial\left\{\frac{\bar{\boldsymbol{h}}(\boldsymbol{X};\boldsymbol{\xi})}{1+\sum_{k=1}^K \lambda_k^*(\Delta_k(\boldsymbol{X};\boldsymbol{\xi})-1)}\right\}/\partial\boldsymbol{\mu}\right\|\right] < \infty$ and $\mathbb{E}_0\left[\left\|\partial\left\{\frac{\bar{\boldsymbol{h}}(\boldsymbol{X};\boldsymbol{\xi})\Delta_j(\boldsymbol{X};\boldsymbol{\xi})}{1+\sum_{k=1}^K \lambda_k^*(\Delta_k(\boldsymbol{X};\boldsymbol{\xi})-1)}\right\}/\partial\boldsymbol{\mu}\right\|\right] < \infty$ for $j = 1, \ldots, K$.

C5′. The external estimate $\tilde{\boldsymbol{\beta}}$ satisfies $N^{1/2}(\tilde{\boldsymbol{\beta}} - \boldsymbol{\theta}_{c_1}^* + \boldsymbol{\theta}_{c_0}^*) \xrightarrow{d} \mathcal{N}(\mathbf{0}, \boldsymbol{\Sigma}_0)$ in distribution.

Similar to Lemma 1, we can establish the consistency of $\hat{\boldsymbol{\xi}}_{\mathbf{V}}$. Next, we start to prove the main results.

Proof: The first-order derivatives of $\ell'_{\mathbf{V}}(\boldsymbol{\xi})$ are

$$
\begin{aligned}
\frac{\partial \ell'_{\mathbf{V}}}{\partial \tau_k} &= -\sum_{i=1}^n \frac{\rho_k \Delta_k(\boldsymbol{X}_i;\boldsymbol{\xi})}{1+\sum_{k=1}^K \rho_k \Delta_k(\boldsymbol{X}_i;\boldsymbol{\xi})} + n_k, \\
\frac{\partial \ell'_{\mathbf{V}}}{\partial \boldsymbol{\theta}_k} &= -\sum_{i=1}^n \frac{\rho_k \frac{\partial \Delta_k(\boldsymbol{X}_i;\boldsymbol{\xi})}{\partial \boldsymbol{\theta}_k}}{1+\sum_{k=1}^K \rho_k \Delta_k(\boldsymbol{X}_i;\boldsymbol{\xi})} + \sum_{i=1}^n \mathbb{1}(Y_i = k) \frac{1}{\Delta_k(\boldsymbol{X}_i;\boldsymbol{\xi})} \frac{\partial \Delta_k(\boldsymbol{X}_i;\boldsymbol{\xi})}{\partial \boldsymbol{\theta}_k} \\
&\quad - [\mathbb{1}(k = c_1) - \mathbb{1}(k = c_0)] \cdot N\mathbf{V}^{-1}(\boldsymbol{\theta}_{c_1} - \boldsymbol{\theta}_{c_0} - \tilde{\boldsymbol{\beta}}).
\end{aligned}
$$

By expanding the score $\partial \ell'_{\mathbf{V}}(\hat{\boldsymbol{\xi}}_{\mathbf{V}})/\partial \boldsymbol{\xi}$ at the true value $\boldsymbol{\xi}^* = (\boldsymbol{\tau}^{*\top}, \boldsymbol{\theta}^{*\top})^\top$,

$$
0 = \frac{1}{n}\frac{\partial \ell'_{\mathbf{V}}(\hat{\boldsymbol{\xi}}_{\mathbf{V}})}{\partial \boldsymbol{\xi}} = \frac{1}{n}\frac{\partial \ell'_{\mathbf{V}}(\boldsymbol{\xi}^*)}{\partial \boldsymbol{\xi}} + \frac{1}{n}\frac{\partial^2 \ell'_{\mathbf{V}}(\boldsymbol{\xi}^*)}{\partial \boldsymbol{\xi}\partial \boldsymbol{\xi}^\top}(\hat{\boldsymbol{\xi}}_{\mathbf{V}} - \boldsymbol{\xi}^*) + o_p(\delta_n),
$$

where $\delta_n = \|\hat{\boldsymbol{\xi}}_{\mathbf{V}} - \boldsymbol{\xi}^*\|$. We have

$$
\hat{\boldsymbol{\xi}}_{\mathbf{V}} - \boldsymbol{\xi}^* = \mathbf{J}_{n,\mathbf{V}}'^{-1}\left(\frac{1}{n}\frac{\partial \ell'_{\mathbf{V}}(\boldsymbol{\xi}^*)}{\partial \boldsymbol{\xi}} + o_p(\delta_n)\right),
$$

where the n^{-1} negative Hessian at the true parameter can converge to $\mathbf{J}'_{\mathbf{V}}$, that is,

$$\mathbf{J}'_{n,\mathbf{V}} = -\frac{1}{n}\frac{\partial^2 \ell'_{\mathbf{V}}(\boldsymbol{\xi}^*)}{\partial \boldsymbol{\xi} \partial \boldsymbol{\xi}^\top} \to \mathbf{J}'_{\mathbf{V}} = \begin{bmatrix} j_{\boldsymbol{\tau}\boldsymbol{\tau}} & j_{\boldsymbol{\tau}\boldsymbol{\theta}} \\ j_{\boldsymbol{\theta}\boldsymbol{\tau}} & j'_{\boldsymbol{\theta}\boldsymbol{\theta}} \end{bmatrix},$$

with $j'_{\boldsymbol{\theta}\boldsymbol{\theta}} = j_{\boldsymbol{\theta}\boldsymbol{\theta}} + [\mathbb{1}(s=c_1) - \mathbb{1}(s=c_0)][\mathbb{1}(t=c_1) - \mathbb{1}(t=c_0)] \cdot \gamma \mathbf{V}^{-1}$. Here, $j_{\boldsymbol{\tau}\boldsymbol{\tau}}$, $j_{\boldsymbol{\tau}\boldsymbol{\theta}}$ and $j_{\boldsymbol{\theta}\boldsymbol{\theta}}$ are identical to that of Appendix B3.

Similarly, the variance of $n^{-1/2}\partial \ell'_{\mathbf{V}}(\boldsymbol{\eta}^*)/\partial \boldsymbol{\eta}$ can be shown to be

$$\mathbf{I}'_{\mathbf{V}} = \begin{bmatrix} i_{\boldsymbol{\tau}\boldsymbol{\tau}} & i_{\boldsymbol{\tau}\boldsymbol{\theta}} \\ i_{\boldsymbol{\theta}\boldsymbol{\tau}} & i'_{\boldsymbol{\theta}\boldsymbol{\theta}} \end{bmatrix},$$

with $i'_{\boldsymbol{\theta}\boldsymbol{\theta}} = i_{\boldsymbol{\theta}\boldsymbol{\theta}} + [\mathbb{1}(s=c_1) - \mathbb{1}(s=c_0)][\mathbb{1}(t=c_1) - \mathbb{1}(t=c_0)] \cdot \gamma \mathbf{V}^{-1}\boldsymbol{\Sigma}_0\mathbf{V}^{-1}$. Note that $i_{\boldsymbol{\tau}\boldsymbol{\tau}}$, $i_{\boldsymbol{\tau}\boldsymbol{\theta}}$ and $i_{\boldsymbol{\theta}\boldsymbol{\theta}}$ are the same as defined in Appendix B3.

Recall the definition of $\boldsymbol{\Gamma}'$ in Appendix B3, one can verify that

$$\mathbf{I}'_{\mathbf{V}} = \mathbf{J}'_{\mathbf{V}} - \mathbf{J}'_{\cdot\boldsymbol{\tau}}\boldsymbol{\Gamma}'\mathbf{J}'^{\top}_{\cdot\boldsymbol{\tau}} + \begin{bmatrix} 0 & 0 \\ 0 & \mathbf{A} \end{bmatrix},$$

where $\mathbf{J}'_{\cdot\boldsymbol{\tau}}$ is the first column-wise block matrix of $\mathbf{J}'_{\mathbf{V}}$, and $\mathbf{A}$ is a $K \times K$ block matrix with the (s,t)-th block $[\mathbb{1}(s=c_1) - \mathbb{1}(s=c_0)][\mathbb{1}(t=c_1) - \mathbb{1}(t=c_0)] \cdot \gamma \mathbf{V}^{-1}\boldsymbol{\Sigma}_0(\mathbf{V}^{-1} - \boldsymbol{\Sigma}_0^{-1})$.

Note that $\mathbb{E}[\partial \ell'_{\mathbf{V}}(\boldsymbol{\xi}^*)/\partial \boldsymbol{\xi}] = 0$, from this and $\hat{\boldsymbol{\xi}}_{\mathbf{V}} - \boldsymbol{\xi}^* = \mathbf{J}'^{-1}_{n,\mathbf{V}}\{n^{-1}\partial \ell'_{\mathbf{V}}(\boldsymbol{\xi}^*)/\partial \boldsymbol{\xi} + o_p(\delta_n)\}$, we have $\delta_n = o_p(n^{-1/2})$. Then by central limiting theorem,

$$n^{1/2}(\hat{\boldsymbol{\xi}}_{\mathbf{V}} - \boldsymbol{\xi}^*) = \mathbf{J}'^{-1}_{n,\mathbf{V}}\{n^{-1/2}\partial \ell'_{\mathbf{V}}(\boldsymbol{\xi}^*)/\partial \boldsymbol{\xi}\} + o_p(1) \xrightarrow{d} \mathcal{N}(\mathbf{0}, \mathbf{J}'^{-1}_{\mathbf{V}}\mathbf{I}'_{\mathbf{V}}\mathbf{J}'^{-1}_{\mathbf{V}}).$$

If $\mathbf{V} = \boldsymbol{\Sigma}_0$, then $\mathbf{V}^{-1} = \boldsymbol{\Sigma}_0^{-1}$ and $\mathbf{A} = \mathbf{0}$, which implies that

$$\mathbf{I}'_{\boldsymbol{\Sigma}_0} = \mathbf{J}'_{\boldsymbol{\Sigma}_0} - \mathbf{J}'_{\cdot\boldsymbol{\tau}}\boldsymbol{\Gamma}'\mathbf{J}'^{\top}_{\cdot\boldsymbol{\tau}}.$$

Note the fact that $\mathbf{J}'^{-1}_{\boldsymbol{\Sigma}_0}\mathbf{J}'_{\cdot\boldsymbol{\tau}} = \begin{bmatrix} \mathbf{I}_K \\ 0 \end{bmatrix}$, where $\mathbf{I}_K$ is the identity matrix of the order K. Some simple algebras show that

$$\mathbf{J}'^{-1}_{\boldsymbol{\Sigma}_0}\mathbf{I}'_{\boldsymbol{\Sigma}_0}\mathbf{J}'^{-1}_{\boldsymbol{\Sigma}_0} = \mathbf{J}'^{-1}_{\boldsymbol{\Sigma}_0} - \begin{bmatrix} \boldsymbol{\Gamma}' & 0 \\ 0 & 0 \end{bmatrix} \triangleq \mathbf{M}_{\boldsymbol{\Sigma}_0}.$$

We next show the asymptotic variance of $\hat{\boldsymbol{\xi}}_{\boldsymbol{\Sigma}_0}$ can achieve the lower bound at $\mathbf{V} = \boldsymbol{\Sigma}_0$ for any positive-definite $\mathbf{V}$. Since

$$n^{1/2}\hat{\boldsymbol{\xi}}_{\mathbf{V}} = n^{1/2}\boldsymbol{\xi}^* + \mathbf{J}'^{-1}_{n,\mathbf{V}}n^{-1/2}\partial \ell'_{\mathbf{V}}(\boldsymbol{\xi}^*)/\partial \boldsymbol{\xi} + o_p(1),$$

$$n^{1/2}\hat{\boldsymbol{\xi}}_{\boldsymbol{\Sigma}_0} = n^{1/2}\boldsymbol{\xi}^* + \mathbf{J}'^{-1}_{n,\boldsymbol{\Sigma}_0} n^{-1/2}\partial\ell'_{\boldsymbol{\Sigma}_0}(\boldsymbol{\xi}^*)/\partial\boldsymbol{\xi} + o_p(1)$$
$$= n^{1/2}\boldsymbol{\xi}^* + \mathbf{J}'^{-1}_{n,\boldsymbol{\Sigma}_0}\left(n^{-1/2}\partial\ell'_{\mathbf{V}}(\boldsymbol{\xi}^*)/\partial\boldsymbol{\xi} + \begin{bmatrix}0\\ \boldsymbol{a}\end{bmatrix}\right) + o_p(1),$$

where the k-th subvector of $\boldsymbol{a}$ is $[\mathbb{1}(k=c_1)-\mathbb{1}(k=c_0)]\cdot n^{-1/2}N(\mathbf{V}^{-1}-\boldsymbol{\Sigma}_0^{-1})(\boldsymbol{\theta}_{c_1}-\boldsymbol{\theta}_{c_0}-\tilde{\boldsymbol{\beta}})$. Thus,

$$\begin{aligned}\mathrm{Cov}(n^{1/2}\hat{\boldsymbol{\xi}}_{\mathbf{V}}, n^{1/2}\hat{\boldsymbol{\xi}}_{\boldsymbol{\Sigma}_0}) \longrightarrow & \mathbf{J}'^{-1}_{\mathbf{V}}\left(\mathbf{I}'_{\mathbf{V}} + \begin{bmatrix}0 & 0\\ 0 & -\mathbf{A}\end{bmatrix}\right)\mathbf{J}'^{-1}_{\boldsymbol{\Sigma}_0}\\ =&\mathbf{J}'^{-1}_{\boldsymbol{\Sigma}_0} - \begin{bmatrix}\boldsymbol{\Gamma}' & 0\\ 0 & 0\end{bmatrix}\mathbf{J}'_{\mathbf{V}}\mathbf{J}'^{-1}_{\boldsymbol{\Sigma}_0}\\ =&\mathbf{J}'^{-1}_{\boldsymbol{\Sigma}_0} - \begin{bmatrix}\boldsymbol{\Gamma}' & 0\\ 0 & 0\end{bmatrix}\begin{bmatrix}j_{\boldsymbol{\tau\tau}} & j_{\boldsymbol{\tau\theta}}\\ * & *\end{bmatrix}\begin{bmatrix}j_{\boldsymbol{\tau\tau}} & j_{\boldsymbol{\tau\theta}}\\ j_{\boldsymbol{\tau\theta}} & j'_{\boldsymbol{\Sigma}_0,\boldsymbol{\tau\theta}}\end{bmatrix}^{-1}\\ =&\mathbf{J}'^{-1}_{\boldsymbol{\Sigma}_0} - \begin{bmatrix}\boldsymbol{\Gamma}' & 0\\ 0 & 0\end{bmatrix} = \mathbf{M}_{\boldsymbol{\Sigma}_0}.\end{aligned}$$

which implies $\mathrm{Cov}(n^{1/2}\hat{\boldsymbol{\xi}}_{\mathbf{V}}, n^{1/2}\hat{\boldsymbol{\xi}}_{\boldsymbol{\Sigma}_0}) \to \mathbf{M}_{\boldsymbol{\Sigma}_0}$. Since $\mathbf{M}_{\boldsymbol{\Sigma}_0}$ is the asymptotic variance of $n^{1/2}\hat{\boldsymbol{\xi}}_{\boldsymbol{\Sigma}_0}$, we have

$$\mathrm{Cov}(n^{1/2}\hat{\boldsymbol{\xi}}_{\mathbf{V}} - n^{1/2}\hat{\boldsymbol{\xi}}_{\boldsymbol{\Sigma}_0}) \to \mathrm{Cov}(n^{1/2}\hat{\boldsymbol{\xi}}_{\mathbf{V}}) - \mathbf{M}_{\boldsymbol{\Sigma}_0} \geq 0.$$

Therefore, the asymptotic variance of $n^{1/2}\hat{\boldsymbol{\xi}}_{\mathbf{V}}$ attains its minimum at $\mathbf{V}=\boldsymbol{\Sigma}_0$.

If $\mathbf{V}$ is a consistent estimate of $\boldsymbol{\Sigma}_0$, i.e. $\mathbf{V}-\boldsymbol{\Sigma}_0 \to 0$ with probability 1, then $\mathbf{V}^{-1}-\boldsymbol{\Sigma}_0^{-1} \to 0$ with probability 1. Using similar arguments in proof of Proposition 1, we have

$$n^{-1/2}\{\partial\ell'_{\mathbf{V}}(\boldsymbol{\xi}^*)/\partial\boldsymbol{\theta}\} - n^{-1/2}\{\partial\ell'_{\boldsymbol{\Sigma}_0}(\boldsymbol{\xi}^*)/\partial\boldsymbol{\theta}\} = -\boldsymbol{a}.$$

Since $\boldsymbol{a}$ converges to $\mathbf{0}$ asymptotically, we have $\mathbf{I}'_{\mathbf{V}} \to \mathbf{I}'_{\boldsymbol{\Sigma}_0}$. Note that $\mathbf{J}'_{n,\mathbf{V}} \to \mathbf{J}'_{\boldsymbol{\Sigma}_0}$, combining these two results, we have

$$\mathrm{Cov}(n^{1/2}\hat{\boldsymbol{\xi}}_{\mathbf{V}}) \to \mathbf{J}'^{-1}_{\boldsymbol{\Sigma}_0}\mathbf{I}'_{\boldsymbol{\Sigma}_0}\mathbf{J}'^{-1}_{\boldsymbol{\Sigma}_0},$$

the asymptotic covariance of $\mathrm{Cov}(n^{1/2}\hat{\boldsymbol{\xi}}_{\boldsymbol{\Sigma}_0})$. This completes the proof of Proposition 2. □

B5 Proof of Proposition 3

To simplify the notation, here we always consider $\mathbf{V}=\boldsymbol{\Sigma}_0$. Let $\bar{\mathbf{A}}$ be a $K\times K$ block matrix with the (s,t)-th block $[\mathbb{1}(s=c_1)-\mathbb{1}(s=c_0)][\mathbb{1}(t=c_1)-\mathbb{1}(t=c_0)]\cdot\gamma\boldsymbol{\Sigma}_0^{-1}$. We can verify that

$$\mathrm{Cov}(\hat{\boldsymbol{\xi}}_{\boldsymbol{\Sigma}_0}) = \mathbf{J}'^{-1}_{\boldsymbol{\Sigma}_0} - \begin{bmatrix}\boldsymbol{\Gamma}' & 0\\ 0 & 0\end{bmatrix} = \left(\mathbf{J}_{\boldsymbol{\xi\xi}} + \begin{bmatrix}0 & 0\\ 0 & \bar{\mathbf{A}}\end{bmatrix}\right)^{-1} - \begin{bmatrix}\boldsymbol{\Gamma}' & 0\\ 0 & 0\end{bmatrix} \leq \mathbf{J}^{-1}_{\boldsymbol{\xi\xi}} - \begin{bmatrix}\boldsymbol{\Gamma}' & 0\\ 0 & 0\end{bmatrix},$$

where the right-hand-side is the variance of the internal based MLE $\hat{\boldsymbol{\xi}}_{mle}$. The last inequality holds due to $\bar{\mathbf{A}} \geq 0$.

To show that $\hat{\boldsymbol{\xi}}_{\boldsymbol{\Sigma}_0}$ is asymptotically more efficient than the restricted MLE, we first derive the covariance of $\hat{\boldsymbol{\xi}}_{rmle}$. We can rewrite the problem (16) as the following Lagrange function

$$L(\boldsymbol{\lambda},\boldsymbol{\xi}) = -\sum_{i=1}^{n}\log\{1+\sum_{k=1}^{K}\rho_k\Delta_k(\boldsymbol{X}_i;\boldsymbol{\xi})\} + \sum_{i=1}^{n}\sum_{k=1}^{K}\mathbb{1}(Y_i=k)\cdot\log\{\Delta_k(\boldsymbol{X}_i;\boldsymbol{\xi})\} - n\boldsymbol{\lambda}^\top(\boldsymbol{\theta}_{c_1}-\boldsymbol{\theta}_{c_0}-\tilde{\boldsymbol{\beta}}),$$

where $\boldsymbol{\lambda}$ is the Lagrange multiplier. Denote $\boldsymbol{\psi} = (\boldsymbol{\lambda}^\top, \boldsymbol{\xi}^\top)^\top$ and its score function $\ell_{\boldsymbol{\psi}} = \partial L/\partial\boldsymbol{\psi}$. Note that the true value of $\boldsymbol{\psi}^*$ is $(\mathbf{0}, \boldsymbol{\xi}^*)$. Based on the Taylor's expansion and similar arguments in the proof of Proposition 1, we have

$$\mathbf{0} = n^{-1}\ell_{\boldsymbol{\psi}}(\hat{\boldsymbol{\psi}}_{rmle}) = n^{-1}\ell_{\boldsymbol{\psi}}(\boldsymbol{\psi}^*) + n^{-1}\ell_{\boldsymbol{\psi}\boldsymbol{\psi}}(\boldsymbol{\psi}^*)(\hat{\boldsymbol{\psi}}_{rmle}-\boldsymbol{\psi}^*) + n^{-1}\ell_{\boldsymbol{\psi}\boldsymbol{\beta}}(\boldsymbol{\psi}^*)(\tilde{\boldsymbol{\beta}}-\boldsymbol{\beta}^*) + o_p(n^{-1/2}).$$

Note that $\mathbb{E}[-n^{-1}\ell_{\boldsymbol{\psi}\boldsymbol{\psi}}(\boldsymbol{\psi}^*)] = \begin{bmatrix} 0 & \mathbf{J}_{\boldsymbol{\lambda}\boldsymbol{\xi}} \\ \mathbf{J}_{\boldsymbol{\xi}\boldsymbol{\lambda}} & \mathbf{J}_{\boldsymbol{\xi}\boldsymbol{\xi}} \end{bmatrix} \triangleq \mathbf{J}_{\boldsymbol{\psi}\boldsymbol{\psi}}$, $\mathbb{E}[n^{-1}\ell_{\boldsymbol{\psi}\boldsymbol{\beta}}(\boldsymbol{\psi}^*)] = \begin{bmatrix} \mathbf{I} \\ 0 \end{bmatrix} \triangleq \mathbf{J}_{\boldsymbol{\psi}\boldsymbol{\beta}}$ and $\mathrm{Cov}\{n^{-1/2}\ell_{\boldsymbol{\psi}}(\boldsymbol{\psi}^*)\} = \begin{bmatrix} j_{\boldsymbol{\beta}\boldsymbol{\beta}}^{-1} & 0 \\ 0 & \mathbf{I}_{\boldsymbol{\xi}\boldsymbol{\xi}} \end{bmatrix} \triangleq \mathbf{I}_{\boldsymbol{\psi}\boldsymbol{\psi}}$. Thus we have

$$n^{1/2}(\hat{\boldsymbol{\psi}}_{rmle}-\boldsymbol{\psi}^*) \xrightarrow{d} \mathcal{N}(\mathbf{0}, \mathbf{J}_{\boldsymbol{\psi}\boldsymbol{\psi}}^{-1}\mathbf{I}_{\boldsymbol{\psi}\boldsymbol{\psi}}\mathbf{J}_{\boldsymbol{\psi}\boldsymbol{\psi}}^{-1} + \mathbf{J}_{\boldsymbol{\psi}\boldsymbol{\psi}}^{-1}\mathbf{J}_{\boldsymbol{\psi}\boldsymbol{\beta}}j_{\boldsymbol{\beta}\boldsymbol{\beta}}^{-1}\mathbf{J}_{\boldsymbol{\beta}\boldsymbol{\psi}}\mathbf{J}_{\boldsymbol{\psi}\boldsymbol{\psi}}^{-1}).$$

Since $\mathbf{J}_{\boldsymbol{\xi}\boldsymbol{\xi}}$ is positive definite and $\mathbf{J}_{\boldsymbol{\lambda}\boldsymbol{\xi}}$ is finite with full row rank, we can define $\mathbf{A}_1 = \left(\mathbf{J}_{\boldsymbol{\lambda}\boldsymbol{\xi}}\mathbf{J}_{\boldsymbol{\xi}\boldsymbol{\xi}}^{-1}\mathbf{J}_{\boldsymbol{\xi}\boldsymbol{\lambda}}\right)^{-1}$ and $\mathbf{A}_2 = \mathbf{J}_{\boldsymbol{\xi}\boldsymbol{\xi}}^{-1} - \mathbf{J}_{\boldsymbol{\xi}\boldsymbol{\xi}}^{-1}\mathbf{J}_{\boldsymbol{\xi}\boldsymbol{\lambda}}\mathbf{A}_1\mathbf{J}_{\boldsymbol{\lambda}\boldsymbol{\xi}}\mathbf{J}_{\boldsymbol{\xi}\boldsymbol{\xi}}^{-1}$. Thus, we have $\mathbf{J}_{\boldsymbol{\psi}\boldsymbol{\psi}}^{-1} = \begin{bmatrix} -\mathbf{A}_1 & \mathbf{A}_1\mathbf{J}_{\boldsymbol{\lambda}\boldsymbol{\xi}}\mathbf{J}_{\boldsymbol{\xi}\boldsymbol{\xi}}^{-1} \\ \mathbf{J}_{\boldsymbol{\xi}\boldsymbol{\xi}}^{-1}\mathbf{J}_{\boldsymbol{\xi}\boldsymbol{\lambda}}\mathbf{A}_1 & \mathbf{A}_2 \end{bmatrix}$. It is clear that

$$\mathbf{J}_{\boldsymbol{\xi}\boldsymbol{\xi}}^{-1}\mathbf{I}_{\boldsymbol{\xi}\boldsymbol{\xi}}\mathbf{J}_{\boldsymbol{\xi}\boldsymbol{\xi}}^{-1} = \mathbf{J}_{\boldsymbol{\xi}\boldsymbol{\xi}}^{-1} - \begin{bmatrix} \boldsymbol{\Gamma}' & 0 \\ 0 & 0 \end{bmatrix} \triangleq \mathbf{J}_{\boldsymbol{\xi}\boldsymbol{\xi}}^{-1} - \mathbf{B}.$$

Since $\mathbf{J}_{\boldsymbol{\lambda}\boldsymbol{\xi}} = (0, j_{\boldsymbol{\lambda}\boldsymbol{\theta}})$, we know $\mathbf{J}_{\boldsymbol{\lambda}\boldsymbol{\xi}}\mathbf{B} = \begin{bmatrix} 0 & 0 \end{bmatrix}$ and $\mathbf{B}\mathbf{J}_{\boldsymbol{\xi}\boldsymbol{\lambda}} = \begin{bmatrix} 0 \\ 0 \end{bmatrix}$. Specifically, we have

$$\begin{aligned}
\mathbf{A}_2\mathbf{I}_{\boldsymbol{\xi}\boldsymbol{\xi}}\mathbf{A}_2 =& \mathbf{J}_{\boldsymbol{\xi}\boldsymbol{\xi}}^{-1}\mathbf{I}_{\boldsymbol{\xi}\boldsymbol{\xi}}\mathbf{J}_{\boldsymbol{\xi}\boldsymbol{\xi}}^{-1} - \mathbf{J}_{\boldsymbol{\xi}\boldsymbol{\xi}}^{-1}\mathbf{J}_{\boldsymbol{\xi}\boldsymbol{\lambda}}\mathbf{A}_1\mathbf{J}_{\boldsymbol{\lambda}\boldsymbol{\xi}}\mathbf{J}_{\boldsymbol{\xi}\boldsymbol{\xi}}^{-1}\mathbf{I}_{\boldsymbol{\xi}\boldsymbol{\xi}}\mathbf{J}_{\boldsymbol{\xi}\boldsymbol{\xi}}^{-1} - \mathbf{J}_{\boldsymbol{\xi}\boldsymbol{\xi}}^{-1}\mathbf{I}_{\boldsymbol{\xi}\boldsymbol{\xi}}\mathbf{J}_{\boldsymbol{\xi}\boldsymbol{\xi}}^{-1}\mathbf{J}_{\boldsymbol{\xi}\boldsymbol{\lambda}}\mathbf{A}_1\mathbf{J}_{\boldsymbol{\lambda}\boldsymbol{\xi}}\mathbf{J}_{\boldsymbol{\xi}\boldsymbol{\xi}}^{-1} \\
&+ \mathbf{J}_{\boldsymbol{\xi}\boldsymbol{\xi}}^{-1}\mathbf{J}_{\boldsymbol{\xi}\boldsymbol{\lambda}}\mathbf{A}_1\mathbf{J}_{\boldsymbol{\lambda}\boldsymbol{\xi}}\mathbf{J}_{\boldsymbol{\xi}\boldsymbol{\xi}}^{-1}\mathbf{I}_{\boldsymbol{\xi}\boldsymbol{\xi}}\mathbf{J}_{\boldsymbol{\xi}\boldsymbol{\xi}}^{-1}\mathbf{J}_{\boldsymbol{\xi}\boldsymbol{\lambda}}\mathbf{A}_1\mathbf{J}_{\boldsymbol{\lambda}\boldsymbol{\xi}}\mathbf{J}_{\boldsymbol{\xi}\boldsymbol{\xi}}^{-1} \\
=& (\mathbf{J}_{\boldsymbol{\xi}\boldsymbol{\xi}}^{-1} - \mathbf{B}) - \mathbf{J}_{\boldsymbol{\xi}\boldsymbol{\xi}}^{-1}\mathbf{J}_{\boldsymbol{\xi}\boldsymbol{\lambda}}\mathbf{A}_1\mathbf{J}_{\boldsymbol{\lambda}\boldsymbol{\xi}}(\mathbf{J}_{\boldsymbol{\xi}\boldsymbol{\xi}}^{-1} - \mathbf{B}) - (\mathbf{J}_{\boldsymbol{\xi}\boldsymbol{\xi}}^{-1} - \mathbf{B})\mathbf{J}_{\boldsymbol{\xi}\boldsymbol{\lambda}}\mathbf{A}_1\mathbf{J}_{\boldsymbol{\lambda}\boldsymbol{\xi}}\mathbf{J}_{\boldsymbol{\xi}\boldsymbol{\xi}}^{-1} \\
&+ \mathbf{J}_{\boldsymbol{\xi}\boldsymbol{\xi}}^{-1}\mathbf{J}_{\boldsymbol{\xi}\boldsymbol{\lambda}}\mathbf{A}_1\mathbf{J}_{\boldsymbol{\lambda}\boldsymbol{\xi}}(\mathbf{J}_{\boldsymbol{\xi}\boldsymbol{\xi}}^{-1} - \mathbf{B})\mathbf{J}_{\boldsymbol{\xi}\boldsymbol{\lambda}}\mathbf{A}_1\mathbf{J}_{\boldsymbol{\lambda}\boldsymbol{\xi}}\mathbf{J}_{\boldsymbol{\xi}\boldsymbol{\xi}}^{-1} \\
=& \mathbf{J}_{\boldsymbol{\xi}\boldsymbol{\xi}}^{-1} - \mathbf{B} - 2\mathbf{J}_{\boldsymbol{\xi}\boldsymbol{\xi}}^{-1}\mathbf{J}_{\boldsymbol{\xi}\boldsymbol{\lambda}}\mathbf{A}_1\mathbf{J}_{\boldsymbol{\lambda}\boldsymbol{\xi}}\mathbf{J}_{\boldsymbol{\xi}\boldsymbol{\xi}}^{-1} + \mathbf{J}_{\boldsymbol{\xi}\boldsymbol{\xi}}^{-1}\mathbf{J}_{\boldsymbol{\xi}\boldsymbol{\lambda}}\mathbf{A}_1\mathbf{J}_{\boldsymbol{\lambda}\boldsymbol{\xi}}\mathbf{J}_{\boldsymbol{\xi}\boldsymbol{\xi}}^{-1}\mathbf{J}_{\boldsymbol{\xi}\boldsymbol{\lambda}}\mathbf{A}_1\mathbf{J}_{\boldsymbol{\lambda}\boldsymbol{\xi}}\mathbf{J}_{\boldsymbol{\xi}\boldsymbol{\xi}}^{-1} \\
=& \mathbf{J}_{\boldsymbol{\xi}\boldsymbol{\xi}}^{-1} - \mathbf{J}_{\boldsymbol{\xi}\boldsymbol{\xi}}^{-1}\mathbf{J}_{\boldsymbol{\xi}\boldsymbol{\lambda}}\mathbf{A}_1\mathbf{J}_{\boldsymbol{\lambda}\boldsymbol{\xi}}\mathbf{J}_{\boldsymbol{\xi}\boldsymbol{\xi}}^{-1} - \mathbf{B}.
\end{aligned}$$

This result implies that

$$
\begin{aligned}
\{\mathbf{J}_{\psi\psi}^{-1}\mathbf{I}_{\psi\psi}\mathbf{J}_{\psi\psi}^{-1}\}_{\xi\xi} &= \left\{ \begin{bmatrix} -\mathbf{A}_1 & \mathbf{A}_1\mathbf{J}_{\lambda\xi}\mathbf{J}_{\xi\xi}^{-1} \\ \mathbf{J}_{\xi\xi}^{-1}\mathbf{J}_{\xi\lambda}\mathbf{A}_1 & \mathbf{A}_2 \end{bmatrix} \begin{bmatrix} j_{\beta\beta}^{-1} & 0 \\ 0 & \mathbf{I}_{\xi\xi} \end{bmatrix} \begin{bmatrix} -\mathbf{A}_1 & \mathbf{A}_1\mathbf{J}_{\lambda\xi}\mathbf{J}_{\xi\xi}^{-1} \\ \mathbf{J}_{\xi\xi}^{-1}\mathbf{J}_{\xi\lambda}\mathbf{A}_1 & \mathbf{A}_2 \end{bmatrix} \right\}_{\xi\xi} \\
&= \mathbf{J}_{\xi\xi}^{-1}\mathbf{J}_{\xi\lambda}\mathbf{A}_1 j_{\beta\beta}^{-1}\mathbf{A}_1\mathbf{J}_{\lambda\xi}\mathbf{J}_{\xi\xi}^{-1} + \mathbf{A}_2\mathbf{I}_{\xi\xi}\mathbf{A}_2 \\
&= \mathbf{J}_{\xi\xi}^{-1} + \mathbf{J}_{\xi\xi}^{-1}\mathbf{J}_{\xi\lambda}\mathbf{A}_1 j_{\beta\beta}^{-1}\mathbf{A}_1\mathbf{J}_{\lambda\xi}\mathbf{J}_{\xi\xi}^{-1} - \mathbf{J}_{\xi\xi}^{-1}\mathbf{J}_{\xi\lambda}\mathbf{A}_1\mathbf{J}_{\lambda\xi}\mathbf{J}_{\xi\xi}^{-1} - \mathbf{B}.
\end{aligned}
$$

Similarly, we have

$$
\begin{aligned}
\{\mathbf{J}_{\psi\psi}^{-1}\mathbf{J}_{\psi\beta} j_{\beta\beta}^{-1}\mathbf{J}_{\beta\psi}\mathbf{J}_{\psi\psi}^{-1}\}_{\xi\xi} &= \left\{ \begin{bmatrix} -\mathbf{A}_1 & \mathbf{A}_1\mathbf{J}_{\lambda\xi}\mathbf{J}_{\xi\xi}^{-1} \\ \mathbf{J}_{\xi\xi}^{-1}\mathbf{J}_{\xi\lambda}\mathbf{A}_1 & \mathbf{A}_2 \end{bmatrix} \begin{bmatrix} \mathbf{I} \\ 0 \end{bmatrix} j_{\beta\beta}^{-1} \begin{bmatrix} \mathbf{I} & 0 \end{bmatrix} \begin{bmatrix} -\mathbf{A}_1 & \mathbf{A}_1\mathbf{J}_{\lambda\xi}\mathbf{J}_{\xi\xi}^{-1} \\ \mathbf{J}_{\xi\xi}^{-1}\mathbf{J}_{\xi\lambda}\mathbf{A}_1 & \mathbf{A}_2 \end{bmatrix} \right\}_{\xi\xi} \\
&= \left\{ \begin{bmatrix} -\mathbf{A}_1 & \mathbf{A}_1\mathbf{J}_{\lambda\xi}\mathbf{J}_{\xi\xi}^{-1} \\ \mathbf{J}_{\xi\xi}^{-1}\mathbf{J}_{\xi\lambda}\mathbf{A}_1 & \mathbf{A}_2 \end{bmatrix} \begin{bmatrix} j_{\beta\beta}^{-1} & 0 \\ 0 & 0 \end{bmatrix} \begin{bmatrix} -\mathbf{A}_1 & \mathbf{A}_1\mathbf{J}_{\lambda\xi}\mathbf{J}_{\xi\xi}^{-1} \\ \mathbf{J}_{\xi\xi}^{-1}\mathbf{J}_{\xi\lambda}\mathbf{A}_1 & \mathbf{A}_2 \end{bmatrix} \right\}_{\xi\xi} \\
&= \mathbf{J}_{\xi\xi}^{-1}\mathbf{J}_{\xi\lambda}\mathbf{A}_1 j_{\beta\beta}^{-1}\mathbf{A}_1\mathbf{J}_{\lambda\xi}\mathbf{J}_{\xi\xi}^{-1}.
\end{aligned}
$$

This result implies that

$$
\begin{aligned}
\mathrm{Cov}(\hat{\boldsymbol{\xi}}_{rmle}) &= \mathbf{J}_{\xi\xi}^{-1} + 2\mathbf{J}_{\xi\xi}^{-1}\mathbf{J}_{\xi\lambda}\mathbf{A}_1 j_{\beta\beta}^{-1}\mathbf{A}_1\mathbf{J}_{\lambda\xi}\mathbf{J}_{\xi\xi}^{-1} - \mathbf{J}_{\xi\xi}^{-1}\mathbf{J}_{\xi\lambda}\mathbf{A}_1\mathbf{J}_{\lambda\xi}\mathbf{J}_{\xi\xi}^{-1} - \mathbf{B} \\
&= \mathbf{J}_{\xi\xi}^{-1} + \mathbf{J}_{\xi\xi}^{-1}\mathbf{J}_{\xi\lambda}(2\mathbf{A}_1 j_{\beta\beta}^{-1}\mathbf{A}_1 - \mathbf{A}_1)\mathbf{J}_{\lambda\xi}\mathbf{J}_{\xi\xi}^{-1} - \mathbf{B}.
\end{aligned}
$$

We also know that

$$
\begin{aligned}
\mathrm{Cov}(\hat{\boldsymbol{\xi}}_{\boldsymbol{\Sigma}_0}) &= \left(\mathbf{J}_{\xi\xi} + \begin{bmatrix} 0 & 0 \\ 0 & \bar{\mathbf{A}} \end{bmatrix} \right)^{-1} - \mathbf{B} = (\mathbf{J}_{\xi\xi} + \mathbf{J}_{\xi\lambda} j_{\beta\beta}\mathbf{J}_{\lambda\xi})^{-1} - \mathbf{B} \\
&= \mathbf{J}_{\xi\xi}^{-1} - \mathbf{J}_{\xi\xi}^{-1}\mathbf{J}_{\xi\lambda}(j_{\beta\beta}^{-1} + \mathbf{J}_{\lambda\xi}\mathbf{J}_{\xi\xi}^{-1}\mathbf{J}_{\xi\lambda})^{-1}\mathbf{J}_{\lambda\xi}\mathbf{J}_{\xi\xi}^{-1} - \mathbf{B} \\
&= \mathbf{J}_{\xi\xi}^{-1} + \mathbf{J}_{\xi\xi}^{-1}\mathbf{J}_{\xi\lambda}[-(j_{\beta\beta}^{-1} + \mathbf{A}_1^{-1})^{-1}]\mathbf{J}_{\lambda\xi}\mathbf{J}_{\xi\xi}^{-1} - \mathbf{B},
\end{aligned}
$$

where the Woodbury matrix identity, $(A+UCV)^{-1} = A^{-1} - A^{-1}U(C^{-1} + VA^{-1}U)^{-1}VA^{-1}$, is used in the second line. Using the Woodbury matrix identity again, then

$$
\begin{aligned}
-(j_{\beta\beta}^{-1} + \mathbf{A}_1^{-1})^{-1} &= -[\mathbf{A}_1 - \mathbf{A}_1(\mathbf{A}_1 + j_{\beta\beta})^{-1}\mathbf{A}_1] \\
&= \mathbf{A}_1(\mathbf{A}_1 + j_{\beta\beta})^{-1}\mathbf{A}_1 - \mathbf{A}_1 \\
&\leq \mathbf{A}_1\left(\frac{1}{2} j_{\beta\beta}\right)^{-1}\mathbf{A}_1 - \mathbf{A}_1 = 2\mathbf{A}_1 j_{\beta\beta}^{-1}\mathbf{A}_1 - \mathbf{A}_1.
\end{aligned}
$$

The last inequality holds due to $\mathbf{A}_1 + j_{\beta\beta} \geq \frac{1}{2} j_{\beta\beta}$ and $\mathbf{A}_1, j_{\beta\beta} \geq 0$. It is straightforward to verify that $\mathrm{Cov}(\hat{\boldsymbol{\xi}}_{\boldsymbol{\Sigma}_0}) \leq \mathrm{Cov}(\hat{\boldsymbol{\xi}}_{rmle})$. This completes the proof of Proposition 1. □

B6 Proof of Proposition 4

As mentioned in Section 2.7, we have the following Proposition.

Proposition 4. *The asymptotic efficiency of* $\hat{\boldsymbol{\xi}}$ *cannot decrease when additional summary information is added, i.e.,* $\mathrm{Cov}(\hat{\boldsymbol{\xi}}_{\boldsymbol{\Sigma}_{11}}) \geq \mathrm{Cov}(\hat{\boldsymbol{\xi}}_{\boldsymbol{\Sigma}})$.

Proof: First, we need some new notations for convenience of expression. Let $\boldsymbol{\gamma}_i = (\boldsymbol{\alpha}_i^\top, \boldsymbol{\beta}_i^\top)^\top$ be the collection of the $i-$th external parameters, and $\boldsymbol{\gamma} = (\boldsymbol{\alpha}_1^\top, \boldsymbol{\alpha}_2^\top, \boldsymbol{\beta}_1^\top, \boldsymbol{\beta}_2^\top)^\top$ be the collection of the joint external parameters. Denote $\boldsymbol{s}_1 = (\boldsymbol{\lambda}^\top, \boldsymbol{v}_1^\top)^\top$ as the collection of Lagrange multipliers for the estimate $\hat{\boldsymbol{\xi}}_{\boldsymbol{\Sigma}_{11}}$, and $\boldsymbol{s}_2 = (\boldsymbol{s}_1^\top, \boldsymbol{v}_2^\top)^\top = (\boldsymbol{\lambda}^\top, \boldsymbol{v}^\top)^\top$ as the collection of Lagrange multipliers for the estimate $\hat{\boldsymbol{\xi}}_{\boldsymbol{\Sigma}}$. Furthermore, we denote $\mathbf{M}_1 = (j_{\boldsymbol{v}_1\boldsymbol{v}_1} - j_{\boldsymbol{v}_1\boldsymbol{\lambda}} j_{\boldsymbol{\lambda}\boldsymbol{\lambda}}^{-1} j_{\boldsymbol{\lambda}\boldsymbol{v}_1})^{-1}$ and

$$\mathbf{M}_2 = (j_{\boldsymbol{v}\boldsymbol{v}} - j_{\boldsymbol{v}\boldsymbol{\lambda}} j_{\boldsymbol{\lambda}\boldsymbol{\lambda}}^{-1} j_{\boldsymbol{\lambda}\boldsymbol{v}})^{-1} = \left(\begin{bmatrix} j_{\boldsymbol{v}_1\boldsymbol{v}_1} - j_{\boldsymbol{v}_1\boldsymbol{\lambda}} j_{\boldsymbol{\lambda}\boldsymbol{\lambda}}^{-1} j_{\boldsymbol{\lambda}\boldsymbol{v}_1} & j_{\boldsymbol{v}_1\boldsymbol{v}_2} - j_{\boldsymbol{v}_1\boldsymbol{\lambda}} j_{\boldsymbol{\lambda}\boldsymbol{\lambda}}^{-1} j_{\boldsymbol{\lambda}\boldsymbol{v}_2} \\ j_{\boldsymbol{v}_2\boldsymbol{v}_1} - j_{\boldsymbol{v}_2\boldsymbol{\lambda}} j_{\boldsymbol{\lambda}\boldsymbol{\lambda}}^{-1} j_{\boldsymbol{\lambda}\boldsymbol{v}_1} & j_{\boldsymbol{v}_2\boldsymbol{v}_2} - j_{\boldsymbol{v}_2\boldsymbol{\lambda}} j_{\boldsymbol{\lambda}\boldsymbol{\lambda}}^{-1} j_{\boldsymbol{\lambda}\boldsymbol{v}_2} \end{bmatrix} \right)^{-1}.$$

From the proof of Proposition 1, we have

$$\mathrm{Cov}(\hat{\boldsymbol{\xi}}_{\boldsymbol{\Sigma}_{11}}) = \left(\mathbf{J}_{\xi\xi} + \mathbf{J}_{\xi \boldsymbol{s}_1} \left(-\mathbf{J}_{\boldsymbol{s}_1\boldsymbol{s}_1}^{-1} - \mathbf{J}_{\boldsymbol{s}_1\boldsymbol{s}_1}^{-1} \begin{bmatrix} 0 & 0 \\ 0 & \left(-\mathbf{M}_1 + \mathbf{J}_{\boldsymbol{\gamma}_1\boldsymbol{v}_1}^{-1} \begin{bmatrix} 0 & 0 \\ 0 & \gamma \boldsymbol{\Sigma}_{11}^{-1} \end{bmatrix} \mathbf{J}_{\boldsymbol{v}_1\boldsymbol{\gamma}_1}^{-1} \right)^{-1} \end{bmatrix} \mathbf{J}_{\boldsymbol{s}_1\boldsymbol{s}_1}^{-1} \right) \mathbf{J}_{\boldsymbol{s}_1\xi} \right)^{-1},$$

$$\mathrm{Cov}(\hat{\boldsymbol{\xi}}_{\boldsymbol{\Sigma}}) = \left(\mathbf{J}_{\xi\xi} + \mathbf{J}_{\xi \boldsymbol{s}_2} \left(-\mathbf{J}_{\boldsymbol{s}_2\boldsymbol{s}_2}^{-1} - \mathbf{J}_{\boldsymbol{s}_2\boldsymbol{s}_2}^{-1} \begin{bmatrix} 0 & 0 \\ 0 & \left(-\mathbf{M}_2 + \mathbf{J}_{\boldsymbol{\gamma}\boldsymbol{v}}^{-1} \begin{bmatrix} 0 & 0 \\ 0 & \gamma \boldsymbol{\Sigma}^{-1} \end{bmatrix} \mathbf{J}_{\boldsymbol{v}\boldsymbol{\gamma}}^{-1} \right)^{-1} \end{bmatrix} \mathbf{J}_{\boldsymbol{s}_2\boldsymbol{s}_2}^{-1} \right) \mathbf{J}_{\boldsymbol{s}_2\xi} \right)^{-1}.$$

Let

$$\mathrm{U}_1 = -\mathbf{J}_{\boldsymbol{s}_1\boldsymbol{s}_1}^{-1} - \mathbf{J}_{\boldsymbol{s}_1\boldsymbol{s}_1}^{-1} \begin{bmatrix} 0 & 0 \\ 0 & \left(-\mathbf{M}_1 + \mathbf{J}_{\boldsymbol{\gamma}_1\boldsymbol{v}_1}^{-1} \begin{bmatrix} 0 & 0 \\ 0 & \gamma \boldsymbol{\Sigma}_{11}^{-1} \end{bmatrix} \mathbf{J}_{\boldsymbol{v}_1\boldsymbol{\gamma}_1}^{-1} \right)^{-1} \end{bmatrix} \mathbf{J}_{\boldsymbol{s}_1\boldsymbol{s}_1}^{-1} \geq \mathbf{0},$$

$$\mathrm{U}_2 = -\mathbf{J}_{\boldsymbol{s}_2\boldsymbol{s}_2}^{-1} - \mathbf{J}_{\boldsymbol{s}_2\boldsymbol{s}_2}^{-1} \begin{bmatrix} 0 & 0 \\ 0 & \left(-\mathbf{M}_2 + \mathbf{J}_{\boldsymbol{\gamma}\boldsymbol{v}}^{-1} \begin{bmatrix} 0 & 0 \\ 0 & \gamma \boldsymbol{\Sigma}^{-1} \end{bmatrix} \mathbf{J}_{\boldsymbol{v}\boldsymbol{\gamma}}^{-1} \right)^{-1} \end{bmatrix} \mathbf{J}_{\boldsymbol{s}_2\boldsymbol{s}_2}^{-1} \geq \mathbf{0}.$$

Therefore, we have $\mathrm{Cov}(\hat{\boldsymbol{\xi}}_{\boldsymbol{\Sigma}_{11}}) = (\mathbf{J}_{\xi\xi} + \mathbf{J}_{\xi\boldsymbol{s}_1} \mathrm{U}_1 \mathbf{J}_{\boldsymbol{s}_1\xi})^{-1}$ and $\mathrm{Cov}(\hat{\boldsymbol{\xi}}_{\boldsymbol{\Sigma}}) = (\mathbf{J}_{\xi\xi} + \mathbf{J}_{\xi\boldsymbol{s}_2} \mathrm{U}_2 \mathbf{J}_{\boldsymbol{s}_2\xi})^{-1}$.

Using some fundamental matrix algebra, we have the facts that $\begin{bmatrix} \boldsymbol{\Sigma}_{11}^{-1} & 0 \\ 0 & 0 \end{bmatrix} \leq \boldsymbol{\Sigma}^{-1} = \begin{bmatrix} \boldsymbol{\Sigma}_{11} & \boldsymbol{\Sigma}_{12} \\ \boldsymbol{\Sigma}_{21} & \boldsymbol{\Sigma}_{22} \end{bmatrix}^{-1}$

and

$$\mathbf{J}_{\boldsymbol{s}_2\boldsymbol{s}_2} = \begin{bmatrix} j_{\boldsymbol{\lambda}\boldsymbol{\lambda}} & j_{\boldsymbol{\lambda}\boldsymbol{\nu}_1} & j_{\boldsymbol{\lambda}\boldsymbol{\nu}_2} \\ j_{\boldsymbol{\nu}_1\boldsymbol{\lambda}} & j_{\boldsymbol{\nu}_1\boldsymbol{\nu}_1} & j_{\boldsymbol{\nu}_1\boldsymbol{\nu}_2} \\ j_{\boldsymbol{\nu}_2\boldsymbol{\lambda}} & j_{\boldsymbol{\nu}_2\boldsymbol{\nu}_1} & j_{\boldsymbol{\nu}_2\boldsymbol{\nu}_2} \end{bmatrix} = \begin{bmatrix} \mathbf{J}_{\boldsymbol{s}_1\boldsymbol{s}_1} & \mathbf{J}_{\boldsymbol{s}_1\boldsymbol{\nu}_2} \\ \mathbf{J}_{\boldsymbol{\nu}_2\boldsymbol{s}_1} & j_{\boldsymbol{\nu}_2\boldsymbol{\nu}_2} \end{bmatrix} = \begin{bmatrix} j_{\boldsymbol{\lambda}\boldsymbol{\lambda}} & \mathbf{J}_{\boldsymbol{\lambda}\boldsymbol{\nu}} \\ \mathbf{J}_{\boldsymbol{\nu}\boldsymbol{\lambda}} & \mathbf{J}_{\boldsymbol{\nu}\boldsymbol{\nu}} \end{bmatrix},$$

$$\mathbf{J}^{-1}_{\boldsymbol{s}_1\boldsymbol{s}_1} = \begin{bmatrix} j^{-1}_{\boldsymbol{\lambda}\boldsymbol{\lambda}} + j^{-1}_{\boldsymbol{\lambda}\boldsymbol{\lambda}} j_{\boldsymbol{\lambda}\boldsymbol{\nu}_1}\mathbf{M}_1 j_{\boldsymbol{\nu}\boldsymbol{\lambda}} j^{-1}_{\boldsymbol{\lambda}\boldsymbol{\lambda}} & -j^{-1}_{\boldsymbol{\lambda}\boldsymbol{\lambda}} j_{\boldsymbol{\lambda}\boldsymbol{\nu}}\mathbf{M}_1 \\ -\mathbf{M}_1 j_{\boldsymbol{\nu}_1\boldsymbol{\lambda}} j^{-1}_{\boldsymbol{\lambda}\boldsymbol{\lambda}} & \mathbf{M}_1 \end{bmatrix}$$

$$\mathbf{J}^{-1}_{\boldsymbol{s}_2\boldsymbol{s}_2} = \begin{bmatrix} j^{-1}_{\boldsymbol{\lambda}\boldsymbol{\lambda}} + j^{-1}_{\boldsymbol{\lambda}\boldsymbol{\lambda}} \mathbf{J}_{\boldsymbol{\lambda}\boldsymbol{\nu}}\mathbf{M}_2 \mathbf{J}_{\boldsymbol{\nu}\boldsymbol{\lambda}} j^{-1}_{\boldsymbol{\lambda}\boldsymbol{\lambda}} & -j^{-1}_{\boldsymbol{\lambda}\boldsymbol{\lambda}} \mathbf{J}_{\boldsymbol{\lambda}\boldsymbol{\nu}}\mathbf{M}_2 \\ -\mathbf{M}_2 \mathbf{J}_{\boldsymbol{\nu}\boldsymbol{\lambda}} j^{-1}_{\boldsymbol{\lambda}\boldsymbol{\lambda}} & \mathbf{M}_2 \end{bmatrix}.$$

Furthermore, we know

$$\begin{aligned}
\mathbf{U}_2 &\geq -\mathbf{J}^{-1}_{\boldsymbol{s}_2\boldsymbol{s}_2} - \mathbf{J}^{-1}_{\boldsymbol{s}_2\boldsymbol{s}_2} \begin{bmatrix} 0 & 0 \\ 0 & \left(-\mathbf{M}_2 + \mathbf{J}^{-1}_{\boldsymbol{\gamma}\boldsymbol{\nu}} \begin{bmatrix} 0 & 0 & 0 \\ 0 & \gamma\boldsymbol{\Sigma}^{-1}_{11} & 0 \\ 0 & 0 & 0 \end{bmatrix} \mathbf{J}^{-1}_{\boldsymbol{\nu}\boldsymbol{\gamma}} \right)^{-1} \end{bmatrix} \mathbf{J}^{-1}_{\boldsymbol{s}_2\boldsymbol{s}_2} \\
&= -\mathbf{J}^{-1}_{\boldsymbol{s}_2\boldsymbol{s}_2} - \mathbf{J}^{-1}_{\boldsymbol{s}_2\boldsymbol{s}_2} \begin{bmatrix} 0 & 0 \\ 0 & \left(-\mathbf{M}_2 + \begin{bmatrix} \mathbf{J}^{-1}_{\boldsymbol{\gamma}_1\boldsymbol{\nu}_1} \begin{bmatrix} 0 & 0 \\ 0 & \gamma\boldsymbol{\Sigma}^{-1}_{11} \end{bmatrix} \mathbf{J}^{-1}_{\boldsymbol{\nu}_1\boldsymbol{\gamma}_1} & 0 \\ 0 & 0 \end{bmatrix} \right)^{-1} \end{bmatrix} \mathbf{J}^{-1}_{\boldsymbol{s}_2\boldsymbol{s}_2} \\
&= -\mathbf{J}^{-1}_{\boldsymbol{s}_2\boldsymbol{s}_2} - \mathbf{J}^{-1}_{\boldsymbol{s}_2\boldsymbol{s}_2} \begin{bmatrix} 0 & 0 \\ 0 & (\mathbf{C}-\mathbf{M}_2)^{-1} \end{bmatrix} \mathbf{J}^{-1}_{\boldsymbol{s}_2\boldsymbol{s}_2} \\
&= - \begin{bmatrix} j^{-1}_{\boldsymbol{\lambda}\boldsymbol{\lambda}} + j^{-1}_{\boldsymbol{\lambda}\boldsymbol{\lambda}} \mathbf{J}_{\boldsymbol{\lambda}\boldsymbol{\nu}} [\mathbf{C}(\mathbf{C}-\mathbf{M}_2)^{-1}\mathbf{C}-\mathbf{C}] \mathbf{J}_{\boldsymbol{\lambda}\boldsymbol{\nu}} j^{-1}_{\boldsymbol{\lambda}\boldsymbol{\lambda}} & -j^{-1}_{\boldsymbol{\lambda}\boldsymbol{\lambda}} \mathbf{J}_{\boldsymbol{\lambda}\boldsymbol{\nu}} [\mathbf{C}(\mathbf{C}-\mathbf{M}_2)^{-1}\mathbf{C}-\mathbf{C}] \\ -[\mathbf{C}(\mathbf{C}-\mathbf{M}_2)^{-1}\mathbf{C}-\mathbf{C}] \mathbf{J}_{\boldsymbol{\nu}\boldsymbol{\lambda}} j^{-1}_{\boldsymbol{\lambda}\boldsymbol{\lambda}} & \mathbf{C}(\mathbf{C}-\mathbf{M}_2)^{-1}\mathbf{C}-\mathbf{C} \end{bmatrix} \\
&= - \begin{bmatrix} j^{-1}_{\boldsymbol{\lambda}\boldsymbol{\lambda}} + j^{-1}_{\boldsymbol{\lambda}\boldsymbol{\lambda}} j_{\boldsymbol{\lambda}\boldsymbol{\nu}_1} [\mathbf{T}(\mathbf{T}-\mathbf{M}_1)^{-1}\mathbf{T}-\mathbf{T}] j_{\boldsymbol{\lambda}\boldsymbol{\nu}_1} j^{-1}_{\boldsymbol{\lambda}\boldsymbol{\lambda}} & -j^{-1}_{\boldsymbol{\lambda}\boldsymbol{\lambda}} j_{\boldsymbol{\lambda}\boldsymbol{\nu}_1} [\mathbf{T}(\mathbf{T}-\mathbf{M}_2)^{-1}\mathbf{T}-\mathbf{T}] & 0 \\ -[\mathbf{T}(\mathbf{T}-\mathbf{M}_1)^{-1}\mathbf{T}-\mathbf{T}] j_{\boldsymbol{\nu}_1\boldsymbol{\lambda}} j^{-1}_{\boldsymbol{\lambda}\boldsymbol{\lambda}} & \mathbf{T}(\mathbf{T}-\mathbf{M}_1)^{-1}\mathbf{T}-\mathbf{T} & 0 \\ 0 & 0 & 0 \end{bmatrix} \\
&= - \begin{bmatrix} j^{-1}_{\boldsymbol{\lambda}\boldsymbol{\lambda}} + j^{-1}_{\boldsymbol{\lambda}\boldsymbol{\lambda}} j_{\boldsymbol{\lambda}\boldsymbol{\nu}_1} [\mathbf{M}_1(\mathbf{T}-\mathbf{M}_1)^{-1}\mathbf{M}_1+\mathbf{M}_1] j_{\boldsymbol{\lambda}\boldsymbol{\nu}_1} j^{-1}_{\boldsymbol{\lambda}\boldsymbol{\lambda}} & -j^{-1}_{\boldsymbol{\lambda}\boldsymbol{\lambda}} j_{\boldsymbol{\lambda}\boldsymbol{\nu}_1} [\mathbf{M}_1(\mathbf{T}-\mathbf{M}_1)^{-1}\mathbf{M}_1+\mathbf{M}_1] & 0 \\ -[\mathbf{M}_1(\mathbf{T}-\mathbf{M}_1)^{-1}\mathbf{M}_1+\mathbf{M}_1] j_{\boldsymbol{\nu}_1\boldsymbol{\lambda}} j^{-1}_{\boldsymbol{\lambda}\boldsymbol{\lambda}} & \mathbf{M}_1(\mathbf{T}-\mathbf{M}_1)^{-1}\mathbf{M}_1+\mathbf{M}_1 & 0 \\ 0 & 0 & 0 \end{bmatrix} \\
&= \begin{bmatrix} -\mathbf{J}^{-1}_{\boldsymbol{s}_1\boldsymbol{s}_1} - \mathbf{J}^{-1}_{\boldsymbol{s}_1\boldsymbol{s}_1} \begin{bmatrix} 0 & 0 \\ 0 & (\mathbf{T}-\mathbf{M}_1)^{-1} \end{bmatrix} \mathbf{J}^{-1}_{\boldsymbol{s}_1\boldsymbol{s}_1} & 0 \\ 0 & 0 \end{bmatrix} = \begin{bmatrix} \mathbf{U}_1 & 0 \\ 0 & 0 \end{bmatrix}.
\end{aligned}$$

where $\mathbf{C} = \begin{bmatrix} \mathbf{T} & 0 \\ 0 & 0 \end{bmatrix}$ with $\mathbf{T} = \mathbf{J}^{-1}_{\boldsymbol{\gamma}_1\boldsymbol{\nu}_1} \begin{bmatrix} 0 & 0 \\ 0 & \gamma\boldsymbol{\Sigma}^{-1}_{11} \end{bmatrix} \mathbf{J}^{-1}_{\boldsymbol{\nu}_1\boldsymbol{\gamma}_1}$, and $\mathbf{M}_2 + \mathbf{M}_2(\mathbf{C}-\mathbf{M}_2)^{-1}\mathbf{M}_2 = \mathbf{C}(\mathbf{C} -$

$\mathbf{M}_2)^{-1}\mathbf{C} - \mathbf{C}$.

Moreover, we have

$$\mathrm{Cov}(\hat{\boldsymbol{\xi}}_{\boldsymbol{\Sigma}}) = (\mathbf{J}_{\boldsymbol{\xi}\boldsymbol{\xi}} + \mathbf{J}_{\boldsymbol{\xi}\boldsymbol{s}_2}\mathrm{U}_2\mathbf{J}_{\boldsymbol{s}_2\boldsymbol{\xi}})^{-1} \leq \left(\mathbf{J}_{\boldsymbol{\xi}\boldsymbol{\xi}} + \begin{bmatrix}\mathbf{J}_{\boldsymbol{\xi}\boldsymbol{s}_1} & \mathbf{J}_{\boldsymbol{\xi}\boldsymbol{v}_2}\end{bmatrix}\begin{bmatrix}\mathbf{U}_1 & 0 \\ 0 & 0\end{bmatrix}\begin{bmatrix}\mathbf{J}_{\boldsymbol{s}_1\boldsymbol{\xi}} \\ \mathbf{J}_{\boldsymbol{v}_2\boldsymbol{\xi}}\end{bmatrix}\right)^{-1}$$
$$= (\mathbf{J}_{\boldsymbol{\xi}\boldsymbol{\xi}} + \mathbf{J}_{\boldsymbol{\xi}\boldsymbol{s}_1}\mathbf{U}_1\mathbf{J}_{\boldsymbol{s}_1\boldsymbol{\xi}})^{-1} = \mathrm{Cov}(\hat{\boldsymbol{\xi}}_{\boldsymbol{\Sigma}_{11}}).$$

Thus, the results are established, and the proof of Proposition 4 is completed. □

References

[1] White HL. Maximum likelihood estimation of misspecified models. Econometrica. 1982;50(1):1–25.

[2] Zhang H, Deng L, Wheeler W, Qin J, Yu K. Integrative analysis of multiple case-control studies. Biometrics. 2022;78(3):1080–1091.
