# Supplementary material for: Improve the model of disease subtype heterogeneity by leveraging external summary data
Source: PLoS Comput Biol. 2023 Jul 12;19(7):e1011236. doi: 10.1371/journal.pcbi.1011236 (PMC10337985; doi:10.1371/journal.pcbi.1011236)
Supplement: S1 Table — All numbers are multiplied by 100. (PDF) [file pcbi.1011236.s002.pdf]

Table S1: Simulation results in situations when summary data is derived from one external study based on case-case (CC) comparison models considering the rare disease and independent markers. All numbers are multiplied by 100.

		MLE_{int}	CC1: Given $\tilde{\beta}_1$			CC2: Given $(\tilde{\beta}_1, \tilde{\beta}_2)$		
			$GIM_{\mathbf{I}}$	$GIM_{\mathbf{V}_\sigma}$	GIM_{opt}	$GIM_{\mathbf{I}}$	$GIM_{\mathbf{V}_\sigma}$	GIM_{opt}
θ_{11}	Bias	1.20	0.70	0.50	0.70	1.31	0.77	0.97
	SE-Emp	16.34	15.69	12.93	12.98	17.21	13.27	13.28
	SE-Est	16.68	15.98	13.43	13.43	17.22	13.76	13.72
	CP	96.05	95.70	95.45	95.40	95.05	95.85	95.75
θ_{12}	Bias	-0.01	0.13	0.08	0.02	-0.04	0.02	-0.06
	SE-Emp	10.56	10.26	9.94	9.94	10.50	9.90	9.90
	SE-Est	10.51	10.40	9.99	9.99	10.65	9.98	9.97
	CP	95.10	95.00	94.85	94.90	95.30	95.00	94.95
θ_{21}	Bias	0.17	0.17	0.17	0.17	-0.13	0.14	0.03
	SE-Emp	10.75	10.75	10.75	10.75	11.21	10.11	10.11
	SE-Est	10.57	10.57	10.57	10.57	10.84	9.79	9.78
	CP	94.60	94.60	94.60	94.60	94.50	94.10	94.20
θ_{22}	Bias	0.78	0.78	0.78	0.78	1.27	0.74	0.90
	SE-Emp	13.08	13.08	13.08	13.08	13.48	11.42	11.40
	SE-Est	12.93	12.93	12.93	12.93	13.46	11.25	11.23
	CP	95.10	95.10	95.10	95.10	95.20	94.35	94.55
