# Supplementary material for: Improve the model of disease subtype heterogeneity by leveraging external summary data
Source: PLoS Comput Biol. 2023 Jul 12;19(7):e1011236. doi: 10.1371/journal.pcbi.1011236 (PMC10337985; doi:10.1371/journal.pcbi.1011236)
Supplement: S2 Table — All numbers are multiplied by 100. (PDF) [file pcbi.1011236.s003.pdf]

Table S2: Simulation results in situations when summary data is derived from one external study based on grouped case-control (GC) models. All numbers are multiplied by 100. GC1/GC2: grouped case-control model with covariates $X_1/\{X_1, X_2\}$.

		MLE_{int}	GC1: Given $\tilde{\beta}_1$			GC2: Given $(\tilde{\beta}_1, \tilde{\beta}_2)$		
			$\text{GIM}_{\mathbf{I}}$	$\text{GIM}_{\mathbf{V}_\sigma}$	GIM_{opt}	$\text{GIM}_{\mathbf{I}}$	$\text{GIM}_{\mathbf{V}_\sigma}$	GIM_{opt}
θ_{11}	Bias	1.20	0.75	1.02	1.04	0.80	1.03	1.05
	SE-Emp	16.34	17.41	14.84	14.85	17.43	14.88	14.89
	SE-Est	16.68	17.82	15.09	15.09	17.80	15.13	15.12
	CP	96.05	95.30	96.00	96.00	95.45	95.90	95.90
θ_{12}	Bias	-0.01	0.01	-0.07	-0.06	0.03	-0.07	-0.05
	SE-Emp	10.56	11.31	9.14	9.14	11.35	9.16	9.15
	SE-Est	10.51	11.54	9.08	9.08	11.56	9.08	9.07
	CP	95.10	95.55	94.65	94.65	95.20	94.55	94.50
θ_{21}	Bias	0.17	0.17	0.17	0.17	0.11	0.13	0.13
	SE-Emp	10.75	10.75	10.75	10.75	11.25	8.87	8.86
	SE-Est	10.57	10.57	10.57	10.57	11.62	8.76	8.75
	CP	94.60	94.60	94.60	94.60	96.00	94.60	94.70
θ_{22}	Bias	0.78	0.78	0.78	0.78	0.52	0.72	0.71
	SE-Emp	13.08	13.08	13.08	13.08	13.11	12.09	12.09
	SE-Est	12.93	12.93	12.93	12.93	13.48	12.08	12.08
	CP	95.10	95.10	95.10	95.10	95.80	95.30	95.20
