# Supplementary material for: Improve the model of disease subtype heterogeneity by leveraging external summary data
Source: PLoS Comput Biol. 2023 Jul 12;19(7):e1011236. doi: 10.1371/journal.pcbi.1011236 (PMC10337985; doi:10.1371/journal.pcbi.1011236)
Supplement: S3 Table — (PDF) [file pcbi.1011236.s004.pdf]

Table S3: Information on the 21 SNPs used in the definition of Hodgkin lymphoma associated polygenic risk score

Locus	SNP	Position (hg19)	Cases	Controls	MAF	Published log(OR)	Variance	Reference
2p16.1	rs1432295	61,066,666	2,057	3,416	0.4	0.1989	1.25E-03	[1]
3p24.1	rs3806624	27,764,623	2,024	1,853	0.464	0.2311	1.06E-03	[2]
3q28	rs4459895	187,954,414	5,314	16,749	0.228	0.2624	9.16E-04	[3]
5q31.1	rs20541	131,995,964	1,200	6,417	0.197	0.3853	4.55E-03	[4]
6p21.31	rs649775	33,684,313	5,325	22,423	0.081	0.2546	1.61E-03	[5]
6p21.32	rs6903608	32,428,285	2,057	3,416	0.321	0.5306	1.27E-03	[1]
6p21.32	rs2281389	33,059,796	582	4,736	0.172	0.4947	4.73E-03	[6]
6p21.33	rs2248462	31,446,796	1,200	6,417	0.783	0.4947	4.46E-03	[4]
6q22.33	rs9482849	128,288,536	5,314	16,749	0.142	0.1823	1.04E-03	[3]
6q23.3	rs7745098	135,415,004	2,024	1,853	0.497	0.1906	1.04E-03	[2]
6q23.3	rs6928977	135,626,348	5,314	16,749	0.593	0.1570	5.95E-04	[3]
6q23.3	rs1002658	137,981,584	5,325	22,423	0.182	0.1740	9.85E-04	[5]
8q24.21	rs2019960	129,192,271	2,057	3,416	0.211	0.2852	1.48E-03	[1]
10p14	rs501764	8,093,034	2,024	1,853	0.184	0.3293	2.77E-03	[2]
10p14	rs3781093	8,101,927	5,314	16,749	0.845	0.2469	1.28E-03	[3]
11q23.1	rs7111520	111,249,611	5,325	22,423	0.676	0.1740	6.63E-04	[5]
13q34	rs112998813	115,059,729	5,314	16,749	0.068	0.3293	3.63E-03	[3]
16p11.2	rs6565176	30,174,926	5,325	22,423	0.461	0.1484	7.31E-04	[5]
16p13.13	rs34972832	11,198,938	5,314	16,749	0.201	0.2151	1.47E-03	[3]
19p13.3	rs1860661	1,650,134	1,281	3,218	0.591	0.2070	1.09E-03	[7]
20q13.12	rs2425752	44,702,120	5,325	22,423	0.273	0.1398	6.20E-04	[5]

References

[1] Enciso-Mora V, Broderick P, Ma Y, Jarrett RF, Hjalgrim H, Hemminki K, et al. A genome-wide association study of Hodgkin's lymphoma identifies new susceptibility loci at 2p16.1 (REL), 8q24.21 and 10p14 (GATA3). Nat Genet. 2010;42(12):1126–1130.

[2] Frampton M, da Silva Filho MI, Broderick P, Thomsen H, Försti A, Vijayakrishnan J, et al. Variation at 3p24.1 and 6q23.3 influences the risk of Hodgkin's lymphoma. Nat Commun. 2013;4(1):2549.

[3] Sud A, Thomsen H, Law PJ, Försti A, Filho MIdS, Holroyd A, et al. Genome-wide association study of classical Hodgkin lymphoma identifies key regulators of disease susceptibility. Nat Commun. 2017;8(1):1892.

[4] Urayama KY, Jarrett RF, Hjalgrim H, Diepstra A, Kamatani Y, Chabrier A, et al. Genome-wide association study of classical Hodgkin lymphoma and Epstein–Barr virus status–defined subgroups. J Natl Cancer Inst. 2012;104(3):240–253.

[5] Sud A, Thomsen H, Orlando G, Foersti A, Law PJ, Broderick P, et al. Genome-wide association study implicates immune dysfunction in the development of Hodgkin lymphoma. Blood. 2018;132(19):2040–2052.

[6] Moutsianas L, Enciso-Mora V, Ma YP, Leslie S, Dilthey A, Broderick P, et al. Multiple Hodgkin lymphoma–associated loci within the HLA region at chromosome 6p21.3. Blood. 2011;118(3):670–674.

[7] Cozen W, Timofeeva MN, Li D, Diepstra A, Hazelett D, Delahaye-Sourdeix M, et al. A meta-analysis of Hodgkin lymphoma reveals 19p13.3 TCF3 as a novel susceptibility locus. Nat Commun. 2014;5(1):3856.
