# Supplementary material for: Improve the model of disease subtype heterogeneity by leveraging external summary data
Source: PLoS Comput Biol. 2023 Jul 12;19(7):e1011236. doi: 10.1371/journal.pcbi.1011236 (PMC10337985; doi:10.1371/journal.pcbi.1011236)
Supplement: S4 Table — Measurement errors are considered at three different levels: none (i.e., w˜i=wi), low (i.e., w˜i∼N(wi,sei2)), and high (i.e., w˜i∼N(wi,16sei2)). Summary data are derived from five external studies with their sample sizes giving in Table 3. All numbers are multiplied by 100. (PDF) [file pcbi.1011236.s005.pdf]

Table S4: Simulation results of type I errors under the null PRS model for the impact of measurement error. Measurement errors are considered at three different levels: none (i.e., $\tilde{w}_i = w_i$), low (i.e., $\tilde{w}_i \sim \mathcal{N}(w_i, \text{se}_i^2)$), and high (i.e., $\tilde{w}_i \sim \mathcal{N}(w_i, 16\text{se}_i^2)$). Summary data are derived from five external studies with their sample sizes giving in Table 3. All numbers are multiplied by 100.

		None		Low		High	
		MLE	GIM$_{opt}$	MLE	GIM$_{opt}$	MLE	GIM$_{opt}$
θ_1	$\alpha = 0.01$	0.0100	0.0120	0.0070	0.0100	0.0060	0.0100
	$\alpha = 0.05$	0.0500	0.0540	0.0475	0.0460	0.0495	0.0460
	$\alpha = 0.1$	0.1045	0.1015	0.1025	0.1020	0.1005	0.1110
θ_2	$\alpha = 0.01$	0.0085	0.0095	0.0110	0.0095	0.0120	0.0110
	$\alpha = 0.05$	0.0495	0.0500	0.0485	0.0560	0.0525	0.0550
	$\alpha = 0.1$	0.1020	0.1040	0.0975	0.1070	0.1035	0.1125
θ_3	$\alpha = 0.01$	0.0090	0.0080	0.0100	0.0080	0.0105	0.0100
	$\alpha = 0.05$	0.0545	0.0550	0.0515	0.0505	0.0495	0.0460
	$\alpha = 0.1$	0.1030	0.1060	0.1045	0.1165	0.0955	0.0990
θ_4	$\alpha = 0.01$	0.0075	0.0075	0.0125	0.0115	0.0095	0.0085
	$\alpha = 0.05$	0.0405	0.0410	0.0475	0.0470	0.0500	0.0495
	$\alpha = 0.1$	0.0875	0.0895	0.0965	0.0930	0.0920	0.0930
