# Supplementary material for: Improve the model of disease subtype heterogeneity by leveraging external summary data
Source: PLoS Comput Biol. 2023 Jul 12;19(7):e1011236. doi: 10.1371/journal.pcbi.1011236 (PMC10337985; doi:10.1371/journal.pcbi.1011236)
Supplement: S5 Table — We consider an internal study with a sample size reduced to 10% of its original size. Summary data are derived from five external studies, with their sample sizes varying from 1, 5 to 10 times the original sizes reported in Table 3. PRS with no or low measurement error is considered. All numbers are multiplied by 100. (PDF) [file pcbi.1011236.s006.pdf]

Table S5: Simulation results on the impact of sample sizes of external studies under the alternative PRS model. We consider an internal study with a sample size reduced to 10% of its original size. Summary data are derived from five external studies, with their sample sizes varying from 1, 5 to 10 times the original sizes reported in Table 3. PRS with no or low measurement error is considered. All numbers are multiplied by 100.

		$1 \times$ sample size		$5 \times$ sample size		$10 \times$ sample size	
		None	Low	None	Low	None	Low
θ_1	Bias	−0.19	−0.07	−0.48	−0.60	−0.05	−0.27
	SE-Emp	4.90	4.92	2.39	2.39	1.76	1.82
	SE-Est	4.96	4.92	2.47	2.45	1.77	1.76
	CP	95.95	95.05	95.85	95.70	95.10	94.45
θ_2	Bias	−0.20	−0.41	−0.40	−0.68	1.42E-03	−0.47
	SE-Emp	5.80	5.70	3.06	3.10	2.18	2.22
	SE-Est	5.71	5.66	3.07	3.04	2.25	2.23
	CP	95.30	94.30	94.30	94.15	95.95	94.65
θ_3	Bias	−0.63	−0.27	−0.43	−0.17	−0.15	−0.09
	SE-Emp	6.32	6.30	3.40	3.32	2.47	2.59
	SE-Est	6.31	6.26	3.42	3.40	2.51	2.49
	CP	95.30	94.80	95.25	95.50	95.40	94.25
θ_4	Bias	−0.36	−0.49	−0.44	−0.57	0.37	−0.57
	SE-Emp	14.49	14.78	14.34	14.61	14.60	14.55
	SE-Est	14.76	14.65	14.66	14.55	14.65	14.52
	CP	95.65	94.75	96.15	95.05	95.35	94.95
