# Supplementary material for: Improve the model of disease subtype heterogeneity by leveraging external summary data
Source: PLoS Comput Biol. 2023 Jul 12;19(7):e1011236. doi: 10.1371/journal.pcbi.1011236 (PMC10337985; doi:10.1371/journal.pcbi.1011236)
Supplement: S6 Table — Summary data are derived from the first 1, 3, or 5 external studies shown in Table 3, with the number of SNPs varying from 21 to 105 by simply stacking the original 21 SNPs. (PDF) [file pcbi.1011236.s007.pdf]

Table S6: Summary of the average computational time (in minutes) over 100 replications under the Null PRS model. Summary data are derived from the first 1, 3, or 5 external studies shown in Table 3, with the number of SNPs varying from 21 to 105 by simply stacking the original 21 SNPs.

	external study 1			external studies 1,2,3			external studies 1–5		
# SNPs	$\text{GIM}_{\mathbf{I}}$	$\text{GIM}_{\mathbf{V}_\sigma}$	GIM_{opt}	$\text{GIM}_{\mathbf{I}}$	$\text{GIM}_{\mathbf{V}_\sigma}$	GIM_{opt}	$\text{GIM}_{\mathbf{I}}$	$\text{GIM}_{\mathbf{V}_\sigma}$	GIM_{opt}
21	0.64	0.62	2.47	1.44	1.45	6.49	2.47	2.51	11.90
42	1.82	1.77	8.26	4.81	4.71	22.15	9.14	8.48	41.86
63	3.72	3.52	17.59	10.37	9.00	46.80	19.63	16.43	86.80
84	6.48	5.92	30.33	17.50	15.10	79.27	31.38	30.24	141.73
105	9.90	8.86	45.90	26.25	24.76	116.39	48.51	45.01	211.11
