# Supplementary material for: Improve the model of disease subtype heterogeneity by leveraging external summary data
Source: PLoS Comput Biol. 2023 Jul 12;19(7):e1011236. doi: 10.1371/journal.pcbi.1011236 (PMC10337985; doi:10.1371/journal.pcbi.1011236)
Supplement: S7 Table — Summary data are derived from the first 1, 3, or 5 external studies shown in Table 3, with the number of SNPs varying from 21 to 105 by simply stacking the original 21 SNPs. (PDF) [file pcbi.1011236.s008.pdf]

Table S7: Summary of the average memory usage (in Gigabyte) over 100 replications under the Null PRS model. Summary data are derived from the first 1, 3, or 5 external studies shown in Table 3, with the number of SNPs varying from 21 to 105 by simply stacking the original 21 SNPs.

	external study 1			external studies 1,2,3			external studies 1–5		
# SNPs	$\text{GIM}_{\mathbf{I}}$	$\text{GIM}_{\mathbf{V}_\sigma}$	GIM_{opt}	$\text{GIM}_{\mathbf{I}}$	$\text{GIM}_{\mathbf{V}_\sigma}$	GIM_{opt}	$\text{GIM}_{\mathbf{I}}$	$\text{GIM}_{\mathbf{V}_\sigma}$	GIM_{opt}
21	0.16	0.17	0.18	0.23	0.24	0.26	0.30	0.30	0.31
42	0.27	0.27	0.29	0.42	0.42	0.43	0.60	0.60	0.61
63	0.38	0.38	0.39	0.64	0.65	0.65	0.92	0.90	0.94
84	0.50	0.50	0.52	0.87	0.85	0.89	1.27	1.23	1.37
105	0.64	0.64	0.66	1.12	1.08	1.15	1.64	1.58	1.83
