# Supplementary material for: Improve the model of disease subtype heterogeneity by leveraging external summary data
Source: PLoS Comput Biol. 2023 Jul 12;19(7):e1011236. doi: 10.1371/journal.pcbi.1011236 (PMC10337985; doi:10.1371/journal.pcbi.1011236)
Supplement: S8 Table — (PDF) [file pcbi.1011236.s009.pdf]

Table S8: Estimated PRS effects on four NHL subtypes using 1/3 of the internal data from the NHL study. Est (SE) refers to the estimated coefficient and its standard error given by the MLE_{int} and GIM_{opt}.

		CLL(θ_1)	DLBCL(θ_2)	FL(θ_3)	MZL(θ_4)
MLE_{int}	Estimate	0.0654	0.1436	−0.0656	0.1395
	SE	0.0542	0.0504	0.0547	0.0804
	P-value	0.2273	0.0044	0.2309	0.0827
GIM_{opt} using 5 external studies	Estimate	0.0384	0.1372	−0.0877	0.1301
	SE	0.0386	0.0406	0.0446	0.0793
	P-value	0.3206	7.36E-04	0.0493	0.1008
GIM_{opt} using 7 external studies	Estimate	0.0324	0.1238	−0.1167	0.1218
	SE	0.0383	0.0358	0.0389	0.0790
	P-value	0.3974	5.36E-04	0.0027	0.1228
