# Supplementary material for: Improve the model of disease subtype heterogeneity by leveraging external summary data
Source: PLoS Comput Biol. 2023 Jul 12;19(7):e1011236. doi: 10.1371/journal.pcbi.1011236 (PMC10337985; doi:10.1371/journal.pcbi.1011236)
Supplement: S9 Table — Each P-value is for testing the null hypothesis that the PRS has the same effect on different NHL subtypes. (PDF) [file pcbi.1011236.s010.pdf]

Table S9: Disease subtype heterogeneity testing *P*-values based on the NHL study by randomly using 1/3 of the internal data from the NHL study. Each *P*-value is for testing the null hypothesis that the PRS has the same effect on different NHL subtypes.

Comparison	MLE_{int}	GIM_{opt} (5 studies)	GIM_{opt} (7 studies)
CLL vs. DLBCL	0.2129	0.0478	0.0547
CLL vs. FL	0.0485	0.0178	0.0029
CLL vs. MZL	0.4033	0.2743	0.2865
DLBCL vs. FL	0.0010	2.42E-05	5.71E-07
DLBCL vs. MZL	0.9628	0.9328	0.9812
FL vs. MZL	0.0214	0.0112	0.0046
CLL vs. DLBCL vs. FL vs. MZL	0.0072	2.53E-04	6.67E-06
