# Supplementary material for: Improve the model of disease subtype heterogeneity by leveraging external summary data
Source: PLoS Comput Biol. 2023 Jul 12;19(7):e1011236. doi: 10.1371/journal.pcbi.1011236 (PMC10337985; doi:10.1371/journal.pcbi.1011236)
Supplement: S1 Fig — Measurement errors for the PRS are considered at three different levels: none (i.e., w˜i=wi), low (i.e., w˜i∼N(wi,sei2)), and high (i.e., w˜i∼N(wi,16sei2)). (PDF) [file pcbi.1011236.s011.pdf]

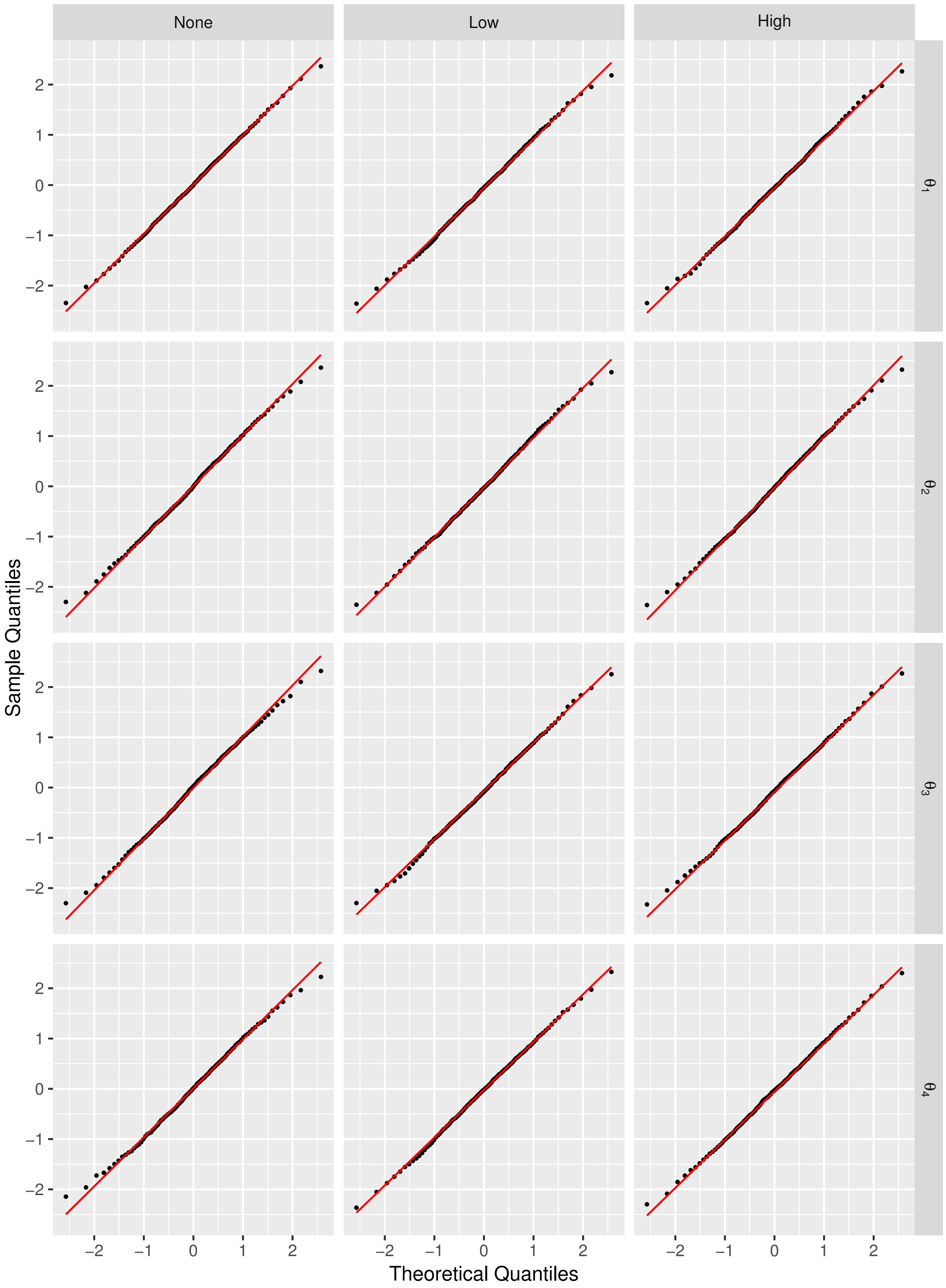


Figure S1: Q-Q plots of $Z$-scores generated by GIM$_{opt}$. Measurement errors for the PRS are considered at three different levels: none (i.e., $\tilde{w}_i = w_i$), low (i.e., $\tilde{w}_i \sim \mathcal{N}(w_i, \mathrm{se}_i^2)$), and high (i.e., $\tilde{w}_i \sim \mathcal{N}(w_i, 16\mathrm{se}_i^2)$).
