# Supplementary material for: Improve the model of disease subtype heterogeneity by leveraging external summary data
Source: PLoS Comput Biol. 2023 Jul 12;19(7):e1011236. doi: 10.1371/journal.pcbi.1011236 (PMC10337985; doi:10.1371/journal.pcbi.1011236)
Supplement: S2 Fig — The minor allele frequency is estimated from the control group within each study. (PDF) [file pcbi.1011236.s012.pdf]

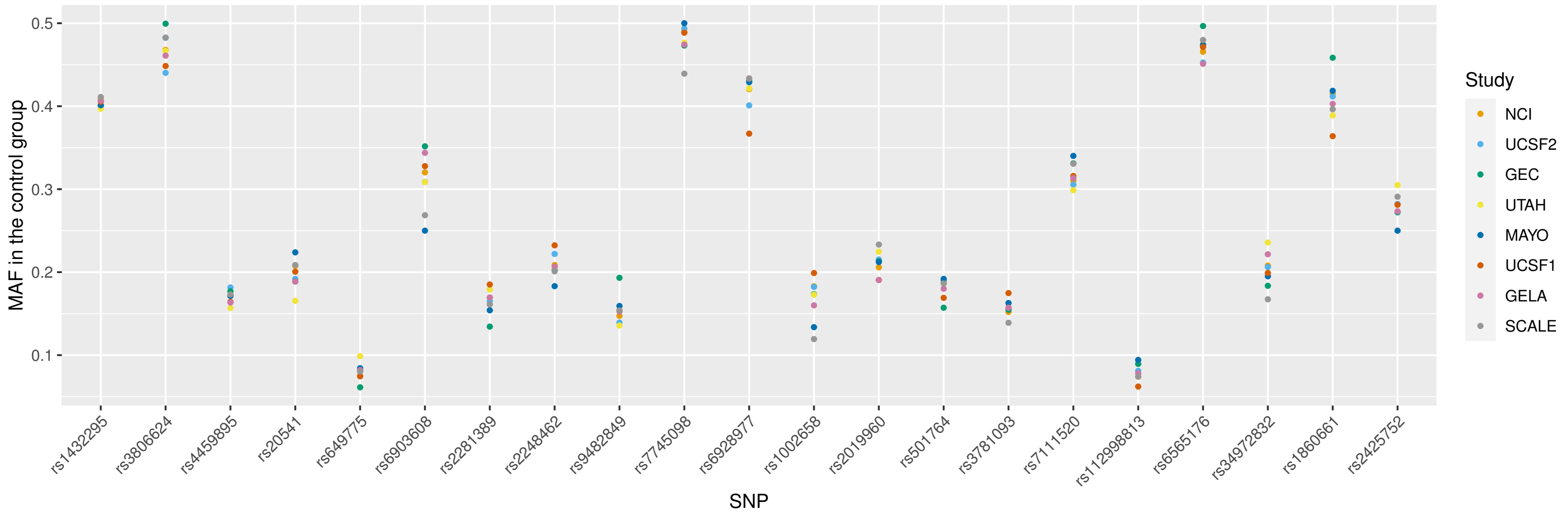

Figure S2: Minor allele frequencies of the 21 SNPs in the eight NHL studies. The minor allele frequency is estimated from the control group within each study.
